# The probiotic *Propionibacterium freudenreichii* as a new adjuvant for TRAIL-based therapy in colorectal cancer

**DOI:** 10.18632/oncotarget.6881

**Published:** 2016-01-11

**Authors:** Fabien J. Cousin, Sandrine Jouan-Lanhouet, Nathalie Théret, Catherine Brenner, Elodie Jouan, Gwénaëlle Le Moigne-Muller, Marie-Thérèse Dimanche-Boitrel, Gwénaël Jan

**Affiliations:** ^1^ INRA, UMR1253 STLO, Science et Technologie du Lait et de l'Œuf, Rennes F-35042, France; ^2^ AGROCAMPUS OUEST, UMR1253 STLO, Rennes F-35042, France; ^3^ CNIEL/Syndifrais, Paris 09 F-75314, France; ^4^ INSERM, UMR1085, Institut de Recherche sur la Santé, l'Environnement et le Travail, Rennes F-35043, France; ^5^ Biosit UMS3080, Université de Rennes 1, Rennes F-35043, France; ^6^ INRIA, UMR6074 IRISA, Rennes F-35042, France; ^7^ INSERM, UMRS1180, LabEx LERMIT, Châtenay-Malabry F-92290, France; ^8^ Université de Paris Sud, Faculté de Pharmacie, Châtenay-Malabry F-92290, France; ^9^ Current address: Research Unit Aliments Bioprocédés Toxicologie Environnements (UR ABTE) EA 4651, Université de Caen Normandie, Caen F-14032, France; ^10^ Current address: Department for Biomedical Molecular Biology, University of Ghent, VIB Inflammation Research Center, Ghent B-9052, Belgium

**Keywords:** TRAIL, RNA microarrays, colon cancer, SCFA, Propionibacterium freudenreichii

## Abstract

TNF-Related Apoptosis-Inducing Ligand (TRAIL) is a well-known apoptosis inducer, which activates the extrinsic death pathway. TRAIL is pro-apoptotic on colon cancer cells, while not cytotoxic towards normal healthy cells. However, its clinical use is limited by cell resistance to cell death which occurs in approximately 50% of cancer cells. Short Chain Fatty Acids (SCFA) are also known to specifically induce apoptosis of cancer cells. In accordance, we have shown that food grade dairy propionibacteria induce intrinsic apoptosis of colon cancer cells, via the production and release of SCFA (propionate and acetate) acting on mitochondria. Here, we investigated possible synergistic effect between *Propionibacterium freudenreichii* and TRAIL. Indeed, we hypothesized that acting on both extrinsic and intrinsic death pathways may exert a synergistic pro-apoptotic effect. Whole transcriptomic analysis demonstrated that propionibacterial supernatant or propionibacterial metabolites (propionate and acetate), in combination with TRAIL, increased pro-apoptotic gene expression (TRAIL-R2/DR5) and decreased anti-apoptotic gene expression (FLIP, XIAP) in HT29 human colon cancer cells. The revealed synergistic pro-apoptotic effect, depending on both death receptors (TRAIL-R1/DR4, TRAIL-R2/DR5) and caspases (caspase-8, -9 and -3) activation, was lethal on cancer cells but not on normal human intestinal epithelial cells (HIEC), and was inhibited by Bcl-2 expression. Finally, milk fermented by *P. freudenreichii* induced HT29 cells apoptosis and enhanced TRAIL cytotoxic activity, as did *P. freudenreichii* DMEM culture supernatants or its SCFA metabolites. These results open new perspectives for food grade *P. freudenreichii*-containing products in order to potentiate TRAIL-based cancer therapy in colorectal cancer.

## INTRODUCTION

Colorectal cancer (CRC), the fourth most frequent cause of cancer deaths worldwide, is tightly linked with lifestyle, including diet [1]. Efforts are made to prevent and/or fight CRC using food components, including prebiotics and probiotics. In this context, dairy Gram positive propionibacteria, as the probiotic species *Propionibacterium freudenreichii* (Pf) [2], were shown to induce apoptosis of colon cancer cells via the intrinsic apoptotic death pathway [3, 4]. These propionibacteria induce apoptosis, via the production of SCFAs, not only *in vitro* but also *in vivo* in human microbiota-associated rats [5]. Interestingly, Pf enhances apoptosis and lowers proliferation only in the context of carcinogenesis induced by dimethylhydrazine (DMH-treated rats) and not in healthy conditions (control rats) [5]. Recently, a first milk fermented exclusively by Pf was obtained and was shown to induce apoptosis in HGT-1 human gastric cancer cells [6]. In this previous study, we have shown that the active compounds, SCFAs, are secreted and recovered in the aqueous phase of the fermented dairy product.

TRAIL, a member of the TNF superfamily, selectively kills transformed and cancer cells, but not most normal cells, by triggering the extrinsic apoptotic death pathway [7]. Indeed, the recombinant human soluble TRAIL (rhTRAIL) is a candidate for cancer therapy [8–10]. TRAIL has anti-tumour activity against a wide variety of tumour cell lines *in vitro* and *in vivo*, including colon cancer [11]. Interestingly, studies have shown that colon carcinomas and high-grade adenomas are more sensitive to TRAIL than normal colonic epithelium, confirming the interest of using TRAIL for colon cancer therapy [12, 13]. TRAIL has also shown efficacy against primary human colon tumour explants in mice [14]. However, most cells derived from human colorectal cancer are resistant against TRAIL-induced apoptosis due to defects in TRAIL signalling machinery [15]. To restore TRAIL sensitivity, combination of TRAIL with chemotherapy, radiotherapy, IFN-γ or other targeted therapies has thus been used [11, 16]. Treatment combining TRAIL with chemotherapy showed for instance a synergistic cytotoxic effect in human cancer cell lines, in tumour xenografts [17, 18], and in patients' colon tumours grown in SCID mice [14], even in drug-resistant ones.

A previous study, particularly, examined the transcriptional response of HT29 cells co-cultivated with probiotic bacterial strains [19]. In this report, the presence of some Gram positive bacteria (bifidobacteria, lactobacilli), other than dairy propionibacteria, modulated expression of genes involved in cytokine-chemokine receptor interactions and NOD signalling, but not apoptosis. In the current work, we compared for the first time the whole genome expression of HT29 cells treated with Pf culture supernatant (SN) or a mixture of its major metabolites (propionate and acetate, C3/C2), in combination or not with TRAIL. Analysis of microarrays demonstrated that SN or C3/C2 in combination with TRAIL modulated the expression of apoptosis related genes. This effect was confirmed in cytotoxic and apoptotic cellular assays showing a synergistic pro-apoptotic action between Pf culture supernatant (or C3/C2) and TRAIL. Finally, a milk fermented by Pf exhibited the same pro-apoptotic properties as Pf culture supernatant and sensitized colon cancer cells to TRAIL.

## RESULTS

### Apoptosis signaling pathway is modulated by *P. freudenreichii* culture supernatant, metabolites (propionate/acetate) or TRAIL alone and by their combinations

**Figure 1:**
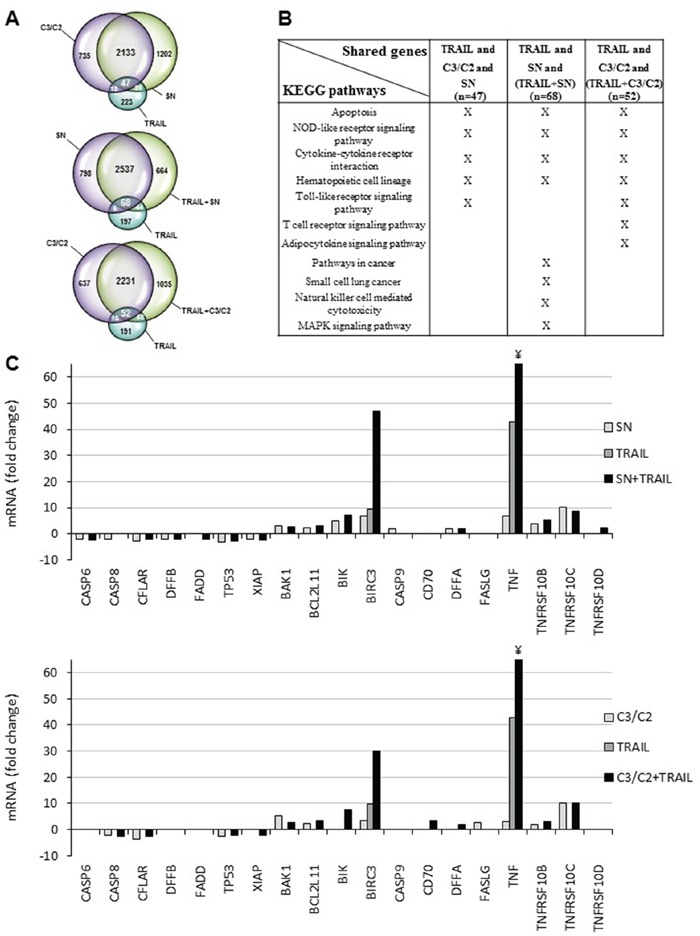
Transcriptomic analyses of HT29 cells treated with TRAIL combined or not with propionibacterial supernatant or metabolites HT29 cells were left untreated or treated for 6 h with either TRAIL-Flag (100 ng/ml + 2 μg/ml anti-Flag) or *P. freudenreichii* DMEM culture supernatant (SN 1/2) or a SCFAs mixture (C3/C2 containing 30 mM propionate and 15 mM acetate) or combinations as indicated. **A.** Venn diagrams of genes up-regulated in HT29 cells treated with single or combined treatments. Complete lists of up-regulated genes are provided as supplementary Table 1. **B.** KEGG pathway enrichment analysis of genes shared by treatments either with TRAIL, C3/C2 and SN (n=47) or TRAIL, SN and TRAIL combined with SN (n=68), or TRAIL, C3/C2 and TRAIL combined with C3/C2 (n=52). Complete list of KEGG pathways for each treatment are provided as supplementary Table 2. **C.** Effect of cell treatment associating TRAIL with propionibacterial supernatant or metabolites on expression of apoptosis-related genes. mRNA expression levels of apoptosis-related genes were extracted from microarray data and plotted as histograms. Data are expressed as fold increase in treated cells compared to untreated cells (p<0.05). For TNF mRNA, the value is approximately 100 for the symbol ¥.

A whole transcriptome analysis was carried out using microarrays, to elucidate the response of HT29 human colon cancer cells to a 6 h treatment combining TRAIL (100 ng/ml + 2 μg/ml anti-Flag M2 antibody) with propionibacterial culture supernatant (SN diluted to 1/2) or a mixture of propionate (30 mM) and acetate (15 mM) (C3/C2, the major metabolites used in the amounts present in the diluted SN). As illustrated by Venn diagrams (Figure 1A and see Supplementary Table 1 for lists of genes), most of the genes induced by SN were also induced by C3/C2 (2180 genes) suggesting a similar effect due to the presence of acetate and propionate in the propionibacterial culture supernatant. Treatment by TRAIL led to a much limited number of over-expressed genes (314 genes), while co-treatments induced expression of 3313 and 3376 genes (for TRAIL+SN and TRAIL+C3/C2, respectively).

To further explore the significance of transcriptomic changes triggered by co-treatments, we investigated the functional annotation of overexpressed genes in the co-treatments compared to the single treatments. We used FatiGo, a web tool from the Babelomics suite, to find significant KEGG pathways associations with groups of genes [20]. Importantly, genes shared by all conditions (*i.e* single treatments or combinations), are characterized by common KEGG pathways including apoptosis, NOD-like receptors and cytokine-cytokine receptors interaction (Figure 1B), known to play a role in immune response. Of note, genes from the combination TRAIL with C3/C2 showed an important increase in the number of KEGG pathways compared with genes from single treatment, suggesting a functional synergy between C3/C2 and TRAIL treatments (Supplementary Table 2).

**Figure 2:**
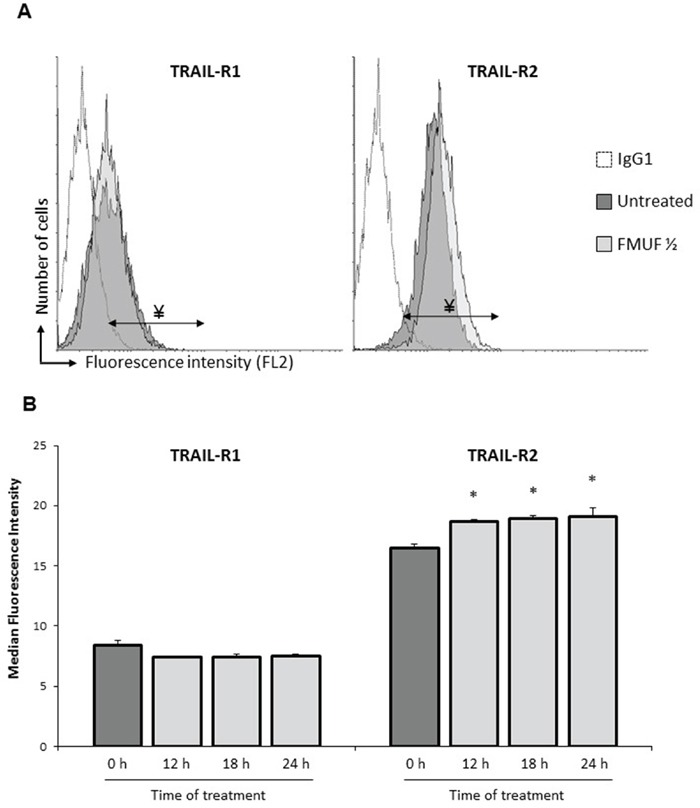
FMUF treatment increases cell surface expression of death receptor TRAIL-R2 HT29 cells were left untreated or were treated with FMUF ½ for 12, 18 and 24 h. **A.** Cell surface expression of TRAIL-R1 or TRAIL-R2 was analyzed by flow cytometry in HT29 cells after treatment without (dark gray histogram) or with FMUF ½ (light gray histogram). Empty histogram represented background fluorescence obtained with secondary antibody alone. Fluorescence histograms were shown for a 12 h time treatment. **B.** Presented values correspond to the median fluorescence intensity for each time treatment. *n*=3, data are means ± SD, * *P*<0.05, treated cells versus control (0 h).

Focusing on apoptosis, we next extracted expression data for 19 apoptosis-related genes. As shown in Figure 1C, the association of TRAIL with either SN or C3/C2 clearly negatively or positively modulated the expression of pro- or anti- apoptotic genes. More specifically, the expression of pro-apoptotic (TNFRSF10B) or anti-apoptotic (CFLAR, XIAP) genes, related to the TRAIL death receptor pathway, were increased or decreased, respectively, in cells treated by TRAIL combined with either SN or C3/C2 (Figure 1C and Table 1). These results thereby demonstrated the efficiency of co-treatments in relationship with apoptosis. Interestingly, propionibacterial SN or C3/C2 metabolites, alone, induced TNFRSF10B (alias TRAIL-R2/DR5) expression, a TRAIL death receptor (Figure 1C and Table 1). TNFRSF10B overexpression was thus confirmed, at the protein level, by characterizing TRAIL-R1 or TRAIL-R2 membrane expression on HT29 cells by flow cytometry after their treatment with the supernatant of Pf-fermented milk ultrafiltrate (FMUF ½), prepared as previously described [6]. An increase in TRAIL-R2 membrane expression was thus observed in HT29 cells after a 12 h treatment with FMUF (Figure 2A), and up to 24 h (Figure 2B). Together, these data suggest that SCFA metabolites (also present in SN or FMUF) may potentiate TRAIL-induced apoptotic effect.

**Table 1:** Gene expression from transcriptomic analyses

Gene symbol	C3/C2vsNT	SNvsNT	TRAILvsNT	(C3/C2+TRAIL)vsNT	(SN+TRAIL)vsNT
CDKN1A (p21)	+5.5	+18.7	+2.5	+13.5	+23
CFLAR (FLIPL)	−3.8	−2.7	NS	−2.5	−2.13
XIAP	NS	−2.1	NS	−2.3	−2.57
TNFRSF10B (DR5)	+2.02	+3.82	NS	+2.89	+5.39
BAX	NS	NS	NS	NS	NS
MCL1	NS	NS	NS	NS	NS
BCL21 (Bcl-XL)	NS	NS	NS	NS	NS
BAK1 (Bak)	+2.09	+2.99	NS	+2.5	+2.9

Values are expressed as fold change (+) in case of increased gene expression or (−) in case of decreased gene expression. NS: non significant.

### *P. freudenreichii* metabolites enhance TRAIL cytotoxicity in HT29 or HCT116 human colon cancer cells compared to HIEC human healthy intestinal epithelial cells

**Figure 3:**
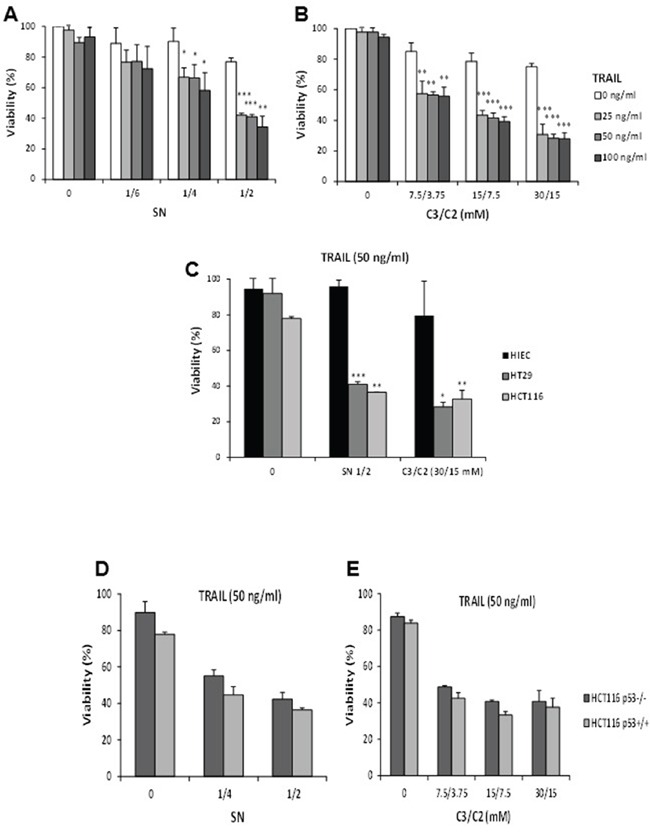
Cytotoxicity of TRAIL in combination or not with propionibacterial supernatant or metabolites in different cell lines HT29 cells were left untreated (0) or were treated for 24 h with a combination of increased concentrations of TRAIL with increased dilutions of a *P. freudenreichii* DMEM culture supernatant (SN) **A.** or increased concentrations of the SCFAs C3/C2 **B.** Viability of HT29 cells was measured by a methylene blue assay as indicated in materials and methods. *n*=3, data are means ± SD, *P<0.05, **P<0.01, ***P<0.001 TRAIL versus TRAIL+SN or TRAIL versus TRAIL+C3/C2. **C.** HT29, HCT116 or HIEC cells were left untreated (0) or were treated with a combination of 50 ng/ml TRAIL with a *P. freudenreichii* DMEM culture supernatant (SN ½) or with the SCFAs C3/C2 (30/15 mM). Cell viability was measured using a methylene blue assay. *n*=3, data are means ± SD, *P<0.05, **P<0.01, ***P<0.001 HIEC versus HT29 or HIEC versus HCT116. p53^+/+^and p53^−/−^ HCT116 cells were left untreated (0) or were treated for 24 h with TRAIL (50 ng/ml) in combination with increased dilutions of a *P. freudenreichii* DMEM culture supernatant (SN) **D.** or increased concentrations of the SCFAs (C3/C2) **E.** Viability of HCT116 cells was measured by a methylene blue assay as indicated in materials and methods. *n*=3, data are means ± SD.

Since the microarray analysis suggests an enhanced pro-apoptotic effect of TRAIL in combination with SN or C3/C2 in HT29 cells, we further analysed the cytotoxic effect of these treatments using a methylene blue assay [6]. Up to 100 ng/ml, TRAIL had a low cytotoxic effect on its own in HT29 cells, with less than 10% of viability loss after 24 h, as shown in Figure 3A and 3B (0-5% of cell death for 25 ng/ml). Besides, propionibacterial supernatant at the highest concentration (½) only killed 23% after 24 h. However, a synergistic effect was observed for 25 ng/ml of TRAIL in combination with SN ½, leading to 57% of cell death (Figure 3A). Similar results were observed for TRAIL in combination with C3/C2 (Figure 3B).

However, HIEC human healthy epithelial colon cells were less susceptible than HT29 or HCT116 cells to cell death induction by combined treatments (Figure 3C), suggesting that normal cells would be spared from the toxicity of TRAIL in combination with SN or C3/C2. Expression or not of p53 did not modify the cytotoxicity of TRAIL/SN or TRAIL/C3/C2 combinations in HCT116 (Figure 3D and 3E).

### Bcl-2 is a resistance factor towards treatment induced by combination of TRAIL with SN or C3/C2

**Figure 4:**
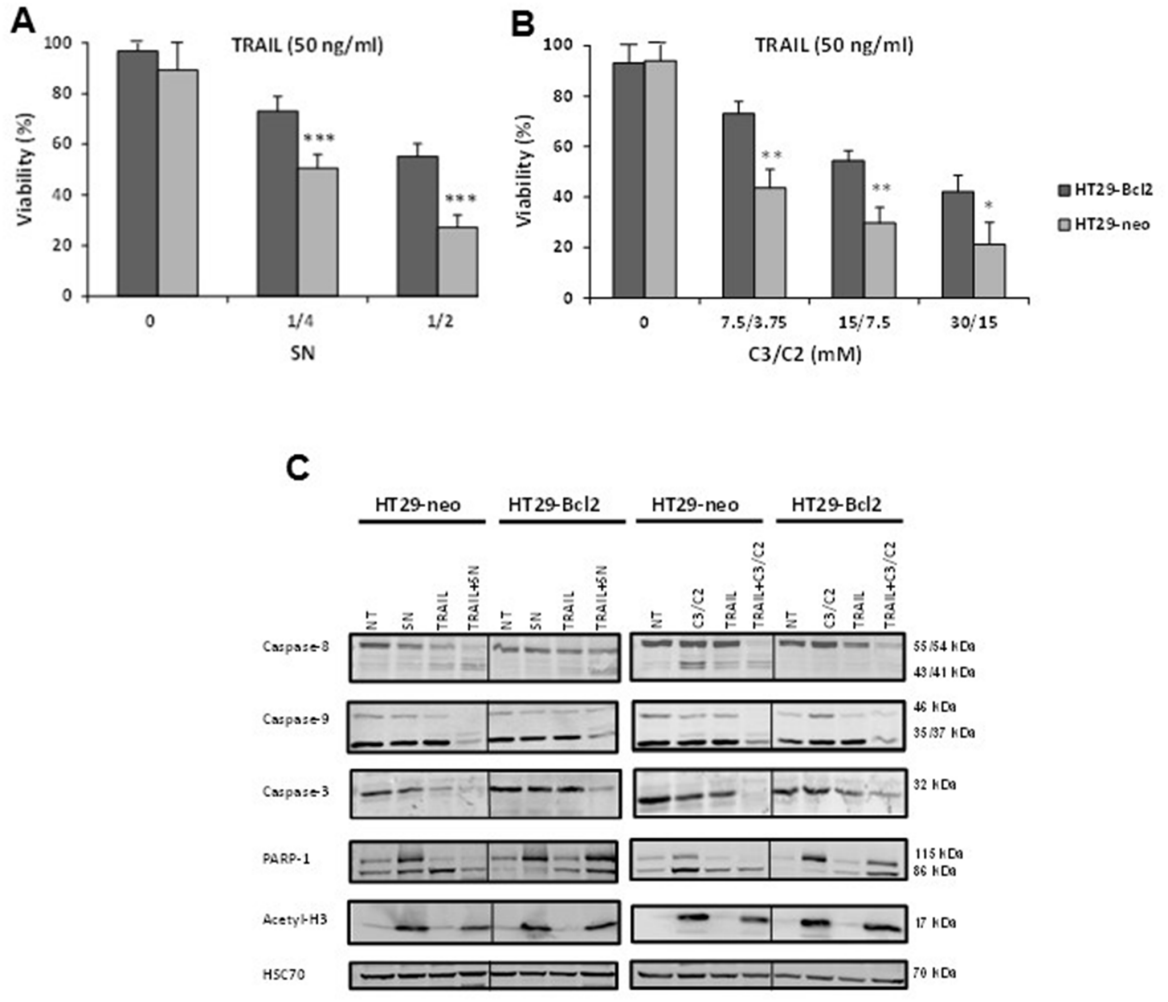
Bcl-2 is a resistance factor towards treatment induced by combination of TRAIL with SN or C3/C2 HT29 cells expressing or not Bcl-2 (HT29-Bcl2 and HT29-neo) were left untreated (0) or were treated for 24 h with a combination of increased concentrations of TRAIL with increased dilutions of a *P. freudenreichii* DMEM culture supernatant (SN) **A.** or increased concentrations of the SCFAs C3/C2 **B.** Viability was measured by a methylene blue assay. *n*=3, data are means ± SD, *P<0.05, **P<0.01, ***P<0.001 HT29-Bcl2 versus HT29-neo. **C.** Caspases-8, -9 and -3, PARP-1 and Acetyl-H3 were analyzed by western blotting in cell extracts of HT29 cells, either expressing Bcl-2 (HT29-Bcl2) or not (HT29-neo), treated or not (NT) with TRAIL (100 ng/ml), SN (1/2), C3/C2 (30/15 mM) or combinations. Antibodies directed against Hsc70 were used as a loading control.

We compared the cytotoxicity of the combined treatments (TRAIL with SN or C3/C2) in HT29 cells that do not constitutively express Bcl-2 (HT29-neo) and in HT29 cells stably transfected with Bcl-2 (HT29-Bcl2) [21]. After a 24 h treatment, HT29-Bcl2 cells presented a decreased cell sensitivity to combination of TRAIL with SN or C3/C2, in comparison with HT29-neo cells (Figure 4A and 4B).

Then, we performed western blot experiments (Figure 4C). Activation of caspase-8, -9 and -3 was observed in HT29-neo cells exposed to TRAIL/SN or TRAIL/C3/C2 combination, as evidenced by the decreased amount of the proforms of caspase-3, -8 and -9 which are no more detected at the end of the 24 h treatment and the appearance of cleavage products of caspase-8 (41/43 kDa) and caspase-9 (35/37 kDa), as well as the complete cleavage of PARP-1, a substrate of caspase-3. In HT29-Bcl2 cells treated with TRAIL/SN or TRAIL/C3/C2, the proforms of caspases-8, -9 and -3 were still detected by western blot as well as the uncleaved PARP-1 (Figure 4C) at the end of the 24 h treatment, suggesting that expression of Bcl-2 delays the activation of caspases-8, -9 and -3, leading to TRAIL/SN or TRAIL/C3/C2 resistance (Figure 4A and 4B). As it has been shown that SCFA can modulate histone acetylation [22], we demonstrated that treatment of HT29 cells with SN or C3/C2 alone or in combination with TRAIL led to the histone H3 acetylation which may be indicative of a histone deacetylase inhibitory activity present in the supernatant (SN) or the mixture of SCFA (C3/C2) (Figure 4C).

### TRAIL/C3/C2 synergistic cytotoxicity involves the activation of both extrinsic and intrinsic apoptotic pathways

**Figure 5:**
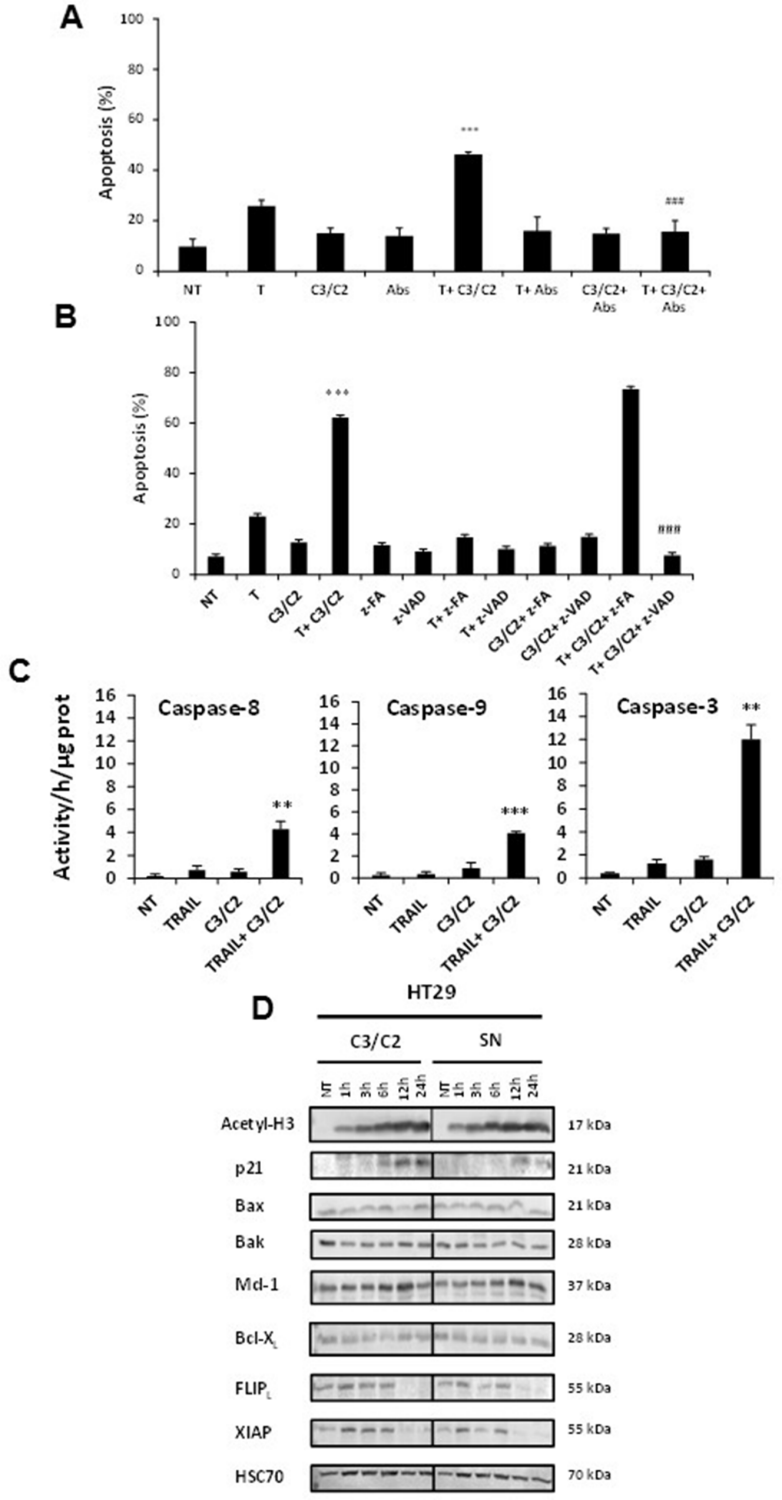
Implication of both extrinsic and intrinsic pathways in synergistic effect HT29 cells were left untreated (NT) or treated for 24 h with TRAIL (T, 50 ng/ml), C3/C2 (30/15 mM) or combination. **A.** Antibodies (Abs) against DR4 and DR5 were used to antagonize the pro-apoptotic effect of these treatments. *n*=3, data are means ± SD, ***P<0.001 TRAIL versus TRAIL+C3/C2, ^###^*P*<0.001 TRAIL+C3/C2 versus TRAIL+C3/C2+Abs. **B.** A pan-caspase inhibitor (z-VAD) and a caspase inhibitor negative control (z-FA) were used to antagonize the pro-apoptotic effect of these treatments. *n*=3, data are means ± SD, ***P<0.001 TRAIL versus TRAIL+C3/C2, ^###^*P*<0.001 TRAIL+C3/C2 versus TRAIL+C3/C2+z-VAD. **C.** Caspase-8, caspase-9 and caspase-3 activities were quantified in HT29 treated cells as indicated in the materials and methods. Caspases specific activities of lysates from cells treated as above were determined using specific fluorescent peptides. *n*=3, data are means ± SD, **P<0.01, ***P<0.001 TRAIL versus TRAIL+C3/C2. **D.** Expression of key proteins was analyzed by western blotting in HT29 cells treated or not (NT) with SN or C3/C2. Antibodies directed against Hsc70 were used as a loading control.

The percentage of HT29 apoptotic cells (10%) following a 24 h treatment with 30/15 mM C3/C2, as determined by Hoechst 33342 and propidium iodide double staining, was similar to the untreated control (NT) (Figure 5A), in accordance with the very low cytotoxicity of these concentrations, as detected in viability assay (Figure 3B). Treatment with TRAIL alone induced 20% of apoptotic cells. However, combination of TRAIL with C3/C2 led to an increase in apoptosis, with 45% of apoptotic cells (Figure 5A). Blocking TRAIL death receptors by using antagonistic antibodies against DR4 and DR5 (Abs) led to an almost complete inhibition of apoptosis induced by TRAIL and C3/C2 combination, showing activation of the extrinsic death pathway. Control treatment with these antibodies alone had no effect (Figure 5A).

Moreover, Figure 5B showed that z-VAD-fmk, a pan-caspase inhibitor, drastically reduced the synergistic apoptotic effect of TRAIL with C3/C2 in HT29 cells, while a caspase inhibitor negative control, z-FA-fmk had no effect, confirming the role of caspases in this cell death pathway. Finally, Figure 5C evidenced an enhanced activation of caspase-8, -9 and -3, as a result of co-treatment, in comparison with treatment with TRAIL or C3/C2 alone, which was in agreement with Western blot data (Figure 4C). Activation of caspase-8 or caspase-9 by co-treatment evidenced activation of extrinsic or intrinsic death pathway, respectively. Next, we assessed the impact of propionibacterial metabolites (C3/C2) or SN on expression of some key regulatory proteins of apoptosis signalling machinery (Bax, Bak, p21, Mcl-1, Bcl-X_L_, FLIP_L_, XIAP). Western blot analysis showed enhanced cellular contents of acetylated histone H3 and p21 in a time-dependent manner following treatment with C3/C2 or SN in HT29 cells (Figure 5D), suggesting inhibition of histone deacetylase. Moreover, the expression of two inhibitors of the extrinsic and the intrinsic apoptotic pathway FLIP_L_ and XIAP respectively, was decreased by C3/C2 or SN treatment (Figure 5D), which could explain cells sensitivity when C3/C2 or SN are combined with TRAIL. These data are in accordance with microarray analysis showing an increase in mRNA level expression of CDKN1A (p21) or a decrease in mRNA level expression of CFLAR (FLIP_L)_ and XIAP after a 6 h treatment with SN or C3/C2 treatment (Table 1). Some small changes in the expression of Bax, Bak, Mcl-1 and Bcl-X_L_ were observed after C3/C2 or SN treatment (Figure 5D and Table 1).

These results showing that propionibacterial metabolites or SN sensitize cells to TRAIL and enhance its effect, we thus hypothesized that a dairy product, fermented exclusively by *P. freudenreichii*, might sensitize colon cancer cells towards TRAIL.

### *P. freudenreichii*- fermented milk or fermented milk ultrafiltrate induces colon cancer cells apoptosis

**Figure 6:**
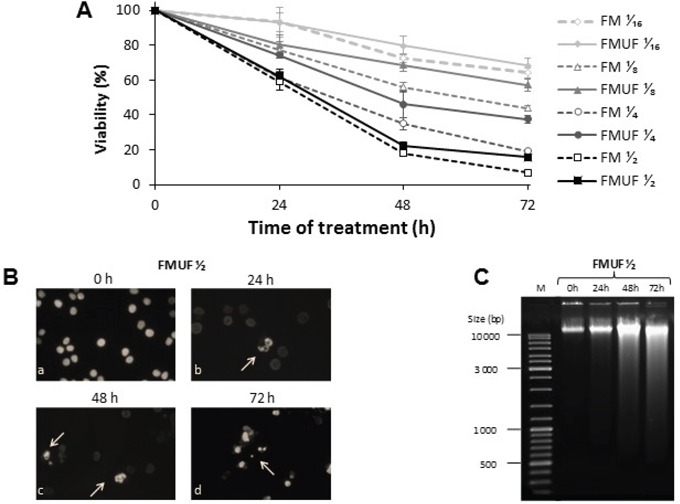
Cytotoxicity of *P. freudenreichii*-fermented milk and of *P. freudenreichii*-fermented milk ultrafiltrate **A.** HT29 cells were left untreated or were treated with increased dilutions of fermented milk (FM) supernatant or fermented milk ultrafiltrate (FMUF) supernatant for 24, 48 and 72 h. Cells viability was measured using a methylene blue assay as indicated in materials and methods. **B.** Cells treated as above were stained with Hoechst 33342 prior to fluorescence microscopy. Arrows indicate chromatin condensation. **C.** HT29 genomic DNA was extracted and analyzed in 1 % agarose gel as described in [6].

A milk (FM) and a milk ultrafiltrate (FMUF, i.e. milk devoid of casein), both fermented exclusively by *P. freudenreichii*, were prepared as previously described by Cousin et al [6, 23, 24]. Supernatants of both products, either in the presence or absence of milk caseins, contained similar concentration of C3 (close to 60 mM) and of C2 (close to 30 mM), and induced cell death in dose- and time-dependent manner in HT29 cells (Figure 6A). FMUF gave similar results as FM and was clear while FM was turbid, so that following experiments were performed using FMUF.

**Figure 7:**
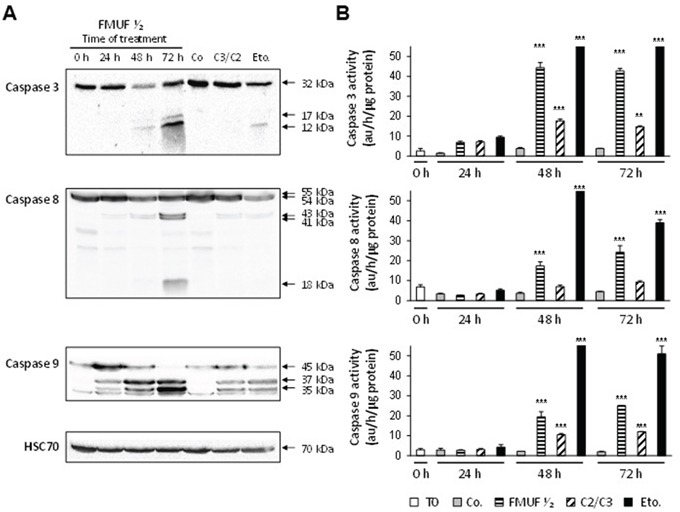
*P. freudenreichii*-fermented milk ultrafiltrate activates caspases-3, -8 and -9 in HT29 cells HT29 cells were treated or not (0 h) with *P. freudenreichii* fermented milk ultrafiltrate (FMUF ½) for 24, 48 and 72 h. **A.** Processing of caspase-3, -8 and -9 was followed by western blotting in lysates of HT29 cells treated with supernatant of *P. freudenreichii* fermented milk ultrafiltrate (FMUF ½). Non-fermented milk ultrafiltrate (Co, 48 h), a mixture of acetate and propionate (C3/C2, 30/15 mM, 48 h) and etoposide (Eto, 100 μM, 48 h) were used as controls. Antibodies directed against Hsc70 were used as a loading control. **B.** Caspases-3, -8 and -9 activities were measured in lysates from HT29 cells as described in materials and methods. *n*=3, data are means ± SD, **P<0.01, ***P<0.001 treated versus control (0 h).

In accordance with effects reported for propionibacterial metabolites C3/C2, FMUF induced a caspase-dependent apoptosis in HT29 cells (Figure 7). Western blotting analysis revealed cleaved forms for caspases -3, -8 and -9 after 72 h of treatment with FMUF ½ (Figure 7A). Moreover, activities of caspases-3, -8 and -9 were quantified in HT29 cell extracts (Figure 7B). Unfermented milk ultrafiltrate (Co) did not induce any caspase activity. By contrast, FMUF induced activation of the 3 caspases detected at 48 h and 72 h, as did C3/C2 or etoposide (Eto.), used as an apoptosis positive control (Figure 7A and 7B).

**Figure 8:**
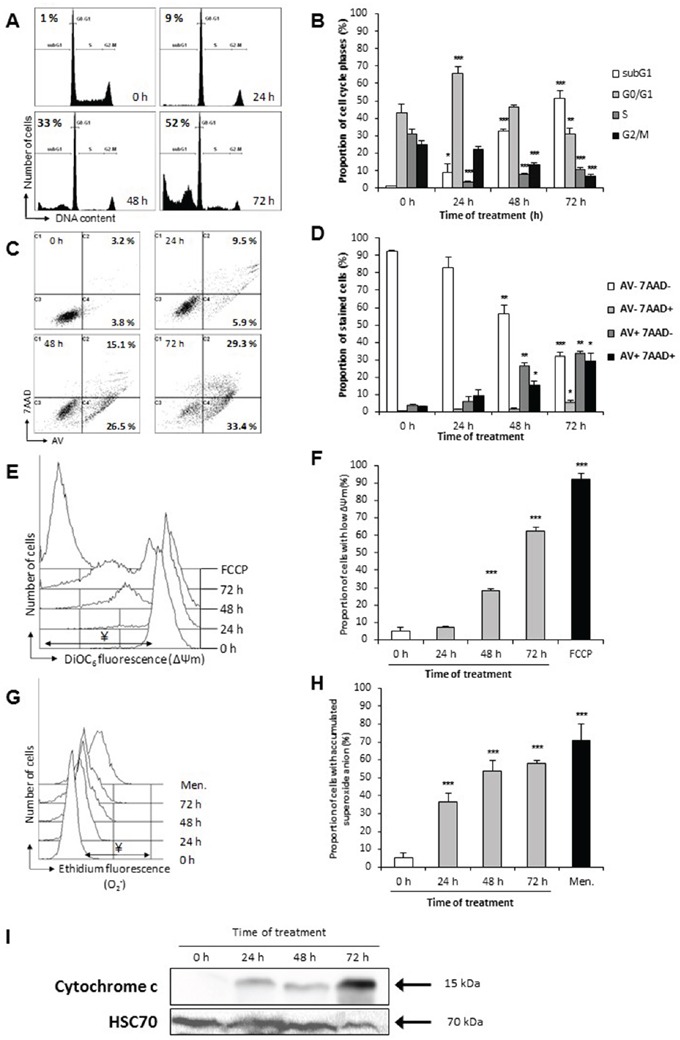
*P. freudenreichii*-fermented milk ultrafiltrate induces apoptosis via the mitochondria pathway HT29 cells were treated as in Figure 7. **A.** Fermented milk ultrafiltrate-induced changes in cell cycle phases. DNA content of HT29 cells was analyzed by flow cytometry after propidium iodide staining. The percentage of the cell population with sub-G1 DNA content, indicative of apoptosis, is indicated (one representative experiment). **B.** Proportion of each cell subsets (sub-G1, G0/G1, S, G2/M), within the total cell population, is shown for each time of treatment. *n*=3, data are means ± SD, *P<0.05, **P<0.01, ***P<0.001 treated cells versus control (0 h). **C.** Flow cytometry kinetic analysis of cell death in HT29 cells treated with *P. freudenreichii* fermented milk ultrafiltrate (FMUF ½ dilution in DMEM). A representative experiment of Annexin V/7-AAD staining of HT29 cells at each time of treatment is shown, with proportions of Annexin V positive cells (AV+; apoptotic cells). **D.** Quantitative FACS analysis of Annexin V-FITC (AV) binding to HT29 cells was performed after counterstaining with 7-aminoactinomycin-D (7AAD). Presented values correspond to the proportion of each cell subsets, within the total cell population, for each treatment time. *n*=3, data are means ± SD, *P<0.05, **P<0.01, ***P<0.001 treated cells versus control (0 h). **E.** Flow cytometry kinetic analysis of mitochondrial inner membrane (ΔΨm) dissipation with DiOC_6_ staining. An overlay view of one representative experiment is shown. The decoupling FCCP (50 μM, 20 min) was used as positive control. **F.** Quantitative analyses of ΔΨm loss in 3 independent experiments. Values are represented as a proportion of cells with decreased ΔΨm, within the total cell population, for each treatment. *n*=3, data are means ± SD, ***P<0.001, treated versus control (0 h). **G.** Flow cytometry kinetic analysis of anion superoxide (O_2_^.−^) accumulation with DHE staining. An overlay of one representative experiment of ROS detection at each time of treatment is shown. Cells were stained with DHE and analyzed by flow cytometry. The pro-oxidant menadione (Men., 100 μM, 15 min) was used as positive control. **H.** Values are represented as a proportion of cells with increased ROS (increase of fluorescence intensity), within the total cell population, for each treatment. *n*=3, data are means ± SD, ***P<0.001, treated versus control (0 h). **I.** Cytochrome *c* relocation over the whole treatment period with *P. freudenreichii*-fermented milk ultrafiltrate supernatant ½ was assessed by western blot analysis of cytoplasm-enrich fractions. Antibodies directed against Hsc70 were used as a loading control.

Modifications of DNA content was also monitored by flow cytometry after propidium iodide staining. Up to 52% of cells were in the sub-G1 fraction after 72 h of treatment, while shorter treatment time resulted in enhanced G0/G1 population (70 % at 24 h of treatment) (Figure 8A and 8B), these data were in accordance with the observed chromatin fragmentation in apoptotic nuclei resulting in DNA degradation (Figure 6B and 6C).

Phosphatidylserine translocation from the inner to the outer leaflet of HT29 plasma membrane was monitored by staining cells with Annexin V (AV) and 7-AAD prior to flow cytometry fluorescence analysis (Figure 8C and 8D). Up to 63% of the cells were stained by AV after 72 h of treatment by FMUF diluted ½ in DMEM, in comparison with 7% of AV positive cells in untreated cells (0 h), suggesting apoptosis induction.

The mitochondrial inner membrane potential ΔΨm, a crucial parameter in intrinsic apoptosis induction, was also monitored. We observed an increase in cells exhibiting a low ΔΨm upon FMUF treatment with time (62% after 72 h) (Figure 8E and 8F). Accumulation of anion superoxide (O_2_^.−^) was followed using ethidium staining and flow cytometry analysis (Figure 8G and 8H), leading to 58% of cells with accumulated superoxide anion after 72 h of treatment with FMUF. Finally, immunoblotting examination of a cytoplasmic-enriched fraction for the presence of cytochrome *c* revealed a strong release of this protein from mitochondria into the cytoplasm upon time treatment with FMUF (Figure 8I).

Altogether, these results indicate that propionibacterial metabolites contained in FMUF induced cell cycle arrest and typical hallmarks of intrinsic apoptosis.

### *P. freudenreichii*- fermented milk ultrafiltrate enhances TRAIL cytotoxicity

**Figure 9:**
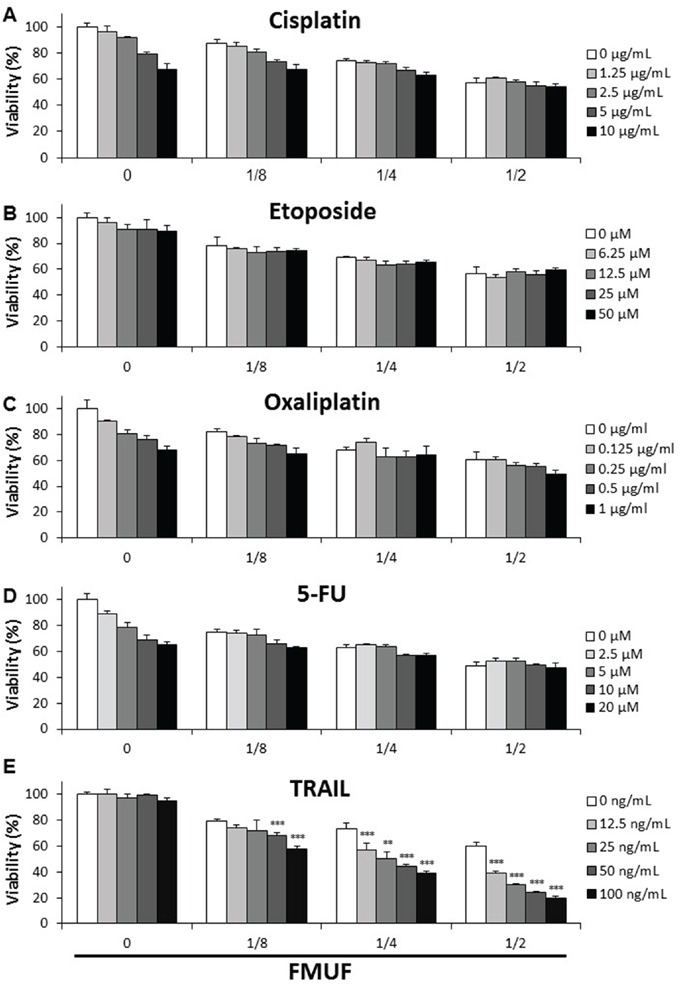
*P. freudenreichii*-fermented milk ultrafiltrate enhances the cytotoxic effect of TRAIL HT29 cells were treated for 24 h with increased concentrations of cisplatin **A.**, etoposide **B.**, oxaliplatin **C.**, 5-fluorouracil (5-FU) **D.** or TRAIL **E.** in combination or not with increased dilutions of *P. freudenreichii* fermented milk ultrafiltrate (FMUF) supernatant. Cells viability was measured using a methylene blue assay as indicated in materials and methods. *n*=3, data are means ± SD, **P<0.01, ***P<0.001, sum of the single treatments FMUF and TRAIL versus the combined treatment of TRAIL and FMUF.

We investigated the combined cytotoxic effects of FMUF and several molecules considered for colon cancer chemotherapy. As shown on Figure 9, cisplatin, etoposide, oxaliplatin and 5-FU induced a dose-dependent cell death in HT29 cells in a 24 h treatment, which was little affected by the addition of FMUF. TRAIL alone had little effect on cell viability, whatever the tested doses, in 24 h. Combination of TRAIL with FMUF killed HT29 cells in a dose-dependent manner. Moreover, whatever the TRAIL concentration, FMUF enhanced its cytotoxicity.

## DISCUSSION

New tools are needed to prevent and/or help treating CRC acting on diet and efficacy of treatments. TRAIL induces apoptosis of colon cancer cells both *in vitro* and *in vivo*, via the extrinsic pathway. Clinical trials demonstrated that rhTRAIL needs to be used in combination with other chemotherapeutic drugs to expect clinical responses [25]. Dairy propionibacteria, including *Propionibacterium freudenreichii*, a food-grade probiotic bacterium generally recognized as safe, were previously shown to induce apoptosis *in vitro* via the intrinsic death pathway [3] and also *in vivo* in the context of carcinogenesis induced by DMH [5]. In this work, we show for the first time that HT29 human colon cancer cells co-treated with TRAIL and a supernatant of propionibacteria, or a mixture of propionate and acetate (C3/C2), displayed quite similar transcription modulation pattern, with changes in expression of genes involved in apoptosis, suggesting a potential synergistic pro-apoptotic effect of these combinations. Among the pro-apoptotic genes, TNFRSF10B (alias TRAIL-R2/DR5) was increased after treatment with SN (+ 3.82 fold change) or C3/C2 (+ 2.02 fold change). Moreover, TRAIL-R2 expression was shown to be increased on cell membrane of HT29 cells after treatment with supernatant of Pf-fermented milk ultrafiltrate (FMUF), a dairy product containing C3/C2. These data are in accordance with the study of Morales et al., where butyrate, another SCFA, induced overexpression of the TRAIL-R2 in human leukemic cells [26]. TRAIL-R2 is predominantly regulated at the transcriptional level [27]. Such an increase in TRAIL-R2 expression could participate to TRAIL sensitization induced by SN, C3/C2 or FMUF [28]. The modulation of the expression of NOD-like receptors and cytokine-cytokine receptors at the mRNA level was also observed in agreement with previous data obtained with HT29 cells after adhesion of probiotic microbes [19] and with the immunomodulatory properties of propionibacteria [29].

The cytotoxic and apoptotic assays confirmed transcriptomic data showing that combination of TRAIL with SN or C3/C2 has a synergistic cytotoxic activity in HT29, with increased cytotoxicity via caspase-dependent apoptosis, in agreement with the apoptotic effect of TRAIL [8] and of acetate and propionate [3, 30, 31] in various cell lines. The cell death pathway triggered by the combinations involved TRAIL death receptors, caspase-3, -8 and -9 activation and mitochondria, since the use of blocking antibodies directed against DR4 or DR5, of a pan-caspase inhibitor (z-VAD-fmk) or of Bcl-2 expression inhibited the pro-apoptotic effect of combination of TRAIL with SN or C3/C2 in HT29 cells. Finally, the very low cytotoxic effect of TRAIL in combination with SN or C3/C2 on normal human intestinal epithelial cells (HIEC) suggests a possible therapeutic application in humans. However, overexpression of Bcl-2 in human colon tumors [32] could be a resistance factor to this kind of co-treatment.

Interestingly, treatment with SN or a mixture of propionate and acetate (C3/C2) induced histone H3 acetylation which has already been reported for mixtures of SCFAs including butyrate, propionate and acetate [33]. Our data demonstrated that a ratio of propionate and acetate (30/15 mM) found in propionibacterial supernatant (SN), is also able to acetylate histone H3 in the same extent as SN. Histone hyperacetylation has been linked to inhibition of histone deacetylase (HDAC) by butyrate [34] leading to growth inhibition [35] and cell-cycle arrest [36] with induction of p21, a CDK (Cyclin-Dependent Kinase) inhibitor [37]. Accordingly, our results clearly showed that p21 protein expression was induced in HT29 cells treated with SN or C3/C2 and correlated with increased histone H3 acetylation, suggesting that SN or mixture of C3/C2 presents a histone deacetylase inhibitory action as butyrate [38]. In parallel, after a 12 h or 24 h treatment with SN or C3/C2, expression of two inhibitors of apoptosis, FLIP_L_ and XIAP, was decreased and could account for increased apoptosis induced by TRAIL in combination with SN or C3/C2 since these inhibitors are both key regulators of extrinsic and intrinsic cell death pathways, respectively [39]. The protein expression levels of p21, FLIP_L_ and XIAP, detected by western blot, were correlated with mRNA levels quantified by microarray analyses showing, after a 6 h treatment, an increase in mRNA levels coding for p21 (CDKN1A) by SN (+18.7 fold change) or C3/C2 (+5.5 fold change) and a decrease in mRNA levels coding for CFLAR (FLIP_L_) by SN (−2.7 fold change) or by C3/C2 (−3.8 fold change) or for XIAP by SN (−2.1 fold change). Altogether, these results suggest that SN or C3/C2 could sensitize colon cancer cells to TRAIL by an anti-proliferative effect via induction of the cell cycle p21 inhibitor leading to a G0/G1 arrest and also by a pro-apoptotic effect via increased expression of DR5 and decreased expression of apoptosis inhibitors (XIAP, FLIP_L_). Such an inhibitory effect on FLIP_L_ expression has already been shown for butyrate treatment in human colon cancer cells, explaining sensitization of these cells to TRAIL-induced apoptosis [40]. Downregulation of FLIP_L_ or XIAP sensitized human colon cancer cells to TRAIL-induced apoptosis [39]. These modifications of specific gene expression is a very interesting point in the objective of cancer therapy since HDAC inhibitors have been proposed to be associated to TRAIL for cancer treatment (review in [41]). A report from the FP6-ONCODEATH research consortium has pointed out the interest to use TRAIL with other drugs, particularly HDAC inhibitors [42].

The link established between diet and the occurrence of CRC strongly suggests that modulation of the diet may play a role in the prevention of CRC and/or in helping its treatment. Trials using probiotic products in the context of CRC treatment have been conducted and revealed a beneficial impact on chemotherapy side effects [43] and on post-operative infections [44]. In accordance with published human studies that report a beneficial effect of *P. freudenreichii* consumption on biomarkers of colon carcinogenesis [45, 46], we propose here to consider dairy propionibacteria in combination with TRAIL, taking into consideration that drugs have limited efficacy due to intrinsic or acquired chemoresistance and monotherapy using TRAIL has been shown to have no therapeutic effect in clinical trials [25]. Such beneficial bacteria may not only enhance tolerance towards treatments, but also enhanced its efficacy. Propionibacteria main metabolite, propionate, is a short chain fatty acid with described effects on histone acetylation, modulation of proliferation/apoptosis balance, and more recently beneficial effects in the context of metabolic and inflammatory disorders such as obesity, diabetes and inflammatory bowel diseases, through the activation of specific G-protein-coupled receptors and modification of transcription factors [47]. We have previously shown that consumption of *Propionibacterium freudenreichii* by humans increased the amount of this probiotics in feces but also increased the total short chain fatty acids to a concentration of 50 mM [48]. These propionibacterial metabolites produced *in vivo* in the gut [22, 49, 50] accordingly trigger apoptosis [3] or necrosis in acidic conditions [4] in CRC cells but also apoptosis in the context of DMH-induced carcinogenesis in rats [5]. We further showed in this work that, provided in fermented dairy product, they enhance the pro-apoptotic effect of TRAIL. This dairy product corresponds to the ultracentrifugated supernatant, (FMUF) *i.e.* the fermented milk aqueous phase, devoid of caseins and of bacteria, showing that the active compounds are secreted. This FMUF is used here to mimick the consumption of a Pf-fermented dairy product. It represents the aqueous phase of the dairy product released upon degradation within the stomach and the small intestine. The major secreted propionibacterial metabolites (propionate and acetate) and TRAIL both induce apoptosis, but via different pathways. TRAIL triggers the extrinsic death receptor pathway and acetate/propionate mixture acts on cancer cells mitochondria and triggers the intrinsic cell death cascade (caspase 9 activation, ROS production, mitochondrial membrane depolarization and cytochrome *c* release). Moreover, propionibacterial metabolites induce histone acetylation and modulation of gene expression. This may explain their synergistic pro-apoptotic effects. Combination of TRAIL with propionibacterial metabolites has a great advantage to target several proteins of the apoptotic pathway (TRAIL-R2/DR5, FLIP_L_ and XIAP), rather a single gene or protein, possibly making its anticancer effects more robust. On the other side, the mitochondrial pathway plays a predominant role in drug-induced apoptosis [51] as in SCFA-induced apoptosis [3], which could explain the absence of synergistic effect between several anticancer agents and SCFA.

This work opens new perspectives for the development of specific food products, fermented by selected pro-apoptotic strains of *P. freudenreichii*. Such dairy products, designed for CRC patients, could produce and deliver SFCA within the colon and thus enhance efficacy of CRC therapies, especially if based on extrinsic apoptotic pathway.

## MATERIALS AND METHODS

### Cell culture

The HT29 and HCT116 human colon adenocarcinoma cell lines were obtained from ATCC (American Type Culture Collection, Rockville, MD, USA). p53^+/+^ and p53^−/−^ HCT116 cells were a kind gift of Bert Vogelstein and Kenneth W. Kinzler (Johns Hopkins University, Baltimore, USA). Cells were cultured at 37°C under a humidified atmosphere of 5 % CO2 in DMEM medium (GlutaMAX^TM^, high glucose, Life Technologies, St Aubin, France) supplemented with 10 % heat inactivated-fetal calf serum (FCS) (PAN, Dominique Dutscher, Brumath, France). HIEC human healthy colon cells were a generous gift from Professor J.F. Beaulieu, University of Sherbrooke, Quebec, Canada. HIEC cells were cultured in OptiMEM-I (Life Technologies) supplemented with 5% FCS (Hyclone, Fisher Scientific, Illkirch, France), 0.01 M Hepes (Life Technologies) and 5 ng/ml EGF (Promega, Charbonnieres, France). Stable Bcl-2 transfected HT29 cells (HT29-Bcl2) and empty vector transfected HT29 cells (HT29-neo) were cultured as previously described [21].

### Chemicals and antibodies

Recombinant human Flag-tagged TRAIL was obtained from Alexis Biochemicals (Enzo Life Sciences, Villeurbanne, France). 5-Fluorouracil, cisplatin, etoposide, oxaliplatin, propidium iodide, methylene blue, menadione, acetate and propionate sodium salts, and caspase substrates Ac-DEVD-AMC (N-Acetyl-Asp-Glu-Val-Asp-7-amido-4-methylcoumarin), Ac-IETD-AMC (N-Acetyl-Ile-Glu-Thr-Asp-7-Amido-4-methylcoumarin) and Ac-LEHD-AFC (N-Acetyl-Leu-Glu-His-Asp-7-amido-4-trifluoromethylcoumarin) were obtained from Sigma-Aldrich (Lyon, France). RNAse A, PI (propidium iodide), Hoechst H33342, 3,3′-dihexyloxacarbocyanine iodide (DiOC6(3)) and dihydroethidium (DHE) were obtained from Invitrogen (Cergy-Pontoise, France). Annexin V-FITC (AV) kit and 7-aminoactinomycin-D (7-AAD) solution were provided by BD Biosciences (Pont-de-Claix, France). A pan-caspase inhibitor z-VAD-fmk, and z-FA-fmk were obtained from Calbiochem (VWR International, Fontenay-sous-Bois, France). Bradford reagent was obtained from Bio-Rad (Marnes-la-Coquette, France). Proteases inhibitors cocktail and PHOSstop were obtained from Roche (Roche Applied Science, Meylan, France). Antagonistic mouse monoclonal antibodies directed against extracellular domain of human TRAIL-R1/DR4 and TRAIL-R2/DR5 were from Alexis Biochemicals and were used for blocking apoptosis or for studying TRAIL-R1/TRAIL-R2 membrane expression by flow cytometry as previously described [52].

Antibodies used for immunoblotting were mouse antibodies to caspase-8 (clone 12F5, Alexis Biochemicals), cytochrome *c* (clone 7H8.2C12, BD Biosciences), Hsc70 (clone B6, Santa Cruz Biotechnology, Tebu-bio, Le Perray en Yvelines, France), p21 (sc-6246, Santa Cruz), PARP-1 (clone 7D3-6, BD Biosciences), FLIP (clone NF6, Alexis Biochemicals), caspase-3 (clone H-277, Santa Cruz) and caspase-9 (Cell Signalling Technology, Ozyme, Saint-Quentin-en-Yvelines, France), and rabbit antibodies to Bax (sc-493, Santa Cruz), Bak (G-23, Santa Cruz), Bcl-X_L_ (L-19, Santa Cruz), Mcl-1 (sc-819, Santa Cruz), XIAP (2042, Cell Signaling Technology) and Histone H3 (acetyl-Lys 9, ADI-905-705, Enzo Life Sciences). Polyvinylidene difluoride (PVDF) membranes, horseradish peroxidase (HRP) conjugated secondary antibodies and ECL plus kit were purchased from GE Healthcare (Saclay, France).

### Bacterial culture, supernatant and fermented milk preparation

The *Propionibacterium freudenreichii* ITG P9 strain (Institut Technique du Gruyère, Actilait, Rennes, France) used in this study was provided by the CIRM-BIA (BIA138, formerly labeled TL133, Centre International de Ressources Microbiennes - Bactéries d'Intérêt Alimentaire, INRA, Rennes, France). This strain was previously used *in vitro* and *in vivo* and triggered apoptosis [3, 5]. Preparation of supernatants in DMEM (SN) was previously described [3] as well as fermented milk (FM) or fermented milk ultrafiltrate (FMUF) [6]. Production of propionate and acetate was quantified in SN, FM or FMUF by HPLC as previously described [3].

### RNA extraction and transcriptomics (Microarray service, Miltenyi Biotec)

Two independent experiments performed in duplicate were done leading to 24 samples (4 NT, 4 C3/C2 (30/15 mM), 4 SN (1/2), 4 TRAIL (100 ng/ml), 4 TRAIL+C3/C2 and 4 TRAIL+SN). RNA was isolated using standard RNA extraction protocols (NucleoSpin^R^ RNA II). The quality of RNA was checked via the Agilent 2100 Bioanalyzer platform (gel and electropherograms). In addition to this visual control, the Agilent 2100 Bioanalyzer expert software allows the generation of an RNA Integrity Number (RIN) to check integrity and overall quality of total RNA samples. All RNA samples revealed RIN values between 9.7 and 10. Linear T7-based amplification of 100 ng of each total RNA was performed and to produce Cy3-labeled cRNA, the RNA samples were amplified and labeled using the Agilent Low Input Quick Amp Labeling Kit following the manufacturer's protocol. Yields of cRNA and the dye-incorporation rate were measured with the ND-1000 spectrophotometer (NanoDrop Technologies). The hybridization procedure was performed according to the Agilent 60-mer oligo microarray processing protocol using the Agilent Gene Expression Hybridization Kit. Briefly, 600 ng Cy3-labeled fragmented cRNA hybridization in hybridization buffer was hybridized overnight (17 hours, 65°C) to Agilent Whole Human Genome Oligo Microarrays 8×60K using Agilent's recommended hybridization chamber and oven. Fluorescence signals of the hybridized Agilent Microarrays were detected using Agilent's Microarray Scanner System. The Agilent Feature Extraction Software (FES) was used to read and process the microarray image files. The software determines feature intensities (including background subtraction), rejects outliers and calculates statistical confidences. For determination of differential gene expression FES derived output data files were further analyzed using the Rosetta Resolver^R^ gene expression data analysis system (Rosetta Biosoftware). The Resolver Software allowed the export of e gene list with all normalized sample/control-log10 ratios. Genes with fold change FC≤2 or FC≥2 and *P*<0.05 were considered differentially expressed. The microarray data were deposited in NCBI GEO data base (GSE67033).

### Cell death assays

Cell viability was assessed by a methylene blue colorimetric assay as previously described [6]. Microscopic detection of apoptosis or necrosis was carried out after nuclear chromatin staining with Hoechst 33342 and propidium iodide (PI) as previously described [53].

### Cell death analysis by Annexin V/7-AAD staining

After treatment, cells were stained with Annexin V-FITC and 7-AAD according to the manufacturer's instructions and fluorescence was analysed by flow cytometry [6].

### Cell cycle analysis

Cell cycle analysis was performed as previously described [6].

### Analysis of mitochondrial membrane potential (ΔΨm)

Disruption of ΔΨm was monitored by flow cytometry using 3,3′dihexyloxacarbocyanine iodide (DiOC6(3)) probe as previously described [6].

### Reactive oxygen species detection

Production of ROS was assessed by flow cytometry using dihydroethidium (DHE) probe to detect superoxide anion (O_2_^.−^) as previously described [6].

### Caspase activity assay

Activity of the caspases -3, -8 and -9 was assessed as previously described [21].

### Western blot analysis

Whole-cell and cytosolic fractions were prepared and immunoblotting analysis conducted as previously described [6].

### Statistical analysis

Student's *t*-test for unpaired samples was used to analyze data from cell viability, apoptosis, caspase activity, flow cytometry analysis of annexinV/AAD staining, mitochondrial membrane potential, cell cycle or TRAIL death receptors expression.

## SUPPLEMENTARY TABLES







## Abbreviations

TRAIL: TNF-Related Apoptosis Inducing Ligand
SCFA: short chain fatty acids
Pf: *Propionibacterium freudenreichii*
SN: supernatant
FM: fermented milk
FMUF: fermented milk ultrafiltrate
HIEC: human intestinal epithelial cells
HDAC: histone deacetylase
AV: annexin V
SD: standard deviation
