# Supplementary material for: The probiotic Propionibacterium freudenreichii as a new adjuvant for TRAIL-based therapy in colorectal cancer
Source: Oncotarget. 2016 Jan 11;7(6):7161–78. doi: 10.18632/oncotarget.6881 (PMC4872776; doi:10.18632/oncotarget.6881)
Supplement: Supplementary file 1 [file oncotarget-07-7161-s001.pdf]

SUPPLEMENTARY TABLES

Supplementary Table S1: List of genes overexpressed in cells treated with either TRAIL or C3/C2 or SN, or TRAIL+C3/C2 or TRAIL+SN.

See Supplementary File S1

Supplementary Table S2: KEGG pathway enrichment analysis of genes overexpressed in treated cells using FatiGO tool. List of genes are compared with genome and the significance of KEGG pathway enrichment was indicated as p-value. KEGG ID: KEGG Identifier. Hsa: homosapiens.

See Supplementary File S2
