# Supplementary material for: The probiotic Propionibacterium freudenreichii as a new adjuvant for TRAIL-based therapy in colorectal cancer
Source: Oncotarget. 2016 Jan 11;7(6):7161–78. doi: 10.18632/oncotarget.6881 (PMC4872776; doi:10.18632/oncotarget.6881)
Supplement: Supplementary file 2 [file oncotarget-07-7161-s002.pdf]

Supplementary Table 1 :

**TRAIL (n=314)**	**C3/C2 (n=2933)**	**SN (n=3408)**	**TRAIL + C3/C2 (n=33[illegible]**
ACPT	IL10RB	HDGFRP2	RPS6KA1
XLOC_l2_006021	MARCH3	CALML6	CAMK1D
LRRC1	FAM104A	SLC7A5P1	XLOC_008337
IRF1	SNORA12	LOC645638	CCRN4L
SNORA53	C13orf15	NR6A1	ABCD3
LOC115110	SCML1	LAMA5	WNT7B
XLOC_003471	GZF1	LDLRAD1	CNP
PRRT2	CCL27	ATP13A2	MLF1
NRP2	GLTPD1	MCTP2	LINC00240
C8orf39	LOC100653017	CLIC3	FAM110C
FOSB	ZNRD1-AS1	LPCAT2	SNORA42
SGPP2	ARL17A	SEC14L1	FBXO31
IKBKE	GIT2	PLA2G12A	FAM102B
IL4R	G0S2	SNORD83A	TBC1D22B
LOC100509780	INPP4A	ZNF30	NUB1
LOXL4	ZNF654	RGS14	CHD5
SNORD101	RAB11FIP3	TTC7B	MLLT4
STAT5A	WDR26	BBS12	VWF
PTGER1	AGSK1	SLC7A2	HAUS6
LOC149773	KIAA0895L	CDH17	SNX21
XLOC_002383	PACSIN2	MARCH3	AMIGO3
XLOC_002736	RECQL4	NPIP	TCEA3
IL27RA	SOCS4	SNORD36C	XLOC_l2_011265
MUC5AC	WRAP53	WNK2	SULT1A4
TAPBP	KCTD11	GDI1	LOC283174
LAMA3	SNORD25	LINS	CDK2
BACH1	NME7	SNORD48	XLOC_006153
PRO0611	CEP44	YIPF1	PRO2852
XLOC_003665	DOPEY1	TBC1D9	SERPINE2
PYGM	SPATA6	KIF17	SIGIRR
CMTM3	PCSK5	ELMOD3	TBC1D8B
IFNAR2	CCDC17	DNAJB11	KIAA0664L3
EXOC3L4	TSC1	CYB5R1	LOC100132247
XLOC_012229	C1orf9	LOC100132247	MGC16121
NPW	IQCD	BRPF1	STK32C
BATF3	PIAS1	PSPN	CXADRP3
XLOC_l2_008031	HES5	SH2D6	LOC100506514
CCDC17	GTF2B	PPP1R12B	RAB33A
LOC100128077	XLOC_002921	LPIN1	SLC22A23
LOC728228	P2RY6	FOXK2	MXI1
GJB4	AMBRA1	DFFA	C1QTNF5
IL15RA	MAPKBP1	VWA1	XLOC_011331
XLOC_006037	STK17A	SLC8A2	XLOC_l2_005076
DUSP5	XLOC_006138	MAP3K9	GLA

ZBTB46	LOC728431	RELL2	MICAL1
XLOC_l2_013734	XLOC_012586	TRIM29	FAM46A
ICOSLG	CBL	LOC100288902	NDOR1
XLOC_004924	RPRD1A	ESYT1	XLOC_008100
LCE1B	PLCE1	NEK10	SNORD30
LAMC2	PER2	NUPL1	GLIS3
C15orf63	LOC387895	TP53INP2	XLOC_006321
XLOC_009677	FAM65C	PDXK	XLOC_l2_005438
TAP1	WDR83	LOC100131089	MAFK
P51957	PELI1	ARPC4-TTLL3	CSGALNACT2
COL20A1	GNRH1	IVD	SEC24A
ALDOB	GPC1	CDC42BPB	MLL3
CXorf48	CAPN12	SUFU	C12orf52
C9orf128	PP12719	SLC38A10	TMEM41A
C6orf222	TERF2	FAM104A	RPS6KL1
IFNGR1	GIT1	USP37	SPTSSA
IGFBP1	TNFRSF10B	TGFBI	XLOC_007262
XLOC_000055	HCP5	CLMN	GNAI3
SYNGAP1	FAM107B	IL18	LEPREL2
TRIM31	ZNF697	IER3	BNIP3L
LOC100505940	RASSF10	VAT1	XLOC_011765
IL2RG	KIF3C	HLA-DOA	LOC284837
PRDM1	SYT15	SCARNA17	AMD1
ERVH-3	GGT1	DUSP13	B3GAT3
CD74	ARHGEF18	TSPAN13	SNORA33
XLOC_007214	INSIG1	ANXA1	XLOC_l2_006027
LOC349196	SNX25	ATG4B	XLOC_l2_010963
XLOC_014247	SLC25A30	LAMC2	XLOC_l2_007802
WNT10A	STARD3	LOC100128001	DMWD
KRT17	LOC284630	GGTLC2	LOC100130357
VTRNA1-2	ADIPOR2	C17orf69	XLOC_007813
XLOC_014418	SLCO1B1	XLOC_002577	SUSD2
PIGR	CGB	PGS1	SELK
AQP7P1	P39194	C15orf48	NAT14
DAPP1	HSPG2	ZNF124	UBXN7
ATPAF1-AS1	ZNF277	ZNF280C	XLOC_l2_001138
CBR3	RLTPR	SDF2	GGT1
CCDC108	TM2D3	AOC2	XLOC_011223
PDLIM4	MIPEPP3	UBOX5	NRP2
LOC553103	ANKRD13D	SLC30A1	LOC100289511
XLOC_000543	ZNF695	BCL3	ZNF441
FLT3LG	XLOC_012624	KATNB1	MGC23284
DIO3OS	FHL3	LIPH	ZNF284
IRAK2	TAOK2	DPYSL3	FLJ39051
LCE1C	ODF2	SYNJ2	XLOC_013350
KIRREL3	C9orf5	SMG1	MGAT4A
TNIP1	LAMB2	LOC100507018	SLC23A2
CDKN1A	ACOX1	C14orf43	RAB11FIP3

SPIB	XLOC_l2_007427	LOC100506305	PHRF1
PPP1R14D	PC	CLU	RCAN2
AQP10	PPP1R18	MLF1	FUCA1
TRIB2	PCDH1	TMEM120B	LPIN1
RND1	PPP1R12C	SEC16A	AKAP5
KRT6B	TTYH3	CEP68	AREG
AKAP4	CHAF1A	CASP9	PPP1R12B
CDRT1	DNAJC6	CIC	PGAP1
FLJ31104	LOC100506990	PELI2	TNFRSF13C
IGFL4	PLEKHO2	USP32P2	DOPEY1
SDR16C5	WFS1	CTU2	C1orf210
XLOC_l2_005933	HKDC1	LIPG	RASA4
XLOC_l2_013120	MAN1B1	XLOC_013914	XLOC_011521
LOC387895	XLOC_004122	DRAP1	CHN2
XLOC_l2_008595	SYNJ1	PER2	C1QTNF8
C12orf61	XLOC_l2_013730	SNX29	PANK3
RNU4ATAC	SMOX	FADS2	UBR2
XLOC_006080	POLR2J4	PVR	CEP112
IL3RA	PAIP2B	DENND4C	SSBP3
XLOC_003327	TRIM3	PTGS2	ZNF608
SERPINA3	DGKQ	C6orf228	INO80B
PSORS1C3	FANCM	CKM	FAM135A
AKT1S1	HSPA1A	POGZ	LAMA3
S100A3	GBA2	FBXO15	CIDEC
LOC100509620	AGT	PMP22	CYP2D6
RTP1	HIST3H2A	B4GALNT3	NFKBIB
SH2D1B	XLOC_008678	XPR1	SRGAP2
LOC642422	CBX7	RAP2B	LACTB
XLOC_004747	SYT13	POTED	QSOX1
XLOC_l2_015038	GYG2	XLOC_012530	CYP4F35P
SSC5D	LOC100652797	TRIP10	CREB3
MCCD1	XLOC_000111	PHGDH	MPRIP
NFKBIA	SYNGAP1	psiTPTE22	GNAI2
NEURL3	HES2	CA12	TSPYL2
SDC4	GRID2IP	SNORA12	FAM100B
PPP4R1L	PRINS	RBM4	LOC650368
SPRR1A	XLOC_l2_007928	CALU	OPN3
IL1B	GDPD5	LINC00483	TTYH3
BCL3	FBXO16	KIAA2013	CD109
XLOC_010031	PTPN1	EID2B	LOC400958
PDGFB	XLOC_009122	WRAP53	SNHG5
LOC284570	IQCE	LAMB1	FAM171A2
XLOC_010245	KIAA1432	SNORD89	GPER
UNC5C	PALLD	MAP7D1	ARHGEF18
MCAM	SPTSSA	PTPN1	TAF13
NAV3	ZNF555	RHOU	SNORA80B
RNF207	ID3	FAHD1	FAM132A
IL23A	STRA6	XLOC_008916	XLOC_012679

CFLAR-AS1	RAPH1	PREPL	C8orf47
TTTY5	SERPINA6	CD46	USP15
CPNE5	PRAF2	ELL	LOC100653016
LINGO1	PAOX	TTLL1	LOC100506714
TRIM42	INSIG2	GNB1L	CARHSP1
XLOC_007438	SPATA13	C9orf85	WDR26
PTGS2	WIPI2	STAT3	ARHGAP44
XLOC_001537	TBC1D10A	XLOC_l2_007644	LOC100129380
XLOC_009361	XLOC_001023	MYH9	MMP25
XLOC_l2_004706	PKD1	QSOX2	C2CD4A
CX3CL1	SNX29	LOC100507006	LOC100507487
ICAM2	FBRSL1	CRABP2	POFUT2
XLOC_009791	C5AR1	C12orf70	ZNF511
XLOC_005747	C19orf59	LOC283710	DYNLT1
CNRIP1	LOC100506821	PFKFB3	FKBPL
NFKBIE	XLOC_l2_015964	MAD2L2	ZNF77
XLOC_l2_005667	KIDINS220	KDM4B	LOC284072
TNFRSF6B	APBB3	TSC2	PLB1
SEMA7A	NINJ1	RNF149	SNORD83A
SPATA8	DNHD1	PLIN2	COL20A1
TNFAIP2	MAP3K3	INPP5A	NAV1
XLOC_009826	KIAA0226	DOCK6	XLOC_000670
XLOC_003093	TPTE	CCDC88B	HPN
KLK10	DRAM1	LRP1	NPRL2
ZNF331	LOC254100	PTPRJ	SLC22A1
TMEM31	LINC00299	ABCD1	C2CD4C
XLOC_000114	TBC1D8B	XLOC_001099	XLOC_012945
IL32	FAM167B	LOC100131829	C12orf44
LOC100505966	XLOC_011448	GALNS	ARHGEF19
LOC100288432	RIPK4	SLC2A6	MKL1
PP12613	CLCNKB	PIP4K2A	ZGPAT
ASB2	C1orf226	SORT1	FCAMR
XLOC_000842	SLC2A8	XLOC_013413	TPPP
DIO3	ZSWIM4	WDR66	MAP6D1
CACNA2D3	RAB40B	SOX12	LOC100507420
CD83	SEC24A	SNORD83B	SLC25A28
LOC100129119	DUSP8	UPK1A	ELMOD3
LOC100506178	DEFB1	C1orf81	C15orf39
SPAG17	RARA	MT1X	GATA2
NFKB2	FUZ	FRMD8	C6orf47
SNORD116-19	MGC23284	MALAT1	PMPCA
ITGAM	GAGE2B	C9orf140	ACOT7
MMP2	ASPG	XLOC_l2_015034	FAM167B
ICAM1	PDLIM7	NCOA3	BMP8B
XLOC_000545	XLOC_011223	HCP5	KIAA1804
XLOC_011559	GGT3P	GCAT	HLA-DOA
CXCL6	GAS2L2	MTHFD2L	MAX
GPR37L1	CITED2	XLOC_008781	C17orf76

LOC100616530	GSTT2B	CDC34	ZNF774
LOXHD1	C9orf89	ZNF295	XLOC_006037
PLAU	TBC1D8	QPCTL	GCH1
LOC646168	LOC100507336	PNMA1	XLOC_014243
LOC100505473	UBR4	LOC100499467	POU3F1
XLOC_l2_014821	OR13A1	NDNF	OR13A1
HOXD12	HAUS6	FAM135A	IL6ST
ZNF503-AS1	LOC100506714	EPB41L2	E2F2
OTOGL	XLOC_001856	SYNGR1	RFPL3-AS1
LOC728763	XLOC_006613	LOC100509213	XLOC_010238
XLOC_003736	VCL	APBB3	TMEM2
SH2D2A	LRRC69	GRINA	ATP2B4
HSD52	LCAT	NCF1	ACADSB
PKD1L3	UBE2Q2P3	NPL	XLOC_l2_004771
CTLA4	MPZL3	POU3F1	C1orf9
DNAH5	SH2D6	DNAH2	FANCM
LOC100128517	RASA4	SPTSSA	GRK5
C12orf12	TNFRSF13C	XLOC_001537	GGTLC2
LOC100505869	ECE1	LOC100507165	MAD2L2
FLJ43390	XLOC_l2_011204	XLOC_009122	CLDN9
LOC100128095	ELL	ATP6V0B	MALAT1
HCN4	ZNF774	XLOC_l2_004640	LOC100129399
XLOC_003965	ZNF821	XLOC_000043	HIST3H2A
XLOC_003791	MICALL2	SLC26A6	SLMAP
1/2-SBSRNA4	NUAK2	XLOC_000683	LOC729513
UNC5D	ARL6	CHN2	XLOC_l2_009501
XLOC_001128	C2orf44	ZHX3	PKD1
C2CD4B	DNAH6	GMCL1	NCK2
CXCL3	LOC283174	KIAA1609	SYT13
XLOC_002688	SNHG7	SHANK2	PAQR4
XLOC_013531	PIK3R3	NPM2	SGPP2
LOC399708	XLOC_009944	PP12719	SLC7A5
XLOC_003668	LINC00483	LOC100506848	KIAA0907
XLOC_006999	JMJD7-PLA2G4B	AKIRIN1	FLJ23867
LOC100130278	CYP2C18	C1orf170	ZNF709
LOC100506048	DOCK6	EFNA5	ANAPC2
ATP6V0D2	ZFP90	ZIC5	UCA1
XLOC_008625	C2CD4A	CROCC	HCAR3
RELB	LOC100506713	CMIP	FLVCR1-AS1
LY86-AS1	MIA3	PPFIA3	CD86
XLOC_l2_009180	SDC4	SLC25A29	PCYT2
XLOC_006456	CNN3	FXR2	ATG4B
ART1	NOXA1	SERPINA4	NOXA1
MPP4	Q8WNA4	HRASLS2	LRRC69
LOC400499	DTX2	GYLTL1B	SCML1
CSF2	MPRIP	PPP1R13L	NQO2
FLJ42842	CARD14	FLJ39051	XLOC_l2_006745
PI3	SEPP1	PAQR4	POTEE

STOML3	LOC100507580	LOC100505675	CHD4
LDLRAD2	NOSIP	MARCH9	HYAL3
XLOC_000856	BAK1	CSNK1E	FLJ32224
XLOC_l2_008190	PLB1	DBNDD1	PHKG2
CXCL10	LOC100506848	RTBDN	DENND5A
TNFAIP3	PSPN	DUSP14	LTBP3
POM121L8P	DAAM1	MAX	XLOC_006138
XLOC_000824	MOB2	N4BP1	PQLC1
TMPRSS11B	SUN1	EXOC3L4	ING2
XLOC_011881	NQO2	CDH1	DFFA
XLOC_006485	TRIM62	LOC728431	PPFIA1
FLJ33534	MAD2L2	FAM106CP	CYP26A1
C3orf55	XLOC_013072	DPM2	TAF3
CXCL2	FAM132A	C3orf71	TMEM120B
XLOC_l2_003888	RBM38	GGN	EPB41L5
XLOC_011326	TUBGCP6	GGCX	RIMKLA
XLOC_011614	SEC14L1	S100A14	STX7
TAS2R16	CASKIN2	FAM83F	RPS6KB1
XLOC_011289	FLCN	OR2AG1	LOC100509213
LOC100506571	FLJ90757	NBR2	DNHD1
LOC441204	CAV1	PI4KA	CEP68
CRB1	SPSB3	C3orf18	BICD2
XLOC_006398	AKAP5	LOC254100	GMCL1
XLOC_009713	XLOC_006994	CD82	SH3D21
TMEM132B	GNL1	MACF1	MYADM
LOC440970	RPL28	SEPP1	LOC100132495
XLOC_011378	XLOC_013732	PML	ETS1
XLOC_000324	TMEM92	XLOC_001441	ZNF503
PRKG1	AMN1	XLOC_000340	LOC100506312
XLOC_l2_008450	SGK223	C5orf45	RNF213
XLOC_l2_010258	FNBP1	SAMD4B	ZNF124
XLOC_009661	RHOU	C6orf25	SNRNP48
CABP2	SNORD22	GALNT2	XLOC_014388
XLOC_008366	SEC1	LOC100506123	HEBP1
XLOC_007093	SLC22A1	ST14	HHEX
BIRC3	XLOC_002185	INSIG1	ND1
MYO18B	PPP1R9B	XLOC_011805	SPDYE5
ADCYAP1	CDKL5	TCFL5	CT62
FAM205B	DNAJC12	KIAA0895L	AKIRIN1
Q6TXI9	MKLN1	OCLN	OTUD3
TMEM229B	VWF	CXorf40B	C21orf56
LOC157381	BAI1	AAMP	PLAU
XLOC_003636	AIF1L	SPN	SYNE1
ECSCR	GSTT2	FANCE	STRA6
XLOC_011421	FRMD8	DHX16	GLTPD1
SLITRK2	NARF	ANKRD11	MFAP2
XLOC_l2_006138	TCP10	NDOR1	C10orf118
XLOC_003754	PECAM1	FBXW10	RNF19B

LOC286114	ZNF519	C20orf96	PECAM1
XLOC_l2_014217	CRY1	FBXO31	GZF1
XLOC_001067	LILRB3	INPP5F	AFAP1
XLOC_007545	GYLTL1B	XLOC_l2_009571	LRRC31
XLOC_009546	GRIN2C	CHP2	SPOCK2
XLOC_001676	MUC1	NR3C2	RHOBTB3
SHROOM2	MALAT1	SNX27	XLOC_005273
XLOC_007916	DCUN1D2	NSAP11	CEP170
XLOC_000955	ATP7A	HYAL3	BDNF
OR14A16	XLOC_001748	ZRANB1	C9orf85
XLOC_011350	RGS2	SLC38A2	XLOC_002133
FGF14	FLVCR1	CEP19	RAP2B
CXCL1	TMED6	FGFRL1	AFF4
LOC100507096	C7orf60	DRD4	TRIM25
XLOC_001483	SLC38A2	UCN	GAS2L2
C10orf120	KRT23	SOCS4	KRTAP3-1
XLOC_l2_013149	FAM131B	LOC100130745	PDE4D
CILP	GNAI3	SNAPC1	SLC5A3
IL17C	MYO1E	PPM1K	SAMD4A
ICAM4	SNORA68	IL1RAP	TCIRG1
XLOC_007727	LAMB2P1	NOXA1	RAB17
XLOC_006620	KLHL28	TBX10	TRIM26
PRDM16	XLOC_002987	HSPA5	MRGPRF
CFHR1	SLC31A1	SNHG7	TMEM136
IL8	XLOC_001537	C2orf81	C16orf86
IL4I1	MKL1	AP2A1	CACNG8
LTB	CNTNAP2	PAQR3	FLJ43663
TNF	LOC643650	TBC1D22B	NPAS2
CCL20	FBXO15	PAQR5	SYNGAP1
UBD	CXADR	HSD17B3	ARMC2
	LOC440934	PRKD2	AIF1L
	CPLX1	AGFG2	LOC100287728
	ZNF79	CDH24	NUP43
	LOC100507800	SLC25A28	NOTCH1
	SNX27	GPT	APBB3
	RGN	SERPINA6	DAND5
	SARDH	LOC100131820	RLF
	CIDEC	ADHFE1	PCBP4
	TESK1	HCFC2	CGRRF1
	HSPC072	CCDC69	KIAA0895
	ZNF324B	PRO0628	NARF
	RTN4R	SH3D21	P51957
	LEPREL2	ETV5	LOC100506990
	FOS	GOLGA6L9	CCDC85B
	TNFSF15	RASSF6	CYP2C18
	SLC7A2	XLOC_005566	MOSPD3
	SERPINF1	SYTL4	KIAA0319
	NPC1	DNAJB13	STAMBP

	LIPA	XLOC_l2_004371	XLOC_l2_015038
	RHOBTB2	NQO2	XLOC_006291
	TMEM41B	MXRA7	FLJ21369
	PTPRE	MCFD2	RAB43
	XLOC_004590	CXADRP3	AQP7P1
	SLC7A5	BAIAP2L1	ENPP1
	XLOC_000683	ISG20	NNAT
	AAK1	LAT2	KIAA1549
	RCAN2	EPHB3	AAK1
	L1CAM	XLOC_013350	LOC100505904
	RECK	FAM25A	PMM1
	XLOC_009943	RAB43	SMG1
	ZNF778	CCDC75	GGCX
	TBC1D16	HK1	SNORA80
	psiTPTE22	PHF20L1	FAM27A
	C17orf51	BNIPL	TSPAN32
	ZNF584	ABHD3	LOC100652766
	HPSE	GRIK1-AS1	GAN
	XLOC_005361	KIAA1671	XLOC_008353
	KIAA1539	CCBL1	CCND3
	ZBTB8OS	ZNF10	SYNGR1
	ZFAND5	GGT1	CREB3L2
	XLOC_012162	JDP2	CPLX1
	DNAJC5	SRPK2	SH3BP2
	GOLGA6L10	SCAP	C21orf91
	FGFR1OP2	XLOC_003480	CPSF3L
	SNHG12	CREB3L2	ACSS2
	XLOC_004827	IRAK2	XLOC_005244
	SPATA2L	HIPK1	LOC100506245
	ADRBK2	GBA2	SNORD35A
	ADRA2C	TOP1	XLOC_006513
	KCNQ1DN	RBBP5	XLOC_l2_010330
	XLOC_000922	LOC650368	OAS1
	ZNF516	SPANXB2	YBX2
	PSORS1C2	S100P	KCNK1
	PGAP1	ZNF75A	TMEM189
	SPDYE5	ZNF3	KLC2
	KIAA0247	C19orf59	COL11A2
	LOC100288092	HIVEP2	TMEM231
	RXFP4	ANKRD27	MIDN
	CDC42EP2	GPR116	CNTNAP1
	XLOC_l2_011265	BCR	TBPL1
	LPCAT3	FOXD4	TP53INP1
	OTUD3	F11R	MCTP2
	NFIL3	AKT1S1	SYT15
	NCKAP5L	MRPL43	RND3
	SAMD8	PLEKHG6	AGAP3
	EGLN3	STK24	TLR1

	XLOC_l2_013931	TTC28-AS1	EFHC1
	CLCN4	SNORD25	TMEM139
	RTBDN	CHMP1B	SLC16A13
	LOC283710	USP12	FLRT3
	LOC100507165	STX5	LOC100652915
	TNKS1BP1	STAMBP	FAM161B
	TNFSF12	CHTF18	PIK3CD
	BTN2A3P	SLC2A8	XLOC_l2_009571
	ERP27	ATG16L2	ADRBK2
	C3orf35	LOC100505634	LOC100509256
	ACHE	UBXN7	LOC100131831
	MXD1	XLOC_010161	TRIM3
	PDXDC2P	HINFP	SLC6A8
	TAF3	LOC100653030	TPMT
	PHLDB3	PRKCZ	HCLS1
	C1orf52	ACBD7	ULK4
	LOC338817	XLOC_l2_011901	COQ10A
	CCDC64B	IFNAR2	ANKRD36B
	XLOC_003787	MOK	LOC100507395
	DNAJC28	LRWD1	XLOC_006588
	HERC1	ARHGAP4	BCL2L11
	TMEM184B	CLDN12	ETV3
	JPX	FGFR1OP2	CDH24
	WDR91	PLXND1	KCNK6
	NEAT1	XLOC_013932	RMRP
	EPB41L5	NEDD4L	DKFZp434J0226
	XLOC_007775	ZCCHC14	STK4
	XLOC_l2_005714	FBXL14	XLOC_l2_013485
	CHN2	C1orf52	CCNL1
	GDPD1	TOM1L2	C19orf77
	MYL9	ITGA3	MFSD2A
	LOC100509263	PCBP4	PTK2B
	XLOC_l2_001064	MAPKAPK2	PPTC7
	IZUMO4	DGKD	ZNF584
	LOC729668	FANCB	ZNF3
	AMY1C	WNT7A	ATP7A
	PTAFR	SLC12A9	ANO3
	PPP1R12B	CDH16	ADRA2C
	OBSCN	ATP2A1	LOC100506157
	WTIP	XLOC_008619	LMTK2
	ABCA7	PIP5K1B	PDK4
	FGFR3	FLJ35024	SAMD8
	MARCH9	FKBP5	HRASLS2
	MORN3	XLOC_004122	CRAT
	FKBP7	TESK1	Q6NT14
	CSF3	SEC14L2	LOC284581
	NPAS1	CAB39	C12orf51
	OSBPL7	BAHD1	ZNF251

	GGCX	RNF183	ECE1
	PLIN4	ZNF833P	CHP2
	RHOBTB3	NANOS3	PPL
	OVGP1	STON2	ZFPM1
	MLF1	TBC1D10B	SLC16A10
	MAP6D1	GPR137B	DNAJB1
	GJB4	MANBA	MEGF6
	LOC100505904	DNAH7	MXD1
	LOC100289092	SLC22A1	PPARD
	TTC21A	NUDT17	SLC12A4
	ARHGAP29	LRP6	TMEM104
	XLOC_l2_000010	ANO6	LAMB2
	ACOXL	PLXNA2	JPX
	LSS	TMEM8B	PRIC285
	LOC286071	HS6ST1	TMEM229B
	KLHL7	LRRC1	XLOC_005442
	TMEM190	C1orf228	SIRT7
	LOC100131820	SLC22A5	PDPK1
	HLF	HAUS6	OVOL1
	PGPEP1	SNORA52	CEACAM18
	XLOC_l2_007528	MYO7A	LOC100289079
	CHTF18	UBR2	XLOC_l2_004854
	XLOC_012139	XLOC_009788	EPHA2
	GALK1	AGSK1	MGC16703
	SLC6A6	IL18BP	FLCN
	SNX22	PLEKHM1	SNX25
	KISS1	XLOC_012754	DEFB1
	CCDC88B	KLHL30	KATNB1
	GMCL1	SIK1	SNORA64
	LOC645638	C19orf26	MAP7D1
	KIAA0319	KCNC4	C15orf5
	ZNF284	PLAG1	SCXA
	TBPL1	AHR	RUNDC3A
	MAFB	PAQR8	LOC100507580
	PMM1	PRKACA	LY6G6C
	LOC286272	ARL13B	TLE3
	FLRT3	REN	CBL
	C19orf28	ARRB2	XLOC_l2_001134
	XLOC_011016	VKORC1	SNX27
	VAT1	NAALADL1	TESK1
	XLOC_009006	CD14	FLJ90757
	SBF1	PSAT1	VTRNA2-1
	LOC100289388	MAP6D1	AOC2
	MYO15B	ZNF554	DENND4C
	N4BP3	GZF1	PTPRVP
	MIA2	ZFAND5	UBE2Q2P3
	KLC2	TTC18	VWA1
	GRIN1	PCSK5	B3GNT4

	INSR	SLC35E4	C12orf70
	CEP76	MOB2	XLOC_013994
	SYCE2	SERPINC1	TIPARP
	CCND3	DNAJC12	LOC100131234
	LRRC8E	NPC1L1	PDE4C
	LOC100287728	PTCD3	OGFR
	PEX13	LOC400499	ANXA6
	ODF3B	ABL2	XLOC_010352
	SZT2	FLJ43681	CGN
	XLOC_l2_007783	XLOC_000350	RBM38
	XLOC_002705	DAB2	RDH13
	CDK5	PCSK7	PRR5-ARHGAP8
	SYNE1	EDA	USP49
	CISH	XLOC_011223	XLOC_l2_013293
	XLOC_014243	TPRA1	LOC554206
	XLOC_001230	BTRC	LCA5L
	BCL2L11	STXBP4	LOC440300
	FLNA	SNORA65	HCFC2
	XLOC_010190	CYP2D6	SLC6A6
	CCDC74B	C1orf55	MIA3
	CCNE1	SGMS2	IQSEC1
	LOC388210	LOXL3	HSPG2
	CYP4F35P	C10orf137	ANKRD36
	MXRA7	AMBRA1	LOC440900
	LOC553103	TNFRSF13C	KIAA0247
	GTPBP3	DNAJC5	LOC100506073
	MMP15	BREA2	TBC1D16
	XLOC_005228	AZGP1P1	GPC1
	MAST3	TEX2	TMEM184B
	OXTR	LOC100505933	PLCE1
	CDKN2B	ART5	LOC388210
	XLOC_l2_013293	RALGAPA2	SERAC1
	SRRM3	HES5	XLOC_009681
	GPBAR1	FAM108A1	KRTAP5-4
	SLC22A23	C4orf19	BBS12
	XLOC_008679	GLI4	TBC1D24
	SNX30	SDCBP2	MAPKAPK2
	C3orf25	LOC100506001	WDR19
	VEGFA	RLF	ZFAND3
	LOC100131089	ATP7A	C17orf28
	FBXO3	DYNC1H1	ADAM10
	LOC100289090	LOC100507309	XLOC_l2_005714
	XLOC_l2_013001	AAK1	VPS18
	LOC100130171	CD74	XLOC_009191
	XLOC_013923	CKB	C3orf71
	DOK3	DNAL1	LOC100509263
	XLOC_l2_013873	ARHGAP29	KHK
	MED26	ARHGAP44	FGD6

	PLK3	ADRBK2	SCARNA5
	XLOC_l2_012415	RAB26	XLOC_001072
	SPIRE1	STK32C	EXOC3L1
	XLOC_000671	XLOC_013072	XLOC_005228
	PDPK1	RNF181	IGFL1
	CCL3	SEMA6A	DUSP7
	NUB1	LOC100127886	CEP72
	TTC18	SLC6A8	TSC22D4
	SPTBN5	COL16A1	XLOC_000527
	LOC100506245	RAB33A	SNORA68
	LOC100129399	SBK1	XLOC_l2_007928
	PTHLH	GTPBP2	XLOC_008556
	FAM131A	MAPK8	LOC730101
	SYVN1	MKL1	DOCK6
	ART5	XLOC_005810	PEX13
	PIGH	ATP2B4	FAM131B
	TSEN54	CRISPLD2	DEPDC7
	CHAC1	XLOC_009602	PCNXL2
	H1FX	LOC202025	HSPA1A
	TPPP	DEPDC7	CRTC2
	AFMID	LOC100506990	LIX1L
	IFNAR2	ZBTB5	KLHL15
	IGSF3	TIE1	IL36G
	PLA2G7	ZNF598	CSF1R
	RELT	LMF2	RPS20
	LOC652990	SH3BP2	FAM131A
	IDI2-AS1	RAPH1	FBXL20
	XLOC_009681	P4HA2	KIF21B
	XLOC_006513	ZMIZ1	LOC642852
	MIR7-3HG	STX1A	FADS3
	CCDC157	SPATA2L	MARCH9
	TPTE2P3	ADAM20	NELF
	POU3F3	ZDHHC8	ARHGEF26
	RAB43	GPBAR1	C15orf58
	TTLL13	HSF2	DGKZ
	ABL2	WDR67	FAM132B
	ATG4B	CTSL2	FAM66D
	SYTL4	NUB1	SSSCA1
	FCHSD1	PCNXL2	UGCG
	FADS3	ACBD4	GYLTL1B
	ATP2A1	CYP1A2	TSPAN9
	SBK1	XLOC_006505	TMEM81
	C6orf124	DTX2	XLOC_000595
	PBXIP1	TTLL7	FBRSL1
	SLC26A1	SPATA6	LOC100132057
	VILL	ABCA2	ZFAND2A
	AIM1L	TPST2	PRSS30P
	CYB5R1	COL13A1	XLOC_005185

	PYGM	SPTBN5	C5orf27
	FBXO33	CPT1A	SLC2A8
	TMEM136	ATP6V0D1	RALGDS
	XLOC_l2_001138	GGT3P	SNORD74
	GRIN2D	FBXL20	SERTAD2
	XLOC_010591	SMAP2	ISG15
	PPM1K	STK40	SNORD89
	TMEM200B	PAPLN	STK11
	C20orf106	RUNDC3B	PML
	SELPLG	SLC31A1	MGC50722
	COL6A1	MICAL3	CCNL2
	ADAM20	DLX4	PDE5A
	LUZP1	ATL1	CDK18
	ARHGAP44	SNHG1	POTED
	TRIM6	CDC14B	TNIP1
	CIC	SNORD84	TRA2B
	PAFAH1B2	TSPAN32	XLOC_010244
	CCNL2	SECISBP2L	TTC21A
	TMEM104	HLA-DMB	XLOC_011618
	SNORD76	ZDHHC14	STARD3
	XLOC_000214	USP20	AHR
	PGP	ZNF211	SLC25A22
	CORO7	LOC100506800	ZNFX1
	ADAMTS10	LPCAT3	PCDH1
	TRAFD1	LOC100287813	BCAR4
	FAM118A	NKD2	SEMA4D
	XLOC_009147	GLTPD1	XLOC_l2_004611
	SBF1P1	C6orf124	TBC1D9
	FAM171A2	HSPG2	C1orf52
	PDE9A	XLOC_013950	PER2
	ADAM10	RPS6KA5	APH1B
	GGT7	FAM91A1	KRT14
	TNNI3	ZNF436	DUOXA1
	ADORA2B	XLOC_l2_005179	PPP1R9B
	SLC19A2	XLOC_006335	HIST2H4B
	TBC1D24	PHLDB3	C1orf195
	C16orf86	TBC1D16	ZNF516
	GPER	LOC100129781	MCOLN1
	XLOC_000194	MGAT4A	FLJ35390
	PPP2R3B-AS1	ATP13A3	CTH
	LOC100289187	WNT4	BAI1
	ANKRD54	COX19	SLC25A29
	VANGL2	RNF41	TSC2
	LOC554206	DNAJB1	XLOC_l2_013001
	CSGALNACT2	TMEM154	SNX29
	C19orf77	STIM1	LOC646626
	PDHA1	LOC643837	DEDD2
	DENND4C	XLOC_011563	ND5

	TNXB	MAP3K10	ILF2
	DAB2	GK	PPM1K
	GRIN3B	XLOC_011924	XLOC_l2_009316
	CCP110	TM2D2	DCUN1D2
	ATP8A1	ULK4	ULBP3
	TP53AIP1	IL6ST	YPEL5
	MYO10	C13orf16	AKT1S1
	RHPN1	RAP1GAP	RLTPR
	LOC146513	MIDN	KLHL30
	SPN	NPIPL2	SYT7
	NEXN-AS1	SNORA59B	SERPINA6
	SLC2A6	RPL28	DNAJC5
	FAM108B1	EPS15L1	LOC388152
	TSPAN13	TUBAL3	ZNF555
	COL20A1	CLCN4	SPATA13
	C17orf59	NRSN2	ANKRD54
	TP53INP1	PPFIA1	FGFR3
	PRKAB2	KEL	MAP3K3
	LMF2	PPAP2A	TNFRSF1B
	SLC9A3R1	LOC554206	EMP3
	CHRM4	CRY2	PPP1R12C
	NCF1	LOC100129034	GSTM4
	XLOC_l2_015213	XLOC_007776	HSPA1B
	NDOR1	TSPO2	HDGFRP2
	CDA	TMEM59L	WDR37
	ACER2	LOC440132	SLC46A1
	PDE4C	RHOBTB2	AQP7
	LOC100652915	SPTAN1	LOC100130419
	WWC3	ATP7B	ARRB2
	GGTLC2	DYNLT1	ANKRD43
	CBFA2T2	IPO9	LCN15
	ZNF878	GALK1	STK17B
	BIVM	ATG4A	FOXO1
	SLC5A3	RIMKLA	SLC19A2
	ZNF3	ACSL1	C9orf89
	SLC22A2	TNFRSF4	CHD9
	RNASE4	LOC729652	EZR
	C17orf69	C17orf28	FAM108A1
	IL6ST	LOC100506605	HOTAIRM1
	XLOC_013950	MYO10	LOC100127886
	XLOC_014088	FBXO3	IGSF3
	XLOC_011025	ENDOV	MAP2K3
	TCEA3	JARID2	PPP2R3B-AS1
	PIKFYVE	CCND3	XLOC_009944
	ATP7B	AFF4	GPR3
	MZB1	LOC100653017	C5AR1
	MARK4	PRKAB2	SLC22A2
	LOC100130899	MYO9B	NMUR2

	OCR1	FEM1C	LTB4R
	EFHC1	KLHL28	MBNL2
	IFFO2	CHD9	IRF2BP1
	INPP5F	USP49	XLOC_008357
	ATG16L2	CCNE1	LOC100653017
	PAPLN	TRIM10	AASS
	ABHD6	ARMC2	KCNAB2
	C9orf96	HN1	LINC00260
	RHPN2	KCTD5	ABL2
	LOC100132057	MATN3	SRPK3
	ZFYVE1	TAGLN	FCHSD1
	FAM89B	ANKRD13C	IRF6
	XLOC_002133	EZR	BRI3
	LOC100268168	TBC1D2B	LRWD1
	LMF1	SNORD99	ATP2A1
	FBXO31	ENPP4	LOC100507347
	E2F2	CLDN7	LOC100507918
	PRSS8	PKD1	MBOAT7
	EML6	DCUN1D2	PHACTR3
	ANO8	KIAA1432	RHPN2
	LOC100507445	LOC401561	FRMD8
	MAPK15	SGK1	CDHR5
	GOLGA6L9	BTN2A3P	STX1A
	XLOC_002113	NEK1	MYT1
	XLOC_007262	LOC100505633	RAB37
	XLOC_003872	GSTT2	LOC100507165
	ZBTB48	PALLD	ITPK1-AS1
	LOC100505669	VPS13C	PPM1B
	LOC100507373	SDHAP1	ATG4A
	LOC100506859	MOCS1	PDXDC2P
	XLOC_005341	TMEM185A	ITGB7
	C5orf45	THBS1	RECQL4
	CXXC1	C12orf44	KATNAL1
	HOXA1	ZNF608	ANO9
	PDE5A	RNFT2	SLC6A10P
	WNT7B	XLOC_l2_010831	ANKRD23
	RSPH3	CCDC85C	ZNF433
	C10orf116	MR1	FLNC
	CTSF	LOC100505882	C17orf69
	Q6P4E4	GNA12	SPN
	KCNJ18	UBXN11	ZNF519
	C17orf56	PTPRVP	MYPOP
	XLOC_003881	ZNF780B	PPAP2A
	LOC100130015	HIP1R	RAB3B
	IDS	LOC729041	MYO7A
	SIRT2	XLOC_l2_010330	XLOC_005900
	LOC100128869	HOXB13	FXYD7
	XLOC_004165	LOC643650	KIAA1432

	ZNF608	CACNA1I	TMEM170B
	PTCH1	CDK18	SNX30
	GPR162	LOC100132240	CEP44
	FAM69A	GTF2B	CDHR3
	SLC41A1	GSTT2B	C14orf45
	KCNC4	C1R	LOC283588
	PLCB2	PFKP	CCDC87
	ATP6V0A1	FANK1	MOK
	ENPP1	GAN	GCNT1
	DNALI1	IL28RA	CLDN11
	PHYHIP	PBXIP1	ZNF878
	LOC100289580	IFFO2	LOC100130876
	SH2D1B	XLOC_003738	XLOC_001023
	LOC100128477	IFI30	SEC31B
	TBX3	CCDC64B	RN7SK
	NOS1AP	TULP3	NINJ1
	PQLC1	MIA3	LOC100271831
	ZNF709	UBXN10	RHOD
	AVIL	NOTCH1	XLOC_011480
	LOC100505933	AFMID	C16orf93
	SCARNA20	ACSL3	KDELC1
	ZDHHC8	XLOC_012688	PAPLN
	CEP68	XLOC_000490	SLC41A1
	ECI2	TMEM92	DTX2
	LOC440910	C16orf80	NRARP
	ANKRD27	STXBP1	PIK3R3
	ATP8B2	SIRPG	XLOC_012162
	LOC100507347	DYNC2H1	SPAG9
	FAM160A1	VWF	PDLIM5
	YBX2	RHCG	XLOC_012053
	LOC497257	ZNF778	XLOC_001507
	DYNLT1	PDE4C	JAG1
	TMEM132A	XLOC_l2_000010	PIAS1
	TNNC2	C1orf138	FAM91A1
	TMEM98	LOC100507594	CDC14A
	LOC100134259	LRRC16B	RNF114
	LOC389199	XLOC_002035	AGPAT4
	C17orf28	FAM160A1	XLOC_l2_010511
	MYLK2	PLEKHA4	RDX
	ASRGL1	XLOC_003386	FAM107B
	LOC283588	HPN	GSTT2
	CGRRF1	LEPRE1	GOLGA8IP
	GLIS3	RLTPR	PRKCA
	TMEM81	ACVR1C	ZFAND5
	DPY19L2P3	FLJ37644	TAGLN
	C15orf60	PRRG1	PLEKHG6
	ELF3	KHDRBS3	SPIRE1
	PNPLA7	PRKAG2	XLOC_011766

	GRK5	F10	LRRC39
	XLOC_l2_014098	XLOC_014243	CCNE1
	LOC388152	SNAPC2	GPT
	XLOC_l2_010831	PDXDC2P	INSR
	WDR19	LINC00341	DNMT3B
	PPP1R15B	PDGFB	PLEKHG2
	MAPK8IP3	sept-12	WDR91
	LOC100507395	ENPP2	ZFYVE1
	GAS2L1	FMNL2	PDE6D
	XLOC_l2_010330	LUZP1	MARCH3
	XLOC_012281	NOSIP	SLC25A27
	SLC9A6	GALNT6	MAPKBP1
	NANOS3	XLOC_l2_006745	XLOC_003349
	FRY-AS1	SNX25	ZCCHC14
	YPEL5	SLC2A1	SNAPC2
	LOC100499221	IQSEC1	TRPM5
	TPMT	XLOC_009181	SBNO1
	LOC100506866	GPATCH1	SNX22
	SSC5D	KIF16B	TMED6
	CYFIP2	PECAM1	RAB44
	MYBPC2	ZNF709	LOC100505669
	KIF16B	ISG15	SEC14L1
	IQSEC1	XLOC_012162	XLOC_l2_007834
	CTSL2	METRN	ACER2
	PLEKHM1	NPAS2	CRABP2
	LOC728061	OCEL1	LOC100505933
	LOC392288	XLOC_l2_008151	sept-12
	PNPLA2	XLOC_013301	UBR4
	VNN2	SLC22A23	MED26
	ULK4	PRR5-ARHGAP8	LRRC16B
	KIAA0930	PMPCA	WWTR1
	POR	AFAP1-AS1	XLOC_004122
	LRRC39	HOXC10	MPV17L2
	XLOC_004366	PGPEP1	XLOC_l2_008203
	C16orf87	XLOC_003482	CAPN12
	NOTCH2NL	CBL	C17orf51
	ACADSB	CERS5	SHOX2
	TBC1D2	CLEC16A	ZNF821
	XLOC_l2_015127	PC	ZBTB8OS
	PTPRVP	C16orf87	CIC
	STK31	ICOSLG	NFIL3
	PDE4DIP	PDE9A	SNORD22
	ANPEP	CDA	LOC100128563
	ZNF251	TMEM145	NR4A2
	CFP	TSEN54	GRIN3B
	SYS1	STK4	TRIP10
	FLJ26086	FRRS1	C19orf28
	OPN3	CLCN5	EID2B

	LOC100506890	LYG1	CYP39A1
	KLHL15	XLOC_013445	GGT7
	ANGPTL2	QSOX1	PLIN2
	MKNK2	SYT15	NPAS1
	ANKRD13B	CYP39A1	ELL
	XLOC_004430	HLA-F	LOC100128001
	METRNL	PPARGC1B	ZNF211
	LOC100507278	GNA11	HPSE
	ANKRD23	LOC729668	SERPINA5
	SCG5	CNIH2	SNORD31
	SLC23A2	NT5DC3	TTLL5
	PORCN	OTUB2	PP12719
	LINC00482	RAB9A	UBE2Q2P1
	XLOC_010286	POFUT2	LOC100509638
	ATG4A	CRELD1	CHST7
	ADAM8	LRRN4CL	C7orf54
	XLOC_011256	MAD1L1	PDE4DIP
	DKFZp451A211	DGKZ	RNGTT
	ARHGEF26	WDR19	C5orf56
	FLJ42022	EXOC8	ADORA2B
	TNK2	TNFSF12	NPHP3
	MUC12	C1QTNF5	POLR2F
	SERAC1	SLC2A2	SNORA10
	BMP8B	LOC642852	LOC100506553
	HIST1H2AC	AK1	AHRR
	RAB9A	SCARNA20	KCNJ13
	KCNK5	FAM102B	XLOC_013942
	CABYR	AQP7P1	CAMTA1
	IGFALS	CD6	XLOC_011448
	CD52	SEMA4F	LOC92659
	XLOC_009576	AFF1	ZNF778
	PPIL6	GPT2	CTSL2
	C17orf76-AS1	STRN	LOC100130899
	LOC100653030	P2RY1	PBXIP1
	LOC100506605	MAFF	XLOC_009576
	ADM	NRARP	SMAD7
	ERO1LB	P39194	HIP1R
	TSGA10	ZNF251	LOC729668
	LPAR5	TMEM104	LOC100294362
	SCD5	G0S2	SNORA65
	WNT10B	TPMT	DAAM1
	LOC100616668	SLC25A36	RPL28
	MAPK8IP1	XLOC_l2_000399	GPR137C
	XLOC_l2_006944	PPP1R18	KCTD11
	TMEM170B	SNORD76	ZNF174
	C2CD4C	SAG	C14orf43
	NEIL1	PAFAH1B2	ZNF598
	FAM69B	LOC100128198	GSTT2B

	MAPK11	TGFBR3	VILL
	TSC22D2	LCN15	SPDYE2
	DDR2	SHB	C10orf47
	SGCA	HLA-DOB	LOC729159
	IZUMO2	XLOC_009261	RAB30
	LOC100128001	LCA5L	CCL27
	CEP19	SERPINA3	C20orf106
	PFKFB2	PTCH1	Q6P4E4
	XLOC_012592	EPHB6	SNORD84
	TTC28-AS1	CCDC112	XLOC_001441
	C9orf24	RSU1	GGN
	LOC728802	LOC100130899	FBXO3
	DNAJB13	PGAP1	XLOC_l2_005020
	FLJ42392	PDE6D	PLEKHA4
	CTH	SNORA44	XLOC_l2_010724
	ULK1	ZNF79	LOC100190939
	PLAG1	WWTR1	CNTD2
	DAGLB	NEBL	RAB26
	MAP4K2	PMM1	XLOC_013445
	XLOC_009724	RUNDC3A	CKB
	XLOC_l2_009050	PCYT2	SELPLG
	CHST7	CDX2	KLHL28
	HEBP1	TMEM189	LOC440905
	UBAP1L	SPECC1	CNNM2
	HHEX	SNORA64	LOC100127885
	LOC100499467	C17orf51	PHLPP2
	NDRG1	SYNGAP1	C9orf140
	XLOC_007504	XLOC_l2_011204	MMD
	LHX1	XLOC_l2_005076	GAK
	HPN	ARL6	C17orf109
	PLIN3	SLC6A10P	XLOC_011837
	IL17RA	TLE1	LOC390940
	STAMBP	CNP	COL16A1
	PTGS1	GOLGA2	ENPP5
	DIRAS3	MBD5	PRRG2
	LOC100128670	SLC1A5	CCDC19
	NAB2	XLOC_l2_007802	SEC16A
	ZNF556	GAK	XLOC_003738
	LOC100130357	MAP2K3	NAMPT
	XLOC_l2_008151	ZNF217	LOC100268168
	CKM	LOC283174	XLOC_012754
	TANC2	XLOC_000670	GTF2B
	ACSS2	ABLIM1	XLOC_l2_011901
	EMP1	EFCAB3	MUC12
	LOC100129924	UNC5A	CCDC82
	XLOC_l2_010511	SPSB3	PPP1R32
	TSPAN9	XLOC_013364	RAPGEFL1
	PPM1N	PGBD1	SRGAP2P2

	LOC100131829	SPAG9	EXD3
	FAM161B	CLCN2	KIAA1671
	C1orf228	MAML2	XLOC_005361
	XLOC_011104	XLOC_003881	CCDC85C
	RPL32P3	ANKRD13B	PLEKHO1
	LOC100132077	TBC1D8	SNORD83B
	CEP72	LEPREL2	SLC25A35
	MAP1LC3B	WDR91	NDRG1
	SNCAIP	MORN3	SMTN
	HCLS1	MYL9	LOC100291105
	MPV17L2	PDPK1	LENEP
	RFPL4A	DNAJC16	TMEM190
	MCOLN1	TPPP	SLC12A7
	SPOCK2	SNORD31	AMY1C
	LOC642852	C20orf54	FAM160A1
	C16orf55	MXD1	XLOC_002921
	C17orf109	JMJD7-PLA2G4B	MYO1E
	MAP2	XLOC_l2_013001	AMH
	KRT80	RHPN1	CCDC157
	C9orf72	ZNF584	PPARGC1B
	LOC100507487	OTUD3	TRIM4
	LCK	NOTCH2	RGS9BP
	ACBD4	RTP3	XLOC_009498
	XLOC_l2_008259	PCSK4	SLC28A2
	POU2F2	MED26	ZNF385A
	KLRG1	LOC100506183	LOC652990
	XLOC_001048	BCL2L11	C1S
	MBOAT7	CBX6	MOB2
	XLOC_005748	RAB7L1	Q93YZ4
	CREB3L2	LOC100128019	AGSK1
	C20orf96	TTLL10	LOC100128477
	LOC284108	TBC1D10A	XLOC_011025
	LACTB	TSPAN5	HS6ST1
	XLOC_003734	FAM69B	LAMA5
	RPUSD1	PDE4DIP	GAPVD1
	KIAA1549	IL10RB	PAOX
	NGDN	NME7	C1orf201
	REEP6	SEL1L	ZDHHC11
	EFCAB3	SLC19A2	PDK2
	STX1A	PTPRE	SEMA6C
	C16orf93	SNAPC4	HDAC10
	GOLGA6L6	HMHA1	C1orf124
	LOC100129973	DBN1	FZD5
	ALAS1	CDHR5	ZNF79
	KSR2	FGF11	PLCG2
	PIDD	SLC25A35	AFF1
	TRIM52	RUNX1	PFKFB2
	IL28RA	CSF2	LUZP1

	KSR1	SCARA3	XLOC_000546
	NFKBIA	SBF1	FLJ22763
	DEPDC7	LOC100190939	RNU1-5
	B3GNT4	LOC100507233	CENPT
	KCNAB2	BCAM	TOB1
	AKNA	RHOD	DBNDD1
	CDHR3	ZNF654	GNA11
	TMEM120B	PLEC	C4orf38
	ZNF503-AS1	C5orf27	LOC100132167
	PLSCR2	RPPH1	LOC100652730
	TTC16	KIF25	GGT3P
	CMTM1	PPP1R9B	FBXO48
	IGLL1	ANKRD54	PHLDA3
	C1orf201	SSC5D	TTBK2
	NAT14	KIRREL3	PTPN13
	GIPR	YIF1B	IFFO2
	UTP23	FLJ32224	PCYOX1L
	OCEL1	PAOX	RSPH3
	DNAL1	DNASE1L2	MYL9
	ZNF324	TMEM184B	DBN1
	SLC30A2	KANK2	CNOT4
	CLMN	LOC729732	XLOC_012586
	GFOD1	GYG2	LLGL2
	GPR153	LOC100132057	XLOC_010881
	CLIC3	COQ10A	FBXO43
	SAT2	SLC25A44	PRDM2
	LOC100506633	TSNAXIP1	RAB9A
	C16orf70	ZNF516	HOXB13
	XLOC_l2_004640	C9orf89	ZNF697
	XCL1	XLOC_l2_008221	ACOXL
	HBEGF	PIK3CD	SPTB
	GSTM1	CLIP3	XLOC_006505
	MGC50722	XLOC_011765	ZDHHC8
	XLOC_006505	SNORA53	XLOC_008995
	FGF11	RORC	LOC100289187
	RTTN	ZFAND2A	XLOC_l2_006152
	UBE2Q2P2	PADI2	C19orf26
	FBXL20	LOC100127885	SBF1P1
	CDX1	SEC31B	PDLIM7
	FARP2	IQCH	XLOC_013950
	AZI1	GNAI2	PGRMC2
	NAV1	COL20A1	LOC100128071
	DBNDD1	CAMK1D	LOC497257
	LOC91450	CHRNA10	FAHD1
	DNMT3B	CYP2B6	JUNB
	RUNDC3A	NXF1	HVCN1
	XLOC_l2_011901	LOC100128477	VTRNA1-3
	XLOC_001576	KLC2	XLOC_003872

	ZNF177	NAMPT	C17orf59
	PURG	REC8	INPP5F
	RAB24	FGFR3	ND2
	SPON1	EPHA2	FNBP1
	TTBK2	ELL2	PGPEP1
	DNMT3L	ZNF821	XLOC_009016
	FOXD4	ECI2	ADARB1
	PPFIA3	XLOC_002356	TSEN54
	Q93YZ4	ZBTB47	TTC25
	LCN15	LOC440300	PVR
	ASB6	LRIG1	MYH3
	ANO3	LOC100129363	KIF25
	CYP2B6	C8orf46	LOC100652777
	ROM1	IZUMO4	CABP1
	XLOC_011918	OSTM1	SNORD12C
	XLOC_012754	HERC1	XLOC_l2_006021
	LOC100505832	ZFPM1	SLC9A3R1
	TTC25	S100A1	XLOC_003734
	XLOC_000388	GRIN3B	SCARNA4
	ELMOD3	KCNAB2	SEC14L4
	GGTLC1	XLOC_l2_003897	OVGP1
	TMEM139	MYPOP	WDR83
	PRKCA	CCDC157	XLOC_l2_000706
	FAM108A1	XLOC_003235	BTN2A3P
	CCDC82	LOC100128402	C17orf56
	XLOC_000527	HK2	CHTF18
	MMD	FAM107B	LOC728431
	RASGEF1B	PDIA2	FYN
	APLP1	EIF4A3	XLOC_l2_007644
	TSL	LEAP2	POR
	SPSB1	FAM132B	BACE1
	GPRIN1	MAPK8IP2	FBXO36
	C15orf62	GTPBP3	GAST
	EXOC8	MBNL2	ACCN3
	FAM91A1	RAB11FIP3	DAGLB
	SERPINA5	XLOC_002069	ITLN2
	PLIN2	WDR26	PSPN
	GAK	XLOC_l2_011798	CCP110
	FUCA1	ROPN1L	UPP1
	LOC440905	ARFGAP1	TUBGCP6
	ESAM	FRY	XLOC_006319
	LRRC31	ACER2	XLOC_l2_013873
	SNORA61	DDX26B	LOC100130581
	TM2D2	C11orf45	CLMN
	LOC100128857	CX3CL1	LOC100505930
	ACBD7	LOC100507800	XLOC_l2_015127
	CHRD	CD81	ADCY10
	SCARNA8	EPB41L5	RPS2

	KIAA0913	FAM18B2-CDRT4	SLC26A1
	XLOC_l2_006152	KIAA0226	TBC1D2
	RELL2	WNT11	SNORD76
	MAPRE2	INSR	ACBD4
	CCDC85C	TRIM41	RNU2-2
	ATP8B3	PDZD7	MXRA7
	TMEM145	DKFZp434J0226	VNN2
	XLOC_l2_015209	TMEM190	IQCD
	TUBB2A	DISP1	XLOC_l2_014098
	LOC100506688	CALCOCO1	XLOC_000388
	RPL3L	RNF157	ANKRD42
	SLC9A3	KIAA1539	RELL2
	SMAD5-AS1	CDX1	LOC100128670
	RALGPS1	LOC100506219	TBC1D10B
	HIP1R	XLOC_l2_003674	PIM2
	KIAA0895	XLOC_001023	NFAM1
	GGN	SSBP3	ULK1
	FZD5	ANGPTL2	XLOC_l2_002204
	XLOC_008357	ZNF433	GRHL1
	ANKRD43	CA4	EGR1
	LOC100130547	XLOC_010500	DIO3
	XYLT1	XLOC_012515	LOC100505760
	BBOX1	RNF208	WNT4
	LOC100132167	KCTD13	SNORA17
	P2RY1	FLJ30064	PNPLA7
	DOC2GP	TBC1D2	CYFIP2
	TLR1	POLR2J4	FLJ16779
	CES1	INPP4A	LOC100653030
	C9orf139	FAM118A	BCAM
	TMEM189	LCK	FARP2
	RNF114	C3orf35	GOLGA6L10
	FAM90A1	PLCXD2	TAOK2
	RAPGEFL1	LOC100505668	XLOC_l2_009883
	CLDN6	LOC100506890	SCPEP1
	GPR116	CDC42EP3	TMEM145
	ZNF707	RHOBTB3	SNORA57
	XLOC_009183	C1orf9	LOC100131820
	LOC100505683	SIX1	KCNC4
	KCNC3	XLOC_005968	TMEM59L
	XLOC_l2_011043	NEAT1	SPACA4
	TEKT5	ASPG	PCSK5
	FBXO32	CEP170	SPSB3
	KHK	ZNF474	UBXN11
	RAB37	XLOC_002793	RAET1K
	LOC100507651	MPV17L2	FLNA
	XLOC_004229	TNFRSF21	PPFIA3
	POLR2F	ZNF555	C6orf124
	LEPRE1	PPTC7	JAKMIP3

	EXD3	NT5DC4	AFMID
	ERV18-1	C2CD4C	BAK1
	PGRMC2	FLCN	XLOC_003787
	VWA3A	SCML1	SIRT2
	SLC46A1	DEF6	CBFA2T2
	ATP2B4	ACOT7	TTLL10
	LGSN	GAS2L1	HLA-DMB
	XLOC_011480	RDH13	LMF2
	LIPE	SRRM5	NEURL1B
	KATNAL1	STK17A	UBAP1L
	FAM126A	SLCO4A1	MAN1A1
	SLC4A9	XLOC_l2_013931	ALAD
	MFSD2A	XLOC_011766	SNORD25
	PLCL1	LOC100131702	ENGASE
	C6orf132	OR4C46	ATF3
	MERTK	SPDYE2	PMEPA1
	CCDC126	XLOC_013866	GPR157
	JARID2	SPATA13	ZNF385C
	FYN	GOLGA6L10	XLOC_l2_007097
	MRGPRF	ACSS2	LOC100132741
	SPAG9	XLOC_l2_013293	CLCNKA
	HCFC2	IRAK1BP1	NPHP1
	SH3PXD2A	ABLIM3	KCNK5
	MYT1	C16orf93	P39194
	COLQ	TCIRG1	C3orf45
	ACCN3	LOC100652769	TTLL13
	SAMD4A	FZD2	WWC3
	UBE2H	SPTBN1	SMOX
	MOK	XLOC_005341	MLLT11
	AEBP1	SNRNP48	XLOC_009181
	AGPAT4	CCP110	IGLL1
	GSTM4	TBX3	CHRD
	CD3G	CGRRF1	USH1G
	XLOC_005968	KIAA0247	TMEM92
	HPX	STK17B	MAPK8IP1
	LOC100505551	ITLN2	LBH
	BMP2	XLOC_l2_011043	ACBD7
	NXF1	MIB2	MIR22HG
	PLXNA4	XLOC_l2_007783	PRODH
	NKIRAS1	ABCA7	FLJ37644
	SLC25A44	XLOC_001262	BREA2
	CERKL	AGPAT2	PAG1
	XLOC_013981	FADS3	XLOC_004834
	MLLT11	GPR162	NGDN
	ZNF221	C4orf22	SBF1
	XLOC_l2_014421	C21orf88	LRRC8E
	LIX1L	XLOC_014212	MAST3
	XLOC_011758	PARM1	VAT1

	SNRNP48	C1orf135	KCNJ10
	SYNGR1	MPRIP	SLC30A2
	CYHR1	EHD4	DNAH6
	ZNF385C	MAPK11	CBX6
	ERVW-1	HERPUD1	HN1
	SYT7	FAM171A2	SLC25A30
	ITPRIP	KRTAP5-8	SHB
	TRIM4	PQLC1	XLOC_014237
	FAM59B	XLOC_013506	TLE4
	MOCS1	SNX18	LIN7B
	SLC9A5	CD55	KIAA1467
	KIF5A	KIAA0930	GRIN2C
	SLC5A11	GRAMD2	PIKFYVE
	SEC61A2	CDC14C	ZNF890P
	AASS	RTTN	PAFAH1B2
	FGF8	F3	XLOC_l2_012415
	XLOC_l2_004317	CT45A1	ATP8B3
	EFNA4	LOC390940	SNORD75
	MIB2	GRIN2C	MEGF9
	C11orf94	TTYH3	SERHL2
	PHF1	XLOC_l2_012871	SYCE2
	ROPN1L	MKNK2	ZNRD1-AS1
	CCDC116	LOC100506428	P2RY1
	LOC283070	RALGPS1	MAP1LC3B
	CSRNP1	CBFA2T2	MPZL3
	LOC100128198	FGFBP1	CD274
	TCEAL5	ABCC1	DRAM1
	SHF	LOC100505725	XLOC_013958
	XLOC_001428	C9orf116	PI4KAP2
	XLOC_011563	ARSK	XLOC_011430
	HOXB13	ADIPOR2	TCFL5
	ARHGAP39	LOC440792	AK1
	XLOC_l2_003897	PTPRCAP	LOC100130285
	COL8A2	XLOC_005273	C16orf55
	CBX6	UBAP1L	XLOC_l2_013853
	HMOX1	CIDECP	ARHGAP29
	LOC152286	PROC	PLCXD2
	KLHL5	CEP72	MAFF
	NR6A1	MICAL2	AMBRA1
	XLOC_l2_005695	KCNC3	PGP
	XLOC_l2_014820	HPCAL1	PPP1R13B
	CSF1R	FAHD2A	XLOC_009457
	SAT1	LOC440910	HTT-AS1
	KLK8	SLC12A4	ZFYVE28
	WFDC1	MYO15B	RUNDC3B
	LOC340508	XLOC_l2_003974	C2
	DDX26B	LOC100505988	psiTPTE22
	XLOC_l2_003295	TRIM62	LYG1

	ALDOC	C17orf76	TTLL11
	PLA2G10	LOC202181	CXXC1
	PIP5K1C	PAGE2	EXOC8
	AHR	JUN	XLOC_000055
	TAS1R3	ENGASE	PLAG1
	KLHL26	FAM69A	PRSS8
	SCARA3	REEP6	SEC61A2
	TBX19	TAOK2	LINC00483
	LYG1	ILF2	TRAFD1
	FASLG	MAP4K2	XLOC_l2_007427
	SLC39A8	BCL6	XLOC_012192
	NFKBIE	XLOC_000048	LOC100131551
	GRM1	MAN1B1	RNF157
	C1QL1	LOC100268168	RAPH1
	LEKR1	SHD	MMP15
	KRTAP5-4	SNX22	ERN1
	ACAP3	PKDREJ	C9orf24
	KLKP1	SLC27A1	CCDC75
	C3P1	EID3	NKPD1
	STXBP4	KLK8	SNORD100
	ALS2CR12	SEPX1	LOC100506913
	HS6ST1	ACSF2	XLOC_003729
	DNAJB5	KIAA1984	TBX19
	LOC283887	EGR1	LCK
	METRN	RPS6KL1	RAB7L1
	XLOC_l2_009883	FAM132A	F3
	ZNF211	DNAJC6	LDLRAD1
	BBS12	ACOXL	DUSP8
	LOC100507401	WFS1	C9orf116
	HIC2	WNT7B	NCKAP5L
	PRODH	LRRC17	GALK1
	LOC100132474	XLOC_002741	RPL3L
	SLC16A3	XLOC_l2_005020	XLOC_008223
	NEK10	XLOC_002987	CCDC17
	ACSF2	TMEM229A	NR6A1
	TMEM59L	LOC100507580	ATMIN
	ZNF385A	SUN1	MECOM
	C10orf54	ANPEP	LOC728802
	XLOC_005991	OAS1	FGF11
	XLOC_007433	PIDD	PPP1R18
	C7orf61	XLOC_008556	PLA2G10
	FLYWCH2	XLOC_l2_013873	XLOC_l2_003974
	PI4K2A	CMTM1	HSD11B1L
	PLCXD1	LOC100509256	LOC100509620
	CYP39A1	ZNF77	FANK1
	SEMA6A	XLOC_013732	XLOC_008781
	MAN1A1	TESK2	GJB4
	HSD11B1L	KCNF1	PORCN

	TNNT2	NAV1	PREX1
	ISYNA1	ZNF296	ARFGAP1
	XLOC_005039	GPR137C	SLC28A1
	C9orf116	RNU11	SGK223
	DPF3	SCIN	MYO10
	XLOC_006933	GPAT2	ATG16L2
	PPP1R13B	XLOC_006726	PYGM
	GPR137C	XLOC_006321	ACSF2
	C7orf43	KIF7	HKDC1
	COL21A1	SLC3A2	RNU12
	LOC100507420	RASGEF1B	PRINS
	XLOC_l2_006745	C6orf132	SOX8
	XLOC_011350	ANKRD2	SNAPC4
	BACE1	WDR60	XLOC_l2_008888
	FBXO43	TSPAN33	CYHR1
	PVR	LOC652990	VTRNA1-1
	TRIM36	LOC100128857	CGB
	ABCD1	SLC9A3R1	EGLN3
	SLC25A35	POLR2F	MICALL2
	LOC100130157	AKAP5	LOC100130547
	NXPH3	LOC283624	FLJ30064
	ALAD	SPON2	XLOC_013506
	LOC100507563	ZNF221	SUN1
	AGPAT4-IT1	GKAP1	FAM65C
	LOC100131796	PLAT	REEP6
	LOC100131551	SBF1P1	LOC283050
	C1orf55	MAST3	FAM166A
	OTUB2	AVIL	ALAS1
	CCDC84	TRIM3	LINC00482
	PDE6D	ATXN1L	XLOC_l2_015209
	LOXL3	CASQ1	COL6A1
	GPR111	CCDC84	C16orf70
	ASMT	TNXB	EFCAB3
	LOC728228	PRKCE	MKLN1
	LOC100652777	GGTLC1	LOC100134138
	IGSF8	AQP11	PDHA1
	TNFAIP3	MAPK8IP1	TMEM110
	TCEAL6	ETS1	TBC1D17
	LINC00310	CDC42BPG	MID2
	ID4	DAAM1	CELSR2
	WDR60	C16orf55	RBSG2
	ZNF433	KLRG2	SNORA53
	ZFYVE28	ZSWIM4	NIPAL3
	CCDC87	UBE2Q2P1	FUZ
	SHD	DPF3	CT45A1
	OASL	ACCN3	TRIM10
	FRY	MAPT	PTGS1
	EPPK1	NIPAL3	SLC7A5P1

	LOC100506120	ARHGAP39	PLXNA4
	ST6GALNAC4	KHK	XLOC_001134
	LOC440900	GOLT1A	SBSN
	PIGR	PPIEL	PSORS1C3
	CAMTA1	DAGLB	XLOC_005087
	PRDM2	DIP2C	NOSIP
	CILP	LOC100130276	DNAJC12
	EID2B	LOC400084	DOK3
	ORAOV1	CTH	ELL2
	PPARD	MB21D2	FAM3B
	C4orf32	UNC5CL	TP53AIP1
	STK17B	XLOC_001134	PI3
	EHD1	ERI1	AK7
	GDF11	RANBP6	KIAA0930
	CX3CR1	CHRD	XLOC_011872
	XLOC_009868	SERP2	XLOC_000683
	XLOC_001738	MAP2	XLOC_l2_015033
	PLIN1	PGM2L1	C1orf226
	GABRE	MIAT	LOC338758
	TOR1B	IGFALS	GSTM1
	USHBP1	FAM167B	LOC100499467
	DBN1	DNAH6	GAFA3
	XLOC_000755	ARHGEF18	ATG9B
	ELL2	LOC100130849	XLOC_014139
	USP35	ZMIZ2	C15orf48
	PPAP2A	SAMD8	ING1
	FLJ22763	XLOC_004827	SLC38A2
	GRHL1	PDHA1	AIM1L
	LOC401052	TMEM45B	XLOC_001788
	SAG	XLOC_l2_001064	ODF3B
	PTK2B	FLJ23867	RTBDN
	TPRN	TRAM2	PPP1R15B
	XLOC_009261	MAFB	C10orf54
	LCA5L	XLOC_014105	CLSTN3
	DAPK3	XLOC_002383	TBC1D10A
	LOC440792	BAI1	XLOC_000794
	SLC24A6	SERTAD1	SCARNA20
	ANKRD29	CATSPERG	FLJ16124
	NGEF	XLOC_l2_015127	ATP13A2
	XLOC_004046	DEGS1	ZNF221
	TCFL5	ALS2	DENND2C
	RPS6KA2	SGPP1	FOXD4
	PPP1R14C	ADAM11	IFNAR2
	FAM85A	ISYNA1	MSMB
	XLOC_l2_010602	APBB2	C21orf88
	XLOC_005810	XLOC_l2_014098	XLOC_l2_010831
	RHOB	TNFRSF11A	PHLDB3
	TNFRSF4	GK3P	GPR153

	ZDHHC11	KIDINS220	GPRIN1
	XLOC_011413	MYLK2	XLOC_008614
	CEP112	S1PR4	LEPRE1
	XLOC_007908	C3orf45	CCDC126
	IRF7	TBC1D20	XLOC_005968
	ENGASE	FAM82A2	SERTAD1
	C14orf55	TNNT2	SCIN
	RAB7L1	WNT10B	TMEM38A
	LOC100506268	TTC7A	KIAA0913
	LOC100652769	LOC100129380	LOC645195
	LOC100129781	MARK4	KIAA1539
	LOC100505760	ULK1	TM2D2
	THPO	MYT1	LOC344595
	ADAT3	XLOC_009944	ADA
	FOXD1	CHST7	FZD2
	CIB2	SNX30	C6orf132
	XLOC_013788	MAPK8IP3	GABBR1
	USP17	LOC143188	XLOC_l2_008151
	SEMA3G	XLOC_006994	SLC9A5
	TMEM38A	TOR1AIP2	HOXA1
	XLOC_l2_014579	C7orf53	RGS14
	XLOC_014219	FUCA1	KLHL26
	ADAMTSL4	EFNA4	XLOC_001537
	XLOC_l2_013116	NCKAP5L	KISS1
	KRTAP5-9	FOXJ1	CIB2
	RBP7	KRT16	USP35
	TNFRSF25	RNF223	CRYGS
	WNT1	BACE1	RIPK4
	RHOF	HEBP1	MARK4
	XLOC_010601	XLOC_013788	SLC46A3
	MICA	NRP2	ANKRD29
	AHRR	NR2E3	TSC22D2
	XLOC_012568	C8orf47	NAB2
	MFAP2	MLLT4	NEK10
	XLOC_014096	TMEM81	SAT2
	DDIT4	ADARB1	OCEL1
	CELSR2	ROPN1	XLOC_004606
	XLOC_004282	ALAD	ANKRD27
	CYP26A1	TMEM132A	NGEF
	DMPK	NFE2	JARID2
	FAM49A	C10orf116	PAX7
	CBR3	SPACA4	C7orf43
	EFNB2	LOC100505904	C4orf47
	COQ10A	CXorf41	WISP2
	LOC440132	KIFC2	IRGQ
	TP53INP2	XLOC_001048	KLK5
	ZNF461	ADORA2B	MAPRE2
	NIPAL3	OSBPL7	XLOC_l2_012054

	DGAT1	SLC41A1	CDA
	PPARA	PAG1	HES2
	ADSSL1	GPR157	TCEAL6
	MAK	NKPD1	SLC30A4
	FAM90A7	NAB2	HLA-A
	FAM73B	LOC100130930	GRHL3
	TNF	PPP1R32	LOC100507588
	PDXP	GIT1	PIP5K1C
	MAGI2	FLJ30403	XLOC_l2_004640
	NEXN	FBXL18	LOC100129675
	TMEM110	ATP2C2	OSBP2
	XLOC_007205	LOC389791	LOC100129781
	FAM53C	P2RX5	DNM1P46
	ITLN2	LOC100130417	XLOC_l2_007371
	HECW2	C16orf70	TGIF2
	RSL1D1	AGPAT9	XLOC_003522
	CDC42BPG	UBE2Q2P3	FHL3
	POU2F3	FAM59B	FBXO32
	LOC400084	XLOC_007368	KIF3A
	XLOC_013932	CLDN6	TP53INP2
	C16orf5	RIOK3	C22orf23
	LOC100652768	HSPB8	CYP3A5
	GATS	C19orf28	CLIC3
	XLOC_013549	RBP5	MKNK2
	LOC339524	ADAM32	TMEM132A
	SEMA4D	IRF6	CMTM1
	PAGE2B	VMO1	GIT1
	XLOC_013866	XLOC_l2_013267	TNFSF15
	PRKCD	XLOC_008711	HBA2
	Q9RWL9	TMEM136	MYOM1
	ZFP2	XLOC_003474	C16orf87
	RAB8B	EFHC1	SNHG12
	FLJ30403	SOCS7	SCARNA8
	FAM154B	RNF39	SLC24A3
	LOC90246	RSL1D1	CD177
	XLOC_009457	ENPP1	TBX3
	TSPAN4	GPC1	SERP2
	RAB11FIP1	sept-04	ATP1A3
	XLOC_005488	IL6R	MAPK15
	FBXO36	LIN37	SLC35E4
	XLOC_000055	LOC440905	PDGFRL
	SGK1	PCOLCE	G0S2
	GCDH	PPIL6	EFNA4
	XLOC_001099	CCDC42B	SMAD5-AS1
	KCNN4	C10orf47	ADAMTSL4
	GCNT1	LOC285141	RXFP4
	SNORA14A	SNORA17	STARD9
	XLOC_001916	XLOC_014399	CCDC42B

	UNC119	LOC284440	ICOSLG
	LMBR1L	MUC17	XLOC_007775
	PLAU	LOC388152	IL28RA
	FAM182B	XLOC_009526	THBS1
	SPON2	LOC729040	ULBP1
	GNAS-AS1	ANKRD13D	H1FX
	KDELC1	B3GAT3	SNHG7
	TTLL11	CIDEC	ZBTB48
	XLOC_008100	FGD6	XLOC_011016
	AVPI1	FAM182B	ISYNA1
	TUFT1	LOC143666	ANKRD13D
	GABBR1	ZNF177	GAS2L1
	RAB26	LLGL2	LOC100132077
	DGCR14	PACSIN2	KIDINS220
	RNF122	LIX1L	WRAP53
	ZDHHC20	ABHD6	CCDC144A
	XLOC_008223	SCG5	C8orf46
	LOC100128402	WFDC10B	INSIG2
	LOC338653	TNNI3	EFR3B
	SEC14L2	LOC100132474	DDR2
	SERHL2	XLOC_l2_006152	CARD14
	RPL23AP32	RTN4R	APLP1
	XLOC_l2_013267	PIP5K1C	RELL1
	CT45A1	Q6BWL2	SH2D6
	LOC100506047	FBXO36	GOLGA6L9
	XLOC_l2_011873	IGSF3	HLA-F
	KIAA1462	XLOC_l2_001138	FAM126A
	SPIRE2	TP53INP1	TNFRSF10B
	SOX8	VTRNA1-2	SHF
	MID2	TOR1B	SYS1
	LOC100128071	ATP8B3	KCNN4
	MSMB	ETV3	ZNF324
	XLOC_000508	HEXIM1	NLRP12
	RGS9BP	GATS	PIDD
	XLOC_013100	FSD1	CDC42EP2
	RTKN2	FLJ43315	GPBAR1
	XLOC_010114	MAN1A1	XLOC_l2_015034
	NAGS	UBE2Q2P2	SPATA2L
	NPHS1	XLOC_l2_005695	HSP90AB4P
	RNF157	LONRF2	ABCD1
	INPP5K	GLIS3	DNAL1
	XLOC_001338	RNF114	SEMA3G
	GLCE	PLK3	ENDOU
	Q8N7V6	HIST2H2BF	DZANK1
	XLOC_014137	CHST12	PDE9A
	WDR78	XLOC_012139	KCNMB4
	LOC100133190	RIPK4	XLOC_001230
	SEC14L4	ITGA10	C2orf48

	LOC100507118	ASB6	PTAFR
	STRC	PLEKHG5	GADD45A
	ADA	FBXO16	SHD
	GJB7	FLNB	XLOC_013981
	ATXN1L	LOC619207	TNNI3
	KCNMB4	MICALL2	C6orf154
	SLC16A10	NBEA	UBXN10
	FAHD1	POU3F3	AVIL
	ARHGAP26	ZBTB48	CCDC64B
	C6orf154	ST5	XLOC_013866
	GGT8P	IDI2-AS1	PPP1R14C
	ARHGAP4	TTBK2	SPTBN4
	CXCL6	LOC100507364	AKNA
	PPARGC1A	ATP6V0A1	PNPLA2
	GRHL3	ZNF703	MERTK
	XLOC_014237	ENDOD1	XLOC_009868
	MBP	SNORA43	GDPD5
	FAM18B2-CDRT4	EFNB3	ANPEP
	LOC100652995	CCDC126	DNAJB5
	PCSK4	ELF3	SNORD43
	XLOC_011287	FAM194A	TNKS1BP1
	MAP3K14	XLOC_013955	PLEKHH2
	LINC00163	TNKS1BP1	ZNF707
	SPTB	ZFYVE28	ZNF703
	CYTH4	ST6GALNAC3	TSPAN4
	C22orf23	XLOC_l2_010494	XLOC_012048
	LOC441268	LOC153577	GUCA1B
	CLGN	PLIN3	ARHGAP39
	HMHA1	H19	SLC25A44
	XLOC_l2_007644	SCARNA8	OCR1
	SMPD1	PRODH	GLCE
	SLC16A12	IGLL1	RASSF10
	KCNN2	TRIM25	XLOC_l2_006944
	NEDD9	TCEAL6	LOC100289211
	XLOC_006319	SLC25A30	SERPINE1
	RFPL3-AS1	ADRA2C	UNC5B
	ARNTL	PYGM	XLOC_007857
	TMEM229B	AQP10	UBE2Q2P2
	RGS9	RAB24	PLEKHO2
	NPHP1	KIAA0895	ARSK
	ASGR1	XLOC_005087	NTF4
	C18orf8	LHX4	LOC100652797
	UNC5B	AZI1	WHSC2
	MICB	SRPK3	NKX3-2
	ARSK	HIC2	SEC14L2
	TUBB8	PHF1	GFOD1
	ARMC2	LAMA3	WNT1
	RAB3B	TMEM110	RNF207

	CBX4	SAT1	KSR1
	XLOC_003327	FAM89B	ROM1
	XLOC_013602	LOC283553	ADAM8
	SYNDIG1L	RPUSD1	DNM3
	MEF2C	XLOC_005082	MIAT
	KIF3A	SERTAD2	PLEKHM1
	COX6B2	LEMD1	MPP2
	XLOC_005963	LTB4R	C10orf116
	CEBPB	FLJ42022	XLOC_l2_014421
	SKI	RILPL1	SLC16A3
	PLEKHM3	Q8N7V6	GGTLC1
	LOC100506851	LIN7B	AQP2
	XLOC_002997	XLOC_l2_007834	METRN
	ADCY10	GNMT	LOC646890
	XLOC_003474	PLGLB1	LEFTY1
	XLOC_l2_013853	XLOC_l2_015239	ESAM
	FAM126B	DNAJB5	KIF5A
	NEURL1B	TUBB2A	FAM89B
	RBP5	TSPAN4	RASGEF1B
	PLD2	FARP2	XLOC_001916
	FBXW10	AMH	MUC17
	NRCAM	MBP	GNMT
	XLOC_000951	CLDN23	TRIM41
	NES	LNX1	TNK2
	XLOC_011765	MPZL3	MATN3
	ASB4	IRGQ	CDRT1
	ZFHX2	LOC100506268	LOC91450
	XLOC_003528	XLOC_l2_004854	NEDD9
	SPATA18	TRIM52	LOC645638
	GLIPR2	SHF	TNNC2
	ARG2	EREG	LOC100132439
	XLOC_l2_015033	XLOC_009457	IRAK2
	DCDC2B	LOC100505555	KLHL6
	PTPN13	TMED6	HS3ST6
	XLOC_013535	COX6B2	WDR47
	LIN7B	LOC100132077	LRRC17
	MATN3	CPLX1	ORM1
	RDX	SNHG12	IDI2-AS1
	LOC100130276	PLXDC1	COL8A1
	POLH	LOC283050	LOC440993
	AQP3	DISC1	KRT17
	SLC43A3	XLOC_011480	XLOC_l2_003897
	LOC100130093	XLOC_002133	LOXL3
	XLOC_009683	TMPRSS9	CCDC88B
	PAG1	NKAIN4	FAM53C
	SEMA3F	B3GNT4	FLJ30403
	FLJ33534	MAMSTR	CCDC84
	ZNF75A	XLOC_007262	RETSAT

	IP6K2	LOC100652965	PPARA
	COL8A1	LOC100507305	LOC100132966
	CNIH3	AIF1L	RSL1D1
	C16orf79	ZNF385A	ERVK13-1
	ITGA10	IRF7	STXBP4
	LOC729609	XLOC_006336	XLOC_l2_006751
	S1PR5	HDAC10	CITED2
	RELL1	PPARD	GJB7
	XLOC_004886	RNF122	CTSK
	CLSTN3	LOC100133319	TRAF3IP1
	KRT83	PRSS8	TRIM36
	LINC00336	KIAA1462	TOR1AIP2
	MEGF9	GCDH	SPEG
	XLOC_l2_006751	PIGH	OBSCN
	HLA-DOA	LOC100505787	SSBP2
	ZSWIM6	OXTR	LOC100507614
	FSD1	LMF1	LOC100507278
	CCDC88A	SLC12A7	NOTCH3
	C2orf48	LOC100507419	C4orf32
	C15orf48	SERPINE2	PLIN3
	BBC3	MRPL42P5	CORO7
	ERRFI1	XLOC_012192	CCDC30
	XLOC_l2_005553	IL20RB	ALDOC
	TUBB6	LBH	ABCA7
	APH1B	CCDC116	XLOC_002987
	KLK4	XLOC_012568	FAM90A1
	XLOC_002830	LOC100287177	IGSF8
	MRPL42P5	SLC24A6	H2AFJ
	RNF208	ZNF324	LOC100505787
	XLOC_007368	TNFRSF1B	LINC00173
	TRAF3IP1	SLC24A3	C1QL1
	LNX1	DGCR5	XLOC_000478
	IRGM	SYNPO2L	XLOC_006310
	XLOC_l2_005517	ANXA6	PPP1R15A
	KRT35	LINC00222	DGCR14
	ZNF775	XLOC_012596	LOC100130849
	IL1RAP	BAK1	SMPD1
	ATP6V0D1	ADAM8	EMP1
	C8orf47	ASMT	TTLL6
	EXT1	PELI3	JAK3
	C18orf45	FZD5	PRICKLE3
	LOC100505787	TTC39B	AZI1
	XLOC_002122	FAM102A	RPL32P3
	SLC25A25	PRIC285	TESK2
	DDX25	ECE1	C18orf8
	ENDOU	FAM53C	RHPN1
	XLOC_008781	DGAT1	DCDC2B
	ITPKA	SNORA61	XLOC_000302

	VSIG8	PI4KAP2	TNXB
	CACNA1A	VILL	MAPK8IP3
	FLJ37644	RSPH3	PACSIN2
	BTBD11	XLOC_002122	RTKN2
	RBSG2	XLOC_013370	LOC728061
	CNNM2	DNAI1	C19orf67
	LOC100506802	XLOC_008487	LOC100129148
	BREA2	LOC149773	LOC440132
	WFIKKN2	WWC3	MEF2C
	DGKA	LOC100505551	USP17
	CDC14B	CGB	RPUSD1
	SP8	HLA-A	LOC100505555
	SCPEP1	SPDYA	ACAP3
	NKAIN4	ADAP2	HMOX1
	XLOC_006954	FCHSD1	NINL
	XLOC_l2_008221	TUBGCP6	PXK
	APOC3	MAP1LC3B	POU2F3
	FAM27A	LOC100505760	MAPK11
	CTSK	DHRS9	XLOC_013732
	AQP10	KCTD6	LOC100507025
	ZNF217	NXPH3	SNORA61
	FBXL18	KIAA1549	XLOC_013323
	GAS1	CDKL2	KLHL5
	SPEG	C1orf226	TRIM52
	CRYGS	HIVEP3	HNRNPH1
	MRVI1-AS1	USP17	GIPR
	SLC28A1	RHPN2	SLC22A13
	PPP1R32	FLNA	SPON2
	DNM3	MRGPRF	OXTR
	SLC9A1	PLCB2	N4BP2L1
	XLOC_l2_000018	LOC283887	STK17A
	LOC606724	KCNE2	FAM118A
	GUCA1B	MFGE8	DISC1
	XLOC_l2_011584	LAMB2P1	XLOC_013838
	ABTB2	XLOC_001532	SNORD50B
	BATF3	XLOC_l2_013116	SPTBN5
	RNF39	XLOC_009196	LIPE
	AHDC1	LINC00482	JAKMIP2
	XLOC_l2_009301	FAM3B	SH3PXD2A
	Q29HP5	ARHGEF26	BMP6
	ZNF890P	LOC338620	C1R
	SHC3	C17orf76-AS1	SAT1
	ABCB1	GRHL3	NOS1AP
	RPS24	ACAP3	CHAC1
	PRDM1	NR3C1	PRO0628
	IL1A	C5AR1	ANKRD33B
	ERVK13-1	LOC100652797	SPSB1
	LIN37	PPM1N	TAC3

	AKAP2	PMEPA1	UBE2H
	SERP2	LIPE	C1orf55
	TRIM50	HSD3B1	XLOC_l2_013267
	SEC31B	KDELC1	NXF1
	UBXN10	TBPL1	XLOC_013370
	C14orf45	PPP1R13B	POLH
	C1R	AFAP1	XLOC_008691
	EFR3B	DENND5A	LOC643650
	GRAMD1A	LOC100506802	PI4K2A
	RASSF4	LOC100507563	NKIRAS1
	XLOC_009005	CLDN9	XLOC_l2_013116
	UPK1A	GRIN1	LOC100507445
	TSC22D3	LOC100130713	DLL3
	RNF223	POU2F3	LOC100128857
	ATP1A3	SLC16A12	UBQLNL
	KLF4	NKIRAS1	PPM1N
	XLOC_l2_000706	FAM49A	XLOC_004046
	LDLRAD1	CHRM4	ZFHX2
	MUSTN1	DNHD1	XLOC_013906
	COL23A1	HPSE	XLOC_001099
	SLC45A4	PPL	XLOC_005748
	SLC46A3	LOC200609	LCAT
	NUMBL	TMPRSS7	SYTL2
	ATPAF1-AS1	NPC1	GOLGA2
	PLEKHH3	LOC283070	LOC254100
	FLJ44124	NFKB2	VWA3A
	CHST12	XLOC_006664	GPR161
	GDF1	ST6GALNAC6	BACH1
	IL17C	ZNF697	RELT
	LOC389791	ULBP3	KRT80
	XLOC_002865	NDRG1	HIC2
	PEAR1	SEC61A2	MBOAT2
	SIRPG	CACNA1A	CHST12
	NTF4	MAPRE2	FRMD6
	CCDC114	LRRC31	EVI5L
	LBH	PLA2G10	CD36
	XLOC_014369	PRSS35	DMPK
	C21orf88	XLOC_011769	WNT10B
	MEF2B	FAM40B	XLOC_009146
	GNMT	SOCS1	CFP
	PODNL1	XLOC_008100	XLOC_014247
	PLA2G15	XLOC_014237	LOC283887
	PML	XLOC_004725	LOC644192
	LRFN3	COL6A1	GOLGA6L7P
	TESK2	LMBR1L	NAGS
	SEPX1	LOC100506641	RPL23AP32
	DZANK1	SYTL2	PGM2L1
	XLOC_003417	RFPL1	XLOC_002132

	BIRC3	CARNS1	LOC100132859
	PCOLCE	XLOC_l2_008203	LGI4
	TOR1AIP2	TAC3	XLOC_l2_011244
	NCEH1	C6orf154	ARG2
	MCAM	KIF3A	RPS27
	PLEKHH2	RASSF4	GCDH
	LOC100130930	SMAD7	RFPL4A
	XLOC_009196	LOC100652746	PRKCD
	MFNG	CIB2	TUBB2A
	NHLRC4	CBR3	DGAT1
	XLOC_005413	XLOC_010591	XLOC_l2_014802
	EPAS1	RNF19B	C12orf5
	ANKLE1	NHLRC4	CYP2B6
	CCDC136	PTPN13	XLOC_l2_014579
	TLR5	RAB44	XLOC_010500
	PLXNA3	ACSBG1	XLOC_005963
	ENO2	CDKL4	C9orf139
	LOC100506881	C22orf23	SALL2
	XLOC_001351	SAT2	LOC100131821
	XLOC_008487	C17orf109	ABCA3
	C6orf165	FNBP1	SNORA44
	TIE1	MFAP2	EPAS1
	B3GAT1	NPHS1	DPY19L2P3
	JPH4	RAB8B	MICA
	KLHL17	TTC21A	SOD2
	LMCD1	POLH	CALML5
	PLGLB1	EXD3	LOC100652995
	ENPP5	CCL3	EFNB2
	XLOC_000478	SLC46A1	XLOC_l2_005553
	XLOC_011421	XLOC_009868	SLC25A25
	MIOX	XLOC_012593	FAM73B
	CTIF	LOC100507588	GADD45G
	KREMEN1	XLOC_001243	PAGE2
	PITPNM1	HSPB11	ANKRD13B
	PLCG2	GPCPD1	PLIN4
	ASB2	TTC9B	GPR116
	FZD2	IGFL1	SOAT1
	SESN2	LDHD	LOC100506268
	KIAA1984	KCTD17	SLC5A11
	JAK3	XLOC_000595	PMAIP1
	TRIM15	KLK4	MBP
	EIF5A2	SLC46A3	MIB2
	ANKDD1A	GLI2	C18orf45
	DUOX2	CYHR1	ITPKA
	BAIAP2	GPSM1	LOC441268
	HNRNPH1	IQCD	NFKBIZ
	HLA-DMB	IL4I1	HIST1H1A
	WDR47	ZNF385C	MAFB

	PROC	C17orf67	RASAL1
	BTN2A2	FOXD1	TMEM63C
	PIM1	VTRNA2-1	Q8N7V6
	H19	ADM	SGTB
	C5orf27	SERAC1	FSD1
	C20orf54	SLC43A3	PPP4R1L
	TAC3	PPIP5K1	PCSK4
	CLDN11	UBE2H	KCNH2
	ARID3A	VAX2	XLOC_l2_008221
	EXOC3L4	TCEA2	GDF1
	SSBP2	HSPA2	KRT23
	NANOS2	PRSS53	CRY2
	FSTL3	LOC100129399	DACT1
	HS3ST6	XLOC_002277	LOC100128402
	XLOC_012288	CCL27	C7orf61
	STARD5	XLOC_013981	XLOC_013549
	IL11RA	JAG1	SLC2A6
	LOC143666	SLC16A3	SLC22A4
	CASC1	MGC4294	IZUMO4
	sept-04	ALDH1A3	XLOC_010114
	ZMIZ2	COL28A1	MRPL42P5
	RIMS3	XLOC_009191	MAPK8IP2
	JAG2	LOC100652777	XLOC_012593
	LOC100653515	LOC100652768	LOC338653
	XLOC_008691	ANKDD1A	XLOC_012254
	SLN	PI4K2A	CX3CL1
	PGM2L1	VTRNA1-3	ITPRIP
	CBX2	EML6	CDC14B
	TIMP2	PNPLA2	TRIM31
	PNPLA3	KLHL17	VPS53
	LOC100294362	FLJ39095	C6orf222
	AGFG2	C10orf129	PLEKHM3
	SGTB	LOC100288432	XLOC_013100
	C12orf5	SYT7	ASMT
	KLHL25	FAM85A	RAB24
	XLOC_004607	SYCE2	TOX2
	GSDMB	CLEC18B	ADSSL1
	ADCY4	C12orf5	NEAT1
	VWA1	XLOC_006513	STARD5
	KIFC2	XLOC_007205	PLA2G15
	CXorf41	KCNMB4	SLC24A6
	XLOC_l2_005179	LILRB3	SOBP
	RHBG	XLOC_006178	XLOC_013905
	RAB6B	PNMA2	LOC100652768
	SCARA5	PLEKHO2	FAM182B
	NEBL	JUND	XLOC_002830
	LOC619207	GRK5	LOC143666
	XLOC_002356	SNORA14A	NUAK2

	JAKMIP2	LOC100506047	LINC00310
	XLOC_010500	ARHGAP26	AQP10
	XLOC_006721	DGKA	XLOC_006941
	TRIM46	TRIM4	PLCXD1
	XLOC_009181	TTLL13	LOC100130930
	LOC100131581	PLB1	CCDC148
	XLOC_010599	ZNF556	MUSTN1
	VKORC1	RASGEF1A	WTAP
	PLA2G4E	XLOC_l2_014421	JAG2
	CRY2	SERPINA5	ASB6
	LGI4	ALDH8A1	IP6K2
	FXYD7	CABYR	S100A3
	LOC440993	VSIG8	CACNA1A
	LOC729652	PRKCD	KCNC3
	TCEA2	RPL23AP32	SLC43A3
	WISP2	BMP2	LOC100506802
	LOC100507025	LOC285768	CDH3
	HABP4	GDF11	FAM55C
	N4BP2L1	CBX2	C11orf94
	ATAD3C	KLHL5	Q6BWL2
	NR1H4	LOC728802	ZNF75A
	XLOC_005223	XLOC_005039	HCN3
	PLEKHG5	GAST	RAB8B
	XLOC_012192	CTSK	FZD4
	SBSN	ARNTL	NUMBL
	GP6	SLC31A2	ST6GALNAC4
	WNT5B	SEC1	LOC100507305
	CDKN2D	TMEM206	CMTM2
	FAM102A	XLOC_001338	METRNL
	CHP2	ZDHHC20	GATS
	LOC731789	XLOC_l2_009883	C9orf72
	NRGN	ZDHHC11	HUS1B
	LOC100289488	IRF8	PLGLB1
	PAPD5	CT45A5	CLDN19
	XLOC_007189	TRIM15	UNC119
	JAKMIP3	IRF1	XLOC_001338
	ZNF540	RELT	XLOC_009943
	LOC100506418	C10orf54	DIXDC1
	VPS53	TSC22D2	LOC285141
	XLOC_l2_014802	PLEKHH3	XLOC_002277
	CHKA	WNT6	LOC285147
	CLEC18B	ITGB7	DDIT4
	XLOC_003457	MAPKBP1	ARNTL
	XLOC_012021	RPS24	AGFG2
	ARID3B	PAGE2B	TPRN
	LOC148638	CCDC19	MAP4K2
	SLC24A3	ZNF775	PHF1
	RFPL1	INSL4	XLOC_012021

	NR3C1	PALM3	LOC389791
	LOC100129268	XLOC_011413	RAB11FIP1
	XLOC_002515	C17orf96	XLOC_012912
	PTGER4	GLCE	CDH16
	INPP5J	C10orf114	CD70
	CLIP2	INPP5K	JUND
	XLOC_l2_012847	FAM161B	C17orf67
	FANK1	N4BP2L1	LOC100131796
	LOC100507305	LOC644242	COX6B2
	ACTA1	FAM131A	TOR1B
	ST3GAL4	XLOC_l2_002171	RAB36
	KCNE2	GSTM4	RARA
	LCA5	LOC100128292	PNPLA1
	DISC1	MFSD4	XLOC_009683
	KIAA1467	RDX	XLOC_013955
	MYBL1	C7orf57	SNORA43
	DENND3	KISS1	LOC440792
	CDH3	PIGR	STRC
	SYTL2	PORCN	TNFRSF6B
	HOXC13	EEF1DP3	C15orf62
	MYOM1	VLDLR	MMP13
	CPLX3	SLC26A1	PHYHIP
	TOX2	LAG3	SPIRE2
	TTYH2	CYP1B1	XLOC_l2_012748
	PAX7	CCDC114	IL27RA
	TCAP	DENND1C	XLOC_013218
	DKFZp686M1136	LRFN3	LOC100289424
	ULBP2	AIM1L	CD74
	GRASP	LOC100294362	OTUB2
	DNAI1	CLEC3B	SYTL3
	BCAS4	SLC25A25	XLOC_005341
	SORBS1	RFX2	LOC100505551
	C1S	NEDD9	PLXNA3
	DOCK4	MMP15	XLOC_l2_009301
	ASMTL	COL8A1	XLOC_001457
	ULBP1	C7orf52	STMN4
	SCIN	SLC2A5	SGK1
	DIXDC1	CFP	TSGA10
	XLOC_003629	SH3PXD2A	TERF1
	CDH16	TSPAN9	XLOC_003417
	ZNF703	XLOC_003734	JPH4
	KANK3	SERHL2	XLOC_013932
	SLC28A2	ITPR1	CACNA1G
	C10orf114	INHBB	KIAA1462
	WNT6	TSPAN10	XLOC_002131
	SLC25A34	XLOC_l2_009292	FBXL18
	XLOC_008809	LHX1	CCDC136
	PELI3	XLOC_011350	KLK8

	PFKFB4	LOC100130547	PDGFB
	DNAH12	IL32	BTN1A1
	ARG1	MLLT11	CDX1
	LHX4	APLP1	MEF2B
	PLCD4	XLOC_009487	LOC440934
	KLK13	XLOC_l2_001537	SLC31A2
	ADAM11	LOC344595	IL1RAP
	LOC644192	XLOC_l2_001134	EID3
	XLOC_009146	MYO1E	TNFRSF25
	LEFTY1	SH2D1B	ITGA10
	HIVEP3	LOC401052	XLOC_007368
	LOC285141	PLXNA4	TUFT1
	HIC1	ALAS1	PLD2
	TMEM52	USP35	PLEKHH3
	UBTD1	NFKBIE	LOC100506001
	TTLL6	SPAM1	AP3B2
	LOC100506252	LOC440330	FAM90A7
	COL28A1	TEX14	OASL
	SLC25A27	BCO2	LOC100287415
	C17orf67	EFNB2	MYO15B
	HIST2H2BF	GDPD1	FAM49A
	HVCN1	FUT7	IGFALS
	LOC100128292	Q9DSJ7	PDXP
	FILIP1L	IDS	PROC
	sept-01	TLE6	IQUB
	SLC22A13	WHSC2	NHLRC4
	CCDC146	GSDMB	TRPC1
	NINL	LOC284551	INPP5J
	XLOC_011559	XLOC_l2_007427	MFSD4
	GLRX	RGS16	XLOC_004165
	sept-03	TRAF3IP1	ELF3
	HIST1H1T	XLOC_002085	CGNL1
	CCDC42B	TBX19	IRF7
	CCDC153	LOC100499221	CALCOCO1
	AOC3	PLXNA3	FCRL5
	MBOAT2	ZNF519	DLX4
	DMBX1	FLJ42392	VTRNA1-2
	LOC100287177	KCNN4	NKAIN4
	ADRB1	XLOC_003417	XLOC_l2_007783
	LOC100506854	WDR96	XLOC_001351
	CABLES2	HLF	IL1RL1
	HCN3	PLD2	FLJ45983
	LOC100287082	KCNH2	TSNAXIP1
	TAOK3	CLDN11	SORBS1
	ST6GALNAC6	POR	TMEM236
	DLX4	XLOC_000011	SOCS1
	LOC729444	FYN	COL28A1
	LOC100507419	LOC100132354	CLEC16A

	MFSD4	XLOC_013838	CA4
	ARHGAP30	VCL	GABRE
	PCYOX1L	ZNF711	XLOC_014402
	KCNK7	MEF2B	ATXN1L
	XLOC_013218	DAPK3	LOC100133190
	NLGN2	VPS53	RPPH1
	MAMLD1	FAM115C	SIRPG
	IL36G	GDPD5	ARHGAP26
	LOC283484	RGS9	N4BP2L2
	TOM1L2	CACNA1G	XLOC_014047
	TNNT1	ST6GALNAC4	GRAMD1A
	DUSP1	XLOC_008466	LNX1
	XLOC_002296	CCDC136	XLOC_008679
	SLC31A2	LOC100507445	GSDMB
	XLOC_l2_002204	CNPY4	SEMA3F
	SOAT1	XLOC_006721	KCNN2
	CALCOCO1	EHD1	XLOC_000822
	AK7	SCN4A	XLOC_007504
	MAPK8IP2	PACSIN1	XLOC_000508
	RASAL1	KCNJ10	LOC100506881
	SAMD14	DEFB1	XLOC_012568
	CPEB4	TMEM229B	INPP5K
	RSPH4A	PRRT4	LOC387895
	NEU1	PLIN1	FLJ26086
	PI4KAP1	GLI1	ANKLE1
	XLOC_006150	XLOC_l2_005553	HMHA1
	XLOC_001134	SLC4A9	C20orf96
	TRPC1	LOC100288092	TAS1R3
	XLOC_010194	DLL3	XLOC_008809
	C7orf53	TNK2	BTN2A2
	LOC100289211	MERTK	OSM
	NOTCH3	ABCB1	PNPLA3
	LOC100131355	SPSB1	AOC3
	XLOC_001457	CSDC2	LOC338620
	CLEC16A	NOS1AP	GLIPR2
	TMCO2	XLOC_000102	TRIM50
	LOC100505679	PRKCA	FBXW10
	SUFU	MICA	LILRB3
	SPDYA	PPP1R15B	LOC90246
	DLL3	LRRC8E	LCA5
	RAB36	CORO7	EXT1
	PRSS53	CNNM2	HIST3H2BB
	XLOC_004584	LGALS7	LRFN3
	CA4	LOC100130987	SKI
	KCNH2	ATG2A	FRY
	USP49	CDKN2D	DKFZP564C196
	LOC344595	TNFAIP8L2	XLOC_001048
	SLC22A4	SGCA	CCDC116

	MSX2	GADD45A	XLOC_010194
	RETSAT	PRODH2	KIFC2
	NOVA2	LOC643201	XLOC_012946
	CT45A5	ATP8A1	XLOC_009398
	LOC100505566	RAET1K	XLOC_008149
	SLC4A3	NTNG1	XLOC_000214
	MSX1	LOC283588	XLOC_011633
	FZD4	MOGAT1	XLOC_003327
	CYR61	ASCL1	KIAA1984
	C12orf34	CNGA1	LOC100130093
	EFEMP2	LOC645195	PLEKHG5
	H1F0	C2orf48	XLOC_013772
	XLOC_000595	KLHL15	XLOC_009526
	EFNB3	TPRN	ZC3H12A
	MEIS3	SIRT2	XLOC_013301
	N4BP2L2	AHDC1	IL11RA
	XLOC_002918	XLOC_003872	NEU1
	XLOC_002033	TNFRSF25	C6orf81
	LOC100129148	XLOC_l2_009301	XLOC_009752
	FRMD6	TRIM50	FOXD1
	RUNDC3B	MTSS1L	ASGR1
	RPL37A	STARD5	CSDC2
	SYTL3	XLOC_l2_012323	PTHLH
	PARD6G	PPARA	XLOC_001074
	SIPA1	MPP2	XLOC_007205
	XLOC_l2_006080	HVCN1	SNORD101
	SLC25A41	LOC646999	sept-03
	LOC100507233	DAPK2	GTPBP3
	FAM3B	MAPK15	LOC100506047
	PAGE2	EMP1	FLJ42392
	GJB5	XLOC_013712	GP6
	ADARB1	XLOC_002830	C16orf79
	CAPN5	AQP3	XLOC_014219
	CNIH2	SGK223	KLRG2
	MAST1	LOC100507639	SNORD99
	LOC646890	DOK3	RASD1
	PPAPDC3	XLOC_004046	ERRFI1
	MCF2L	CSGALNACT2	XLOC_013602
	KIF25	LOC442028	TOM1L2
	LOC100506025	TTLL11	BMP2
	NAP1L5	HNRNPH1	GDF11
	FKBP14	FAM71A	XLOC_008678
	XLOC_003427	GDF15	LOC619207
	LRRC17	SPRR1A	ZNF775
	C2CD2L	INPP5J	NCEH1
	ESPN	PTK2B	NCF1
	C17orf96	PLCXD1	TNNT2
	DPP6	SRPX	WNT6

	OTP	XLOC_l2_011291	CRYM
	LRRC24	XLOC_011331	NPAS3
	MYZAP	BBOX1	MAST1
	STARD9	GPRIN1	XLOC_009183
	HAS3	NFIL3	KANK3
	C14orf37	TMEM170B	CDC42BPG
	IQUB	XLOC_009943	ZNF217
	KCTD17	NCEH1	XLOC_000755
	CGNL1	MEGF9	TMEM86A
	IL4I1	CLSTN3	LOC100130157
	C7orf63	TNNT1	C22orf31
	FMNL1	PRKCG	TCEA2
	FAM153B	UNC119	AGPAT4-IT1
	GLI1	LINC00173	CNGA1
	CDR2L	SLC22A13	MYBL1
	ACAP1	CD109	KLK13
	NPAS3	RETSAT	DGKA
	XLOC_l2_003674	KIF5A	IRX4
	AP3B2	MUC4	KLHL17
	BMP8A	CD274	HIVEP3
	ASAP3	ZNF815	CTIF
	CCDC168	SALL2	ZNF296
	DLX1	CEBPB	XLOC_011287
	LOC100507131	TANC2	COLEC11
	ABCB9	XLOC_l2_007928	ADAT3
	ATG2A	LOC100289488	XLOC_008441
	LOC100506001	DCDC1	ZSWIM4
	FOSB	PHYHIP	CSRNP1
	CNNM4	TMC8	CCDC146
	CLEC3B	PNPLA1	PTGER4
	DENND1C	XLOC_013268	XLOC_l2_005517
	RASIP1	JAKMIP3	TIMP2
	XLOC_000340	TMEM52	XLOC_010315
	XLOC_011766	LOC728978	RAB34
	MMP13	OVGP1	DISP2
	GRK4	SERPINE3	MAK
	TMEM63C	KCNN2	FAM102A
	MAN1C1	RAB30	DUOX2
	PLEKHF1	OSTBETA	NES
	GDPD3	PITPNM1	XLOC_002122
	LIPC	MUSTN1	RASIP1
	XLOC_001243	SOX8	GOLGA6L6
	EPHB2	KLHL25	XLOC_002997
	NFATC4	ALDOC	NR1H4
	CCDC148	XLOC_l2_000706	H1F0
	LOC338620	PFKFB2	PRSS35
	USH1G	METRNL	TSC22D3
	XLOC_010275	PLEKHM3	EPPK1

	PLEKHO1	SNN	ADM
	XLOC_013301	CCDC153	KRT83
	SPAG8	MIR7-3HG	CNNM4
	RAD9A	RAD9A	XLOC_010945
	LINC00173	ANKRD42	ENO2
	BLVRA	MID2	MYLK2
	MAMSTR	FAM126B	LIN37
	XLOC_007776	TMCC3	XLOC_002085
	CD36	ESAM	XLOC_011563
	TMEM158	GUCA1B	PIGR
	ALDOAP2	GSTM1	XLOC_012288
	XLOC_010942	LOC100289187	PELI3
	IER5	TNFRSF10B	XLOC_000340
	CLEC4A	PLCG2	XLOC_003881
	RFX2	FAM126A	MZB1
	ADAMTS7	SDC4	CDON
	VWA5B2	NINL	XLOC_l2_014820
	DCDC1	LOC100652915	RGS16
	XLOC_000102	RAB3B	ACHE
	LPHN1	CYFIP2	XLOC_004361
	DACT1	GRHL1	FAM126B
	XLOC_000822	LOC100289211	EFNB3
	EVI5L	PLCE1	ESPN
	XLOC_005082	WDR47	DCDC1
	PI4KA	MAST1	XLOC_010601
	LOC285147	MUC12	ZMIZ2
	SPTBN4	SLC9A5	RIIAD1
	FAM55C	NPHP1	FMNL1
	XLOC_012339	SPRED3	LMBR1L
	VAX2	XLOC_002132	IZUMO1
	C16orf54	SPTBN4	LOC100130276
	PRSS35	DISP2	LOC100652769
	RORA	PRRT2	ASMTL
	FLYWCH1	HCN3	LOC100506219
	AQP7P1	LOC100289090	LOC100652740
	LOC100506791	MYBL1	GLI1
	PRKCG	OASL	H19
	IL16	LRRC3	CT45A5
	BTNL8	RPL32P3	SLC38A4
	SLC4A11	FZD4	KREMEN1
	XLOC_l2_012942	PLA2G15	XLOC_005082
	LOC100130987	DGCR14	RPS6KA2
	TNNC1	H1FX	LOC100129999
	CDON	LOC100131726	HABP4
	WWTR1	ENTPD2	EPHB2
	PTGES	FUT1	ABCB9
	NACAD	PPP1R15A	BTNL8
	MPP2	NLGN2	GGT8P

	RHEBL1	KCNK7	SUFU
	XLOC_012593	ATPAF1-AS1	CLEC4A
	FOXJ1	FAM55C	SH2D1B
	SPAM1	GPR153	CABLES2
	FGF22	RAPGEFL1	FKBP14
	DSG3	HES2	XLOC_l2_001947
	MSX2P1	LOC90246	PIM1
	ABHD8	PLEKHH2	DOCK4
	UNC5D	XLOC_l2_010602	NAP1L5
	MYBPHL	KREMEN1	CYP46A1
	EEF1DP3	LRRC24	ULBP2
	XLOC_000302	BTN2A2	DKFZp451A211
	SPRED3	EPPK1	XLOC_014418
	BCL6	XLOC_014369	LPHN1
	GARNL3	NR1H4	MCAM
	FAM115C	CRYGS	TTC16
	CALHM3	MAP3K14	XLOC_012258
	VAV1	RELL1	PACSIN1
	CD109	SPAG8	LMCD1
	CRYM	XLOC_008809	XLOC_l2_011798
	SNN	RIMS3	CBX2
	HEY1	SLC26A11	ATP6V0D1
	FLT3LG	SLC22A15	AEBP1
	C7orf57	XLOC_l2_003133	RPS24
	GJD3	TNFAIP3	XLOC_012456
	S100A1	AGT	LOC729652
	DISP2	NES	XLOC_l2_011291
	BMP6	COL5A3	ANKDD1A
	SH3GL3	LOC100131366	MYZAP
	ARHGEF25	PRDM2	RASL11B
	LOC151300	PLEKHO1	PLLP
	LOC100505983	LOC100506124	EIF5A2
	C20orf160	XLOC_l2_014820	EHD1
	FUT7	C9orf174	LOC100505679
	MYBPC1	MCAM	ST3GAL4
	C4orf47	KIRREL2	CPLX3
	PNMA2	GLRX	LOC100131726
	PRRT2	VEGFA	ARHGEF4
	KCNK4	FAM73B	XLOC_010591
	MIAT	LOC100129171	XLOC_008487
	IGSF9	SPOCD1	FLYWCH1
	XLOC_004361	XLOC_001351	LOC400548
	MOGAT1	GFOD1	SERPINC1
	CCR7	RIIAD1	RFX2
	XLOC_000670	DUSP8	CBR3
	CADM4	KIF21B	ZNF815
	PXK	PTGER4	RASSF4
	GLS	LOC151657	CDKN2D

	CCDC96	C14orf45	XLOC_006249
	FP588	CD52	XLOC_l2_000792
	RBFOX3	PDXP	BCL6
	HSPB8	AK7	LOC100507639
	GPNMB	KLHDC8B	XLOC_009880
	KIAA1683	IL17C	XLOC_010245
	SH2B3	LOC606724	ST6GALNAC6
	XLOC_009274	PARD6G	SCARNA11
	XLOC_012258	C7orf63	LINC00202
	ELFN2	TRPC1	C1orf190
	PRODH2	DKFZp686M1136	XLOC_000111
	ABCA3	DUOXA1	C20orf54
	CSDC2	HOXC13	EGR2
	AMICA1	L1CAM	COL23A1
	LOC284688	ERVK13-1	AHDC1
	C6orf81	AGPAT4	GRIN1
	TSNAXIP1	ACAP1	MIR7-3HG
	DUSP10	ZNF491	TNNT1
	RASGEF1A	PLCD1	LOC100499221
	LOC340335	C9orf72	DAPK3
	XLOC_014402	PKIG	XLOC_l2_000804
	LOC100288432	GPR124	XLOC_010599
	RNF24	XLOC_000421	SLC25A34
	MATN4	FKBP14	XLOC_014369
	PLLP	BTBD11	XLOC_006994
	DUSP5	C18orf45	PCOLCE
	TBC1D10C	ACTA1	CD164L2
	CNGA1	SKI	ARID3B
	ABCG1	GNG3	MYBPC1
	KALRN	XLOC_001788	XLOC_007191
	TMEM45A	CCDC80	KRT35
	GNG3	SPIRE1	GLS
	UBQLNL	H1F0	SESN2
	ATCAY	SIPA1	AKAP2
	PREX1	C11orf94	DENND3
	RIIAD1	IGSF9	ZNF720
	GPC2	SLC4A3	ATPAF1-AS1
	SH3PXD2B	ARHGEF25	SEPX1
	LOC100129999	OSBP2	LOC100128292
	CNPY4	LOC100129999	HES5
	SLC2A5	HKDC1	PALM
	TMEM86A	FSTL3	LOC100507563
	SUSD2	SOX2	ATG2A
	GCGR	SMOX	ARID3A
	BEST1	STAB1	CHKA
	LOC254896	SLC45A4	HAS3
	LOC286299	SCPEP1	SNORA14A
	XLOC_004606	MAN1C1	CYP2U1

	CMTM2	XLOC_005488	LOC728705
	IL11	Q93YZ4	AQP3
	NAALADL1	XLOC_002918	KCTD17
	LPPR2	MRVI1-AS1	XLOC_001243
	GATSL3	XLOC_008223	PITPNM1
	C7orf52	GLS	SNN
	XLOC_000101	C1QL1	CCDC153
	PACSIN1	AREG	SIX1
	PPP2R5B	SLC22A4	CYP1A1
	HTR7	LOC497257	PFKFB4
	EPHA3	NGEF	HBEGF
	XLOC_011284	XLOC_009684	CLEC18B
	LOC100506662	DIXDC1	CD52
	ADAM32	KLHL26	FP588
	KCNJ10	ST3GAL4	CAPN5
	XLOC_l2_001947	PALM	XLOC_014096
	DYNLRB2	GCNT1	UBTD1
	LOC283404	LOC100130357	NFATC4
	ST3GAL5	TCAP	SLC4A9
	XLOC_014336	LCAT	ELFN2
	DBP	ADA	LOC100287082
	LOC153577	IER5	C7orf52
	XLOC_l2_014369	SEMA3F	NR3C1
	LOC646851	PLAU	NPHS1
	IZUMO1	GCGR	RNF122
	WFDC10B	ST8SIA2	TMEM52
	LGALS7	FAM65C	NFATC2
	BCO2	XLOC_l2_000961	RNF39
	BIK	XLOC_002997	PTGIR
	XLOC_l2_011291	CASC1	AMPD3
	SLC30A4	VAMP1	CNIH2
	LINC00312	XLOC_012360	ADCY4
	XLOC_l2_013835	OR51A2	GARNL3
	DENND2C	XLOC_010194	SNPH
	C8orf31	BLVRA	NRGN
	SNPH	SLC25A27	XLOC_001198
	LOC400548	SLC9A3	LHX1
	XLOC_009167	DAPL1	DKFZp686M1136
	HEXIM1	BMP8B	VWA5B2
	LINC00202	PLEKHF1	GLRX
	XLOC_005927	FGF22	RAD9A
	XLOC_011040	CGNL1	BLVRA
	RBM44	SLC6A13	FLJ44124
	XLOC_l2_000804	SOBP	XLOC_010557
	LOC100133669	MOGAT2	CCDC114
	SOBP	CCL20	LOC149773
	PPP1R14D	GABBR1	SPAG8
	XLOC_l2_010386	XLOC_013711	NLGN2

	XLOC_013559	XLOC_011827	HEY1
	HIP1	DOCK4	ASAP3
	LOC100131726	XLOC_009576	FSCN3
	LRRC43	TAOK3	CYTH4
	PDZD7	XLOC_l2_003295	ARHGEF25
	CD164L2	ATP8B2	HOXC13
	CYP46A1	XLOC_004606	IL8
	SYNGR3	HIPK4	XLOC_002400
	MUC15	ASMTL	FGF22
	ITGA7	CCDC148	ADAM32
	XLOC_002958	RHOF	C10orf114
	LOC100505573	HIP1	RNF223
	LARP6	DMPK	C7orf63
	OOSP1	SPIRE2	C7orf53
	CDKN1A	PFKFB4	TRIM15
	C19orf71	CREB5	MFNG
	NAV2	C18orf8	XLOC_008823
	LOC200609	LOC728705	BATF3
	ProSAPiP1	XLOC_003854	DENND1C
	XLOC_l2_008599	SPTSSB	XLOC_l2_013846
	CCDC40	XLOC_013602	LDHD
	C10orf129	PIRT	LOC100507419
	XLOC_011949	CCDC146	EVC
	PDE4A	C12orf34	XLOC_l2_010386
	XLOC_009398	LPAR5	XLOC_l2_012323
	DLX3	LRRC43	DNAH12
	XLOC_010470	CYP26B1	HPX
	SNTA1	ARID3A	LOC374890
	VAMP1	SLC30A4	HTR7
	XLOC_003854	ANKRD29	NGFR
	CCDC28B	LOC349160	XLOC_l2_010558
	PLCD1	TMEM236	LOC441204
	TMC8	XLOC_000388	XLOC_011284
	TMEM236	ASGR1	ENTPD2
	NKX3-2	XLOC_000101	TPST1
	RCOR2	FRMD6	DMBX1
	RNASE1	AKNA	KLK4
	XLOC_011592	XLOC_013100	XLOC_013578
	XLOC_007515	NFATC4	C20orf160
	RFPL2	XLOC_010542	XLOC_000102
	Q9BVX4	PODNL1	LPHN2
	LYST	ADAMTS17	MSX2
	XLOC_006879	CLEC4A	ALPPL2
	TLE2	PPARGC1A	LOC100130987
	C1orf190	SBSN	PLK3
	XLOC_009016	NEURL1B	CXCL10
	SRPX	TMCO2	CLIP2
	XLOC_012596	KIAA0319	CLEC3B

	LOC100506219	TIGD3	WDR96
	TFCP2L1	SLC23A2	TAOK3
	EPOR	HEY1	HIC1
	LOC100505576	FLRT1	LPPR2
	PGF	FMNL1	WNT5B
	LOC284939	BTNL8	KLHDC8B
	PTGIR	PNPLA3	ADAM11
	PLA2G4D	MSX2	XLOC_010607
	PALM	SAMD4A	OTUD7A
	XLOC_013467	C19orf51	XLOC_002515
	LAG3	S100B	USHBP1
	KIAA0509	MATN4	VAX2
	HSPA2	GDF1	XLOC_l2_009884
	MITF	LOC100133190	RNF208
	SELM	C20orf195	LARP6
	KLHL29	KIAA0509	FAM115C
	XLOC_006951	SLC16A10	XLOC_013968
	XLOC_006195	C4orf32	NANOS3
	ARC	ERN1	HIP1
	PCK1	LCA5	PDE4A
	SEMA6C	TIPARP	VSIG8
	HES4	ENO2	SNTA1
	XLOC_l2_004631	POLN	BCO2
	XLOC_002132	CCDC96	LRRC6
	CROCCP3	FILIP1L	SESN3
	RND1	LOC399715	HCG22
	XLOC_l2_012323	LEPR	LOC283435
	AQP7	NLRC5	CYP2F1
	CCDC30	PLLP	LOC100289488
	XLOC_011597	LOC729609	XLOC_010262
	SPTSSB	NFATC2	OSBPL6
	MICALCL	SEMA3G	LOC100131043
	HSD17B3	LILRB5	SLC9A1
	XLOC_l2_007274	GRASP	XLOC_009452
	ATG9B	B3GAT1	TMEM25
	LOC731779	HABP4	BCAS4
	OSTBETA	SLC6A12	GJB5
	FNDC4	DZANK1	TSPAN10
	SH2D5	CDH3	IL16
	XLOC_000695	CELSR2	VEGFA
	GPR146	SSBP2	LOC100134285
	XLOC_l2_007802	LOC644192	XLOC_l2_011873
	XLOC_002788	RHOB	XLOC_001628
	WNT4	LAMP3	TLE2
	MAPRE3	XLOC_009146	WFDC10B
	SLC22A16	CD164L2	CNPY4
	SIX1	KALRN	SPRED3
	TTC29	TTYH2	XLOC_l2_001064

	KRT86	AMHR2	AVPI1
	LOC100505473	IGSF8	RBM44
	ALDH8A1	EPAS1	IDS
	ENTPD2	SGTB	SIPA1
	BMF	AHRR	XLOC_013712
	ICAM5	NRGN	XLOC_l2_011007
	CACNA1G	ZNF541	ZDHHC20
	XLOC_010506	TRIM36	XLOC_014137
	LOC100507639	LPPR2	WDR66
	HTR7P1	ERRFI1	NEBL
	XLOC_004534	C15orf62	XLOC_002918
	XLOC_006398	FAM90A7	CCDC96
	BCORL1	MYOM1	C8orf31
	XLOC_003907	HTR7	FOS
	ARL4C	LOC646851	STAB1
	LOC728705	ASNS	TMEM158
	FXYD4	TMEM45A	LRRC24
	S1PR2	CSF1R	SLC45A4
	S1PR4	CSRNP1	BAIAP2
	CYP2E1	XLOC_011513	XLOC_012114
	LOC100652843	GBX1	LOC100505894
	TMEM25	XLOC_l2_001947	RHOB
	FAM46C	RHEBL1	SLC4A3
	C8orf60	XLOC_l2_007204	LOC100505573
	XLOC_009224	JAG2	LOC100507131
	KRTAP5-8	DOC2GP	ADRB1
	LOC643201	SEMA6C	FILIP1L
	XLOC_012891	KSR1	PI4KAP1
	ZBTB46	RSPH4A	CROCCP3
	C3orf32	CCDC88A	NAALADL1
	XLOC_l2_011798	CCL21	XLOC_010715
	XLOC_004557	IRGM	ZSWIM6
	LOC400927	IL36G	XLOC_007776
	LOC388242	ENTPD8	XLOC_l2_014369
	XLOC_009601	XLOC_011278	C17orf96
	RHOXF1	EMID1	FAM85A
	XLOC_010945	HTR7P1	MCF2L
	DUSP5P	NEU1	Q29HP5
	TPST1	AVPI1	OOSP1
	COLEC11	NUMBL	LOC100507800
	LOC648149	SLC7A5	XLOC_l2_004317
	NGFR	MCF2L	C6orf25
	NTNG2	CYP46A1	CRYGC
	LOC100506124	FLJ44124	PNMA2
	C20orf195	ESPN	NCF2
	XLOC_006902	MEIS3	GDPD3
	LILRP2	NTNG2	XLOC_000951
	PALM3	CABLES2	CD83

	SLC6A13	KSR2	XLOC_l2_010083
	APOE	OR2AK2	RBP5
	LOC100505869	XLOC_010942	ADCY9
	XLOC_002872	XLOC_007504	PRSS53
	LOC100507430	HMOX1	XLOC_002069
	XLOC_013712	SESN3	XLOC_006844
	CERCAM	PTHLH	POU2F2
	ENTPD8	N4BP3	C8orf66
	MAP1LC3A	CTIF	LOC643201
	CITED4	XLOC_009797	C2CD2L
	IQCJ-SCHIP1	LOC284939	FTCD
	EGFL7	TLE2	KLHL25
	ADM2	ITPKA	LOC100506662
	LOC100288911	LOC100507347	S100A1
	HBA2	LARP6	LINC00051
	ANKRD42	MBOAT2	C13orf35
	MYO7A	ARG2	KIAA0509
	GPR123	EPHB2	CEBPB
	CERS1	XLOC_010743	SH3PXD2B
	XLOC_008466	LOC100653515	XLOC_007884
	CYP1A1	CAPN5	XLOC_008696
	C19orf51	DENND3	XLOC_004584
	XLOC_000256	OTUD7A	C12orf34
	ALPPL2	EFEMP2	KRTAP5-8
	ARID5A	BCAS4	LOC100128262
	TNFAIP8L2	LOC728175	XLOC_010374
	XLOC_007966	TTLL6	CPEB4
	XLOC_002402	EPOR	LYST
	SLC26A11	XLOC_012567	RHOF
	C1orf170	RHOXF1	RASGEF1A
	SLC38A4	TUBB6	KIF6
	LRRC6	MAK	LOC100507233
	OR2T34	IZUMO1	XLOC_006195
	SLC29A4	ITPRIP	AMICA1
	XLOC_002821	ZFHX2	ATP1A1
	GPR161	TMEM98	CCDC168
	KLRG2	C2CD2L	RIMS3
	PER1	SLC25A41	ABHD8
	WFIKKN1	LOC100616668	XLOC_005579
	FAM43A	ADRB1	GRK4
	XLOC_013993	CCDC28B	HTR7P1
	ADCY9	CLIP2	GJA3
	C12orf39	XLOC_009279	MAP1A
	XLOC_010385	AP3B2	XLOC_004636
	CYTH3	SYTL3	XLOC_010275
	TSPAN10	C14orf37	IL17RD
	LOC283553	RAB11FIP1	NTNG2
	XLOC_l2_012678	CYP2G1P	KALRN

	XLOC_l2_007204	MGC50722	LOC153577
	XLOC_l2_010018	XLOC_011287	HTR3B
	SALL2	XLOC_004557	SLC26A11
	KLF9	PMEL	XLOC_013711
	MPP1	SH3BP5	OR2B6
	LOC100130800	XLOC_l2_014504	ProSAPiP1
	XLOC_001431	GAMT	PAPD5
	CDH15	SPON1	XLOC_009601
	XLOC_l2_013153	XLOC_012021	XLOC_l2_003133
	XLOC_002131	ITGA7	GPC2
	XLOC_014226	XLOC_002131	CERCAM
	XLOC_l2_001097	ATP1A3	PAGE2B
	RAB3A	USH1G	XLOC_003688
	XLOC_005128	LOC100133669	PALM3
	ZNF815	BIK	RORA
	BDKRB2	SGCE	FAM71A
	FTCD	ULBP2	FBP2
	METTL20	MEI1	GSTM2
	XLOC_011278	C16orf79	IER3
	DBNL	FAM90A1	LOC100287177
	XLOC_011769	XLOC_009458	XLOC_008652
	XLOC_006219	SECTM1	XLOC_009797
	XLOC_009948	XLOC_000478	CBX4
	CCK	CPLX3	XLOC_l2_011043
	CYP2U1	PI4KAP1	SLC4A11
	SCHIP1	EFR3B	HEXIM1
	LOC100169752	KLF4	RNASE1
	ARAP3	LOC100131551	LOC100133669
	SERPINC1	ATAD3C	XLOC_002961
	PBX4	BCORL1	XLOC_003854
	NDRG4	CYP2E1	SPAM1
	KLHDC8B	AOC3	TCAP
	FBXO27	RASAL1	LOC100131829
	XLOC_l2_002171	CAMTA1	IGSF9
	NHSL2	FLT3LG	LOC100507959
	XLOC_l2_003974	XLOC_013702	IRF1
	XLOC_l2_003133	NOTCH3	CSF2
	LOC151657	PTGDS	KCNK7
	LRFN1	PDLIM7	SHANK3
	C14orf49	LGI4	PTGES
	LOC100129119	GPC2	FAM69B
	KIF21B	MDGA2	XLOC_005101
	AKD1	SAMD14	NFE2
	TIGD3	SPEG	XLOC_010542
	COL5A3	XLOC_003820	XLOC_l2_000033
	SPHKAP	P2RY12	CDH15
	XLOC_007884	LRRC15	LTB
	AHNAK2	LOC648149	SLC25A41

	PPM1J	LINC00312	LRFN1
	XLOC_000035	LOC100131796	LOC100506124
	XLOC_006892	BBC3	ABTB2
	KC6	PGLYRP1	TNNC1
	XLOC_010200	LOC100505573	XLOC_l2_010056
	CFD	XLOC_010945	CADM4
	GPR37L1	LOC647012	TNFRSF9
	PDGFRL	MSX2P1	MYBPHL
	NFE2	TNNC1	C19orf51
	SOCS3	XLOC_006240	IER5
	TMEM229A	XLOC_002872	PLCD1
	DHDH	XLOC_l2_001826	ACSBG1
	GJA3	IL1RAPL2	HSPA2
	LOC643723	EVI5L	XLOC_l2_008667
	OR2C3	UBTD1	METTL20
	RTN2	DUSP5P	GNG3
	XLOC_003729	C3orf20	MIOX
	MAP1A	TSC22D3	MITF
	LINC00051	GPR146	XLOC_l2_012071
	CILP2	SMPD1	TMCO2
	KRTAP1-3	PIEZO2	LOC340335
	XLOC_009810	LOC100505576	LOC100505566
	NKX2-1-AS1	NDRG4	OR4C46
	OSBPL6	CCDC164	C10orf129
	OR4N2	TIMP2	LOC400680
	TUBB3	LIPI	XLOC_006721
	RDH12	XLOC_l2_005690	XLOC_l2_010226
	SHANK3	JAK3	RELB
	XLOC_013960	LRFN1	LOC100505576
	XLOC_011513	XLOC_009782	XLOC_002356
	XLOC_009682	GOLGA6L6	POU3F3
	PEG3	XLOC_014336	SLC2A5
	HIPK4	LOC400927	RNF24
	XLOC_010924	KIAA1467	LOC100510007
	XLOC_009880	LOC388242	XLOC_014349
	XLOC_007971	AIM2	MCART6
	SH2B2	MSX1	XLOC_011278
	NPW	RASA4	ABCG1
	PPP4R4	PCK1	VKORC1
	XLOC_003130	VWA5B2	LINC00312
	SPINK2	ASAP3	XLOC_l2_004631
	PLA2G2F	GRK4	XLOC_013535
	XLOC_010434	MEF2C	SAMD14
	OTUD7A	SORBS1	OSTBETA
	XLOC_l2_001037	XLOC_l2_000804	HSD17B3
	PLXDC1	LOC100131581	IQCJ-SCHIP1
	XLOC_010542	MFNG	XLOC_009269
	ACSBG1	LOC100130157	C1orf170

	XLOC_l2_015239	CDKN1C	MAPRE3
	ITIH4	C20orf160	MAP3K14
	UCN2	S1PR2	CDR2L
	LOC440970	PDGFRL	CCDC89
	CDKN1C	KRT80	BCL3
	GNAZ	LOC100652843	POU5F2
	CCDC89	LMCD1	SGCE
	CYP2C19	XLOC_014219	LOC100652843
	LOC388588	ABTB2	GCGR
	XLOC_008833	XLOC_005910	DLX1
	XLOC_009094	RPS6KA2	XLOC_002840
	XLOC_002650	CEACAM22P	XLOC_008244
	CSRP2	XLOC_013549	XLOC_014333
	BAI2	CRYGB	RHEBL1
	LDHD	XLOC_008729	LOC100507308
	LOC100506777	FNDC4	SLC29A4
	OR2B6	XLOC_006150	GRASP
	CYP2G1P	XLOC_003291	PRKCG
	PAR1	SYNGR3	DKFZP434K028
	GPSM1	XLOC_l2_006944	ATAD3C
	ADORA2A	SIRPB1	LOC100128651
	SESN3	AASS	LOC646999
	PLAC9	RAB36	XLOC_l2_012942
	SOX2	LOC441268	HMSD
	ARL4D	PLIN4	LOC100288911
	WDR96	BAIAP2	CYR61
	IL17RD	TLR5	C14orf49
	XLOC_004256	ABHD8	XLOC_l2_014219
	LOC100653007	SEMA4D	FOXJ1
	LOC283403	ZSWIM6	XLOC_l2_007070
	XLOC_006779	LOC388588	CCDC164
	FBXL16	XLOC_003210	BCORL1
	CNN1	SLC29A4	XLOC_010634
	TSHZ2	CNNM4	LOC100506384
	CD48	XLOC_001852	SPDYA
	FCHO1	XLOC_011284	CCR7
	LINC00319	IL11RA	HPD
	OR56A5	EGR2	XLOC_007189
	CCL20	XLOC_l2_012847	RYR2
	XLOC_001497	DKFZp451A211	XLOC_013559
	C12orf74	LINC00163	PRDM1
	MFAP3L	XLOC_002223	LOC731779
	XLOC_005101	SOAT1	XLOC_010891
	CCDC164	POU2F2	XLOC_006933
	FBXO44	XLOC_012288	DBP
	PMEL	SLC4A11	XLOC_002872
	XLOC_009458	PNOC	LOC100131355
	DFNB31	C8orf60	ABLIM2

	LEMD1	AMICA1	LOC100507109
	TRPV6	GATSL3	SH2B3
	NKD2	RNASE1	SOX2
	XLOC_014105	CRYM	EGFL7
	XLOC_002588	C4orf47	FLJ36848
	FAM182A	XLOC_002650	XLOC_001713
	XLOC_006043	CROCCP3	EFEMP2
	LMTK3	PLA2G4D	ERO1LB
	MEOX2	RFPL2	XLOC_l2_015239
	GNG8	LOC100288911	XLOC_l2_011309
	RRAD	XLOC_000614	NFKB2
	BAIAP3	FOS	ZNF345
	DKFZP434L187	LOC440993	DHDH
	XLOC_011319	XLOC_002515	PURG
	SLC25A42	CARD14	FLT3LG
	ADRA2B	RORA	RHBG
	LINC00472	COMP	MAMSTR
	PDE2A	SEC14L4	XLOC_010279
	NUP210L	XLOC_011559	XLOC_003910
	CCR10	HPX	ARHGAP30
	KIAA1751	TRIM61	MSX2P1
	XLOC_002612	SPTB	LOC100653515
	MANSC4	RBMY2FP	PCK1
	SNORD114-23	MITF	LOC283403
	DYRK3	TTC25	HIPK4
	ABLIM2	LOC285147	NACAD
	GAMT	UCN2	XLOC_001286
	GSTM2	TBC1D10C	SECTM1
	XLOC_007676	XLOC_006918	LOC100288432
	XLOC_010084	PPP2R5B	RNU11
	XLOC_013743	FLYWCH1	DUSP5P
	DLX2	CDKN2B	ARAP3
	XLOC_008251	MUC21	XLOC_004557
	XLOC_l2_012605	LOC339442	MAN1C1
	CALB1	LOC100507959	EXOC3L4
	FHAD1	SERPINF1	DUSP1
	OGDHL	SLC30A2	FBXL16
	PRSS41	LOC100652728	XLOC_011722
	LOC100507959	PGR	XLOC_000980
	XLOC_009100	LOC100506688	SYNGR3
	SLC45A1	CITED4	TMEM229A
	DPF1	BATF3	MFAP3L
	PADI3	C9orf171	SCN3B
	ENPEP	RASIP1	GPR146
	LOC157562	KIF6	XLOC_009196
	REEP2	ARID3B	MPP1
	C20orf173	XLOC_013559	SPTSSB
	XLOC_001345	FLJ26086	LOC388588

	SCN2A	FBXO44	LRRC3
	XLOC_011359	DIO1	C17orf98
	CPT1C	FBP2	XLOC_001816
	LOC100506837	PHACTR3	XLOC_001523
	XLOC_009258	FBXO27	LRP4
	ZNF204P	JAKMIP2	LOC151657
	PTPRU	XLOC_013583	PTPRU
	PTGDS	PIM1	PLEKHF1
	XLOC_005935	GGT8P	SH2D5
	HEG1	XLOC_002296	XLOC_001512
	XLOC_013711	EIF5A2	PIEZO2
	XLOC_012169	LOC100508950	GATSL3
	LOC100132354	C1orf201	EPOR
	XLOC_005763	PTGS1	OPRK1
	LRRC3	LPHN1	GPX6
	BRSK2	LOC284412	ZNF114
	P2RX7	DENND2C	ZNF204P
	LOC100131366	XLOC_003937	FAM46C
	HSPA12A	TUFT1	LOC606724
	COL7A1	LRRC7	RCOR2
	SLC27A1	ST3GAL5	ASB15
	ABAT	SPOCK2	RDH12
	SECTM1	XLOC_002485	HIST1H1T
	LOC439950	GNAZ	ALDH8A1
	XLOC_l2_013427	PREX1	NDRG4
	XLOC_003176	KANK3	FUT7
	ZNF883	AKT3	LOC284939
	CETN1	IL21R	HEG1
	KIRREL2	TDRD1	SSC5D
	XLOC_005535	FBXL16	CXorf22
	ZNF114	CFD	DYRK3
	TNFRSF10C	RAB6B	LOC728228
	XLOC_009797	SNORD114-6	BEST1
	PACSIN3	PPP1R14C	XLOC_008959
	ASPHD2	PCYOX1L	BBC3
	CD7	PTGES	XLOC_010148
	POLN	PTGIR	PPP2R5B
	LOC100508950	CHKA	GPR155
	SYNPR	LOC100287415	SPIB
	LOC100507930	XLOC_011178	IL32
	UAP1L1	CDR2L	SCARA5
	TNFRSF1B	KRT16P2	XLOC_010057
	LOC646999	DDIT4	XLOC_l2_012847
	CLEC4E	GPR111	ADORA2A
	XLOC_010989	ELFN2	XLOC_009706
	CALHM2	LINC00485	XLOC_000101
	XLOC_005621	ABCB9	DFNB31
	XLOC_010293	SCHIP1	PEAR1

	GPR20	NAP1L5	LOC283404
	C1orf130	LOC100131492	XLOC_011359
	WDR66	LOC100507278	XLOC_004120
	XLOC_012343	SLC1A4	XLOC_011513
	SIT1	GRAMD1A	ST3GAL5
	CLTCL1	METTL20	XLOC_013052
	SEMA7A	IQCJ-SCHIP1	XLOC_006756
	SLC10A7	ENPP5	S1PR2
	KCNQ1OT1	ProSAPiP1	CCDC28B
	CORO1A	CPT1C	MICALCL
	TBXA2R	RCOR2	REEP2
	GNG4	XLOC_l2_008252	XLOC_006951
	FNDC5	sept-03	KLHL29
	LOC100652965	LOC285456	XLOC_l2_007876
	LRP4	ATG9B	CYP2E1
	XLOC_008611	SLC9A1	XLOC_l2_013162
	HAPLN3	HTR2C	COL18A1-AS1
	RGS11	C8orf31	PI4KA
	CYP2C9	ICAM5	LOC729040
	C17orf99	XLOC_l2_003627	SLC45A1
	CES1P1	XLOC_012343	XLOC_009682
	HS6ST3	ARID5A	KLF4
	XLOC_011136	MME	ZNF605
	SERPINB9	CDON	XLOC_010434
	XLOC_009684	XYLT1	SV2B
	LINC00087	LOC100507131	XLOC_008466
	GPR155	LINC00051	ITGA7
	XLOC_l2_009332	OLAH	FBXO27
	TMCC2	TAS1R3	LOC388242
	sept-06	ARHGEF4	LOC100506068
	TEX19	DUSP1	XLOC_l2_002709
	LOC253962	XLOC_l2_004631	XLOC_014110
	LOC100131066	SYT2	XLOC_l2_001652
	LOC284570	LOC100652839	C3
	TNFRSF18	LOC100506030	SDC4
	KRT12	PTAFR	LOC100287651
	OTOR	MED14	XLOC_007850
	SNAI3	XLOC_007218	PDZD7
	ETNK2	XLOC_l2_011921	FGF16
	SRCIN1	FLJ40453	XLOC_001698
	LYPD5	LOC152586	FNDC4
	TMEM151B	CCDC89	LILRA6
	RASGRP2	APOE	XLOC_014336
	STAB1	EXT1	XLOC_l2_009140
	LOC100507039	RBM44	XLOC_l2_010880
	SMARCD3	XLOC_003199	LOC100505869
	SCN4B	MYZAP	SLC6A13
	RAB3IL1	DBP	DBNL

	RHOV	SH3PXD2B	XLOC_l2_007204
	SIRT4	TSHZ2	CILP2
	CAMK2N2	XLOC_003508	ACTA1
	ZCCHC24	SNTA1	POFUT1
	XLOC_l2_011302	MGC23284	XLOC_005927
	SCAMP5	CYP2U1	XLOC_013356
	XLOC_004978	C17orf99	KIAA1683
	GP1BB	RFPL3-AS1	XLOC_l2_012984
	SPEF1	COLEC11	OGDHL
	NEURL3	EGFL7	SELM
	LOC100507162	XLOC_009252	XLOC_l2_011508
	OLFML2A	XLOC_007191	LOC200609
	XLOC_l2_007070	ASB2	XLOC_011006
	NOD2	HIC1	LOC100652839
	XLOC_007598	XLOC_l2_014191	CYTH3
	XLOC_011348	LOC100508287	C8orf60
	XLOC_l2_000735	CCR10	OR4Q3
	SNAI1	XLOC_000446	FXYD4
	POU4F1	XLOC_l2_015673	SCHIP1
	CCDC151	SLC25A34	C20orf79
	CTAG1A	FTCD	XLOC_003838
	XLOC_008370	BIRC3	IL11
	ELOVL3	CNN1	PTGDS
	XLOC_013939	TNF	TBC1D10C
	GATA5	GPC6	LOC100506348
	WDR69	LOC401134	CCK
	ESPNL	FOXD3	OLFML2A
	IL5	LEUTX	XLOC_001624
	DUSP27	AKAP2	XLOC_005434
	TMEM169	CLGN	HES4
	RNF112	XLOC_011348	XLOC_l2_006578
	EDN2	CILP2	XLOC_010240
	SGCE	CPEB4	C20orf195
	GEM	LOC254896	XLOC_002650
	LOC100505651	KCNJ5	TMC8
	LOC440330	XLOC_002079	CNN1
	XLOC_009622	PGF	WDR69
	USP2	STRC	XLOC_002821
	ANKRD35	KCNJ18	FLJ40852
	ANKRD34A	LOC728228	TUBB3
	EFNA3	XLOC_002514	EBF3
	CHST6	FAM46C	XLOC_l2_003674
	PRSS50	XLOC_002821	TTTY15
	FBP2	GPR37L1	VSTM2A
	SERTM1	SYT11	CPT1C
	SCN3B	XLOC_001485	AHNAK2
	PP14571	TMEM200B	C14orf23
	XLOC_008731	CTAG1A	ITIH4

	XLOC_010743	NRG2	XLOC_003541
	SYT12	PEG10	XLOC_010989
	GPRC5B	TMEM158	GATA5
	SUSD3	GNG8	XLOC_l2_000297
	LOC100507002	XLOC_009880	LOC100128185
	TMOD2	CALCR	ETNK2
	STOM	XLOC_011359	XLOC_008540
	SLC6A12	LOC100506153	LINC00461
	VASN	KRT83	RNU4ATAC
	PEG10	TPST1	SH2B2
	XLOC_006495	XLOC_007598	XLOC_l2_009332
	APC2	STMN4	CDKN2B
	SLC19A3	MAPRE3	WISP1
	IL1B	HBEGF	LYPD5
	XLOC_l2_000116	XLOC_l2_000018	LOC254896
	NRTN	XLOC_006951	XLOC_010829
	IFITM10	XLOC_010989	EDDM3B
	LOC100288175	CD7	P2RX7
	KLK7	FXYD4	GAMT
	XLOC_l2_011908	PDE4A	LOC100506034
	CD19	XLOC_005633	ARID5A
	MLXIPL	XLOC_009024	VAMP1
	LYL1	LOC283404	XLOC_006150
	PRPH	PPP1R14D	XLOC_012209
	GADD45B	XLOC_013535	XLOC_001305
	BMPR1B	PER1	STGC3
	CTRC	CCDC102B	SLC25A42
	XLOC_l2_014217	XLOC_002059	TBXA2R
	ADRB2	CMTM2	XLOC_l2_000233
	ATP1B2	DNAH12	HIST2H2BF
	KIAA0513	LRP4	LMTK3
	LYPD3	TMEM86A	INSM2
	PPFIA4	CACNB4	SAA2
	SCARF2	XLOC_l2_012942	XLOC_l2_007731
	XLOC_l2_009929	BAIAP3	BIK
	VWCE	KIAA1524	APOE
	LRRC10B	TRIM43	IL1A
	PIP5KL1	ABCA3	RNU6ATAC
	XLOC_012622	SHC2	XLOC_013558
	MAP3K12	XLOC_l2_013564	BMF
	HYAL1	GNG4	TIGD3
	CATSPERB	RCAN2	FHAD1
	DLK2	IL17RD	CYP2C9
	VPS37D	XLOC_009147	XLOC_009487
	LOC283674	LOC100505827	ASB2
	SYNPO	LPHN2	XLOC_009887
	XLOC_000254	GP6	GNAZ
	RAB42	XLOC_000695	XLOC_l2_000018

	ANKRD62	TMEM25	ENTPD8
	RASL10A	MFAP3L	SYT1
	LGALS1	DLX3	NFKBIE
	C11orf86	LOC100131355	OR51I1
	C9orf169	LOC400548	CERS1
	HRH4	XLOC_001988	PPM1J
	SYT11	XLOC_008165	BAI2
	TMEM151A	ZNF391	PER1
	SERPINI1	LOC157562	DZIP1
	IL10RA	DUSP10	ARL4C
	ICAM4	LOC100653007	PRRT2
	C17orf72	RTN2	VAV1
	C1QL4	XLOC_005663	LOC157562
	RGL1	TRIM48	KLF9
	JSRP1	FOSB	S1PR4
	TNFSF9	XLOC_006495	ADAMTS4
	NXPH4	HAS3	CTAG1A
	ZMYND10	LOC100289650	XLOC_004423
	XLOC_004244	XLOC_008246	RAB3A
	PLK5	ZNF114	LOC100507930
	LOC643037	ABLIM2	KCNH5
	HES7	DLX1	XLOC_002688
	NR4A3	PLA2G2F	ICAM5
	KLC3	GDPD3	XLOC_010243
	TDRD6	PRDM1	XLOC_012105
	NAT8L	LMTK3	XLOC_010306
	CEACAM22P	XLOC_005782	XLOC_012899
	PLIN5	XLOC_010275	TEX19
	XLOC_006813	XLOC_l2_009449	SLC27A1
	COL1A1	CBX4	XLOC_002865
	XLOC_l2_012552	XLOC_000233	CFD
	CHD5	XLOC_004626	RTN2
	XLOC_l2_002351	TRIM46	XLOC_002232
	WBSCR28	XLOC_012694	CYP2C19
	LRRC25	P2RX7	ZNF404
	NTN5	LOC643406	LRRC10B
	PIK3CD	C6orf81	LOC100131366
	FSCN1	DMBX1	ADM2
	XLOC_000027	ARAP3	PMEL
	HOXC12	GLIPR2	RASGRP2
	FKBP1B	XLOC_002449	XLOC_012169
	FAM46B	CERS1	PPP4R4
	DHH	SEMG2	XLOC_l2_001310
	CBS	DBNL	XLOC_013720
	LOC100506546	ARHGAP30	LOC100508950
	TUBB2B	XLOC_000686	TTN
	ADC	GSTM2	XLOC_001050
	DACT3	LOC340335	LAG3

	ABCG4	LOC339166	FBXO44
	FBXO2	PEAR1	CLEC1A
	TMEM121	CCR7	XLOC_005529
	KREMEN2	XLOC_004565	BDKRB2
	CREB3L3	DACT1	XLOC_014226
	ANGPTL4	XLOC_009864	LGALS7
	CEND1	XLOC_003709	LOC728175
	SLC2A4	CLEC4F	C20orf194
	SH2D3C	CD19	XLOC_007701
		CALHM3	KIRREL2
		SESN2	ARL4D
		SH2D5	XLOC_009367
		XLOC_l2_010386	WFIKKN1
		RAB42	KRT86
		A2MP1	LINC00087
		ADCY4	XLOC_l2_015766
		BMP6	ZBTB46
		LOC283392	POLN
		SAMD12-AS1	CD7
		ABCG1	XLOC_007349
		XLOC_013039	XLOC_002534
		XLOC_004584	XLOC_008700
		CADM4	DUSP10
		KLK7	ZCCHC24
		HES4	XLOC_l2_015800
		XLOC_003834	LOC650293
		MAP1A	TMEM151B
		DZIP1L	OPRM1
		XLOC_007189	XLOC_000051
		DFNB31	SLC24A2
		XLOC_011529	CYP2G1P
		RHBG	SRCIN1
		RFPL4A	FCHO1
		SH2B2	LY6G6F
		XLOC_l2_011873	LOC100652965
		LOC100507930	BRSK2
		XLOC_012792	CITED4
		SLC5A8	LOC100653007
		ABCC9	XLOC_l2_014697
		BMF	RAB3IL1
		LAMA4	OR14C36
		KIAA1683	PPP1R14D
		WFIKKN1	MOGAT1
		TMEM63C	LOC100505634
		ADORA2A	C3orf32
		RNF24	XLOC_010942
		BMP8A	XLOC_000671
		XLOC_l2_002122	XLOC_007090

		NGFR	XLOC_013754
		XLOC_011938	XLOC_007502
		RBM24	CALB1
		Q29HP5	GNG8
		SPEF1	LOC400927
		LACRT	LEMD1
		CYR61	XLOC_000695
		VAV1	GNG4
		LOC100507039	XLOC_000446
		XLOC_l2_006751	OR5M3
		LRRC6	PLA2G2F
		INSM2	LOC100132354
		GARNL3	XLOC_002643
		SELM	SIRT4
		TEX19	TRPV6
		OGDHL	GPSM1
		ATF3	XLOC_005372
		XLOC_001659	PADI3
		UNC5B	LOC100508631
		XLOC_006933	XLOC_001974
		TOX2	XLOC_003562
		SIRT4	CLDN6
		RRAD	CAMK2N2
		XLOC_003688	EEF1DP3
		GPR155	XLOC_008388
		RGS11	GALNT13
		PPP4R4	ASPHD2
		XLOC_l2_000735	CCR10
		PADI3	ST6GALNAC3
		NACAD	LILRB2
		XLOC_l2_002351	LINC00319
		MICALCL	CSRP2
		GREB1L	PTGER3
		MAP1LC3A	LOC100653080
		LOC100506791	RGS11
		GPR101	IPW
		COL7A1	XLOC_006228
		TBXA2R	NFKBIA
		C17orf72	XLOC_003475
		ZCCHC24	XLOC_009147
		XLOC_014402	TSHZ2
		CCDC151	PGF
		PPM1J	SLC9B1
		LINC00472	LOC643962
		ARL4C	OR1L4
		ADCY9	GP1BB
		XLOC_l2_012552	TMOD2
		PXK	MAP1LC3A

		BAI2	LOC283547
		XLOC_l2_015828	AJAP1
		TUBB3	XLOC_004765
		KCNQ1OT1	XLOC_000181
		TDRD6	CLTCL1
		BARX1	PPP1R14A
		CRTAM	DUSP5
		SRGN	LOC100507065
		XLOC_l2_009285	XLOC_012866
		XLOC_l2_013099	XLOC_001045
		SHANK3	FOXD3
		CYTH3	TMEM169
		SNPH	XLOC_004961
		LOC284570	PPFIA4
		ITIH4	PACSIN3
		DUSP5	SYT11
		XLOC_l2_013456	USP2
		LOC100507162	TNFRSF10C
		FAM9B	NKD2
		XLOC_003338	TMCC2
		AGPAT4-IT1	NPW
		LOC100506731	TRIM46
		LRTOMT	XLOC_014050
		LYST	LPAL2
		PDE2A	PBX4
		MIOX	SCN4B
		HS3ST4	FSTL3
		FCHO1	XLOC_012991
		ZNF582	XLOC_002612
		C14orf49	XLOC_l2_001826
		SRRM3	SCAMP5
		OVCH1	TNFAIP2
		XLOC_011470	RNF112
		LGALS14	XLOC_l2_003419
		ZNF204P	BAIAP3
		KLHL29	SLC22A8
		SNORD115-27	DPF1
		IQCJ	LRRC43
		TMEM212	XLOC_l2_002171
		LOC150185	LOC285547
		XLOC_l2_004318	XLOC_l2_010815
		XLOC_l2_015220	XLOC_l2_001289
		HBA2	FAM182A
		NEURL3	XLOC_004429
		GJA3	XLOC_012343
		TNFRSF10C	XLOC_l2_000116
		TRPV6	XLOC_011173
		XLOC_l2_009332	LOC440330

		XLOC_l2_006003<br>LOC100507228<br>XLOC_002250<br>INMT<br>DYRK3<br>XLOC_007188<br>MYBPHL<br>CAMK2N2<br>LOC100505851<br>CTRC<br>BRSK2<br>WISP2<br>DSG3<br>GATA5<br>ESPNL<br>XLOC_005514<br>CDH15<br>SLC6A3<br>TAG<br>RAB3A<br>LOC100506022<br>XLOC_012169<br>PBX4<br>RNF112<br>ABAT<br>KLF9<br>C1orf130<br>ETNK2<br>ZBTB46<br>LOC283403<br>SCN3B<br>XLOC_008611<br>CYCSP52<br>XLOC_005774<br>PP14571<br>XLOC_l2_010549<br>HAPLN3<br>RASGRP2<br>CATSPERB<br>LINC00087<br>XLOC_012114<br>TAGAP<br>KRT12<br>NPW<br>CSRP2<br>LOC728218<br>SH2B3<br>LOC400541	SPEF1<br>FGF20<br>GTSF1L<br>SMARCD3<br>HSPA12A<br>CCR3<br>FCAR<br>XLOC_007182<br>FAM43A<br>DLX3<br>HIST1H4G<br>OR6B2<br>XLOC_003829<br>C1orf130<br>LOC389199<br>LOC100507039<br>XLOC_l2_011728<br>sept-06<br>PEG10<br>SLC6A12<br>LOC100507002<br>GPRC5B<br>XLOC_003825<br>XLOC_007598<br>XLOC_003834<br>XLOC_009429<br>XLOC_009167<br>XLOC_l2_007586<br>MSX1<br>SERPINI1<br>XLOC_011348<br>XLOC_009813<br>ANKRD35<br>SRPX<br>KRT12<br>LOC100507162<br>Q81CU5<br>PP14571<br>XLOC_005088<br>XLOC_003544<br>C17orf72<br>CD19<br>NCRNA00185<br>OR5P2<br>XLOC_001354<br>XLOC_005047<br>CLEC4G<br>HAPLN3

		ADM2	CHST6
		GPR26	HIST1H4A
		IL9	ELOVL3
		XLOC_014104	TNFRSF18
		LOC100128905	SLC19A3
		CERCAM	GFAP
		PACSIN3	XLOC_014188
		ARC	XLOC_001826
		C20orf103	ANKRD34A
		SLC38A4	ADRA2B
		XLOC_009167	SOX14
		FLJ32756	XLOC_012665
		PPFIA4	EFNA3
		VWCE	XLOC_l2_014757
		LOC100505890	XLOC_002560
		DAO	FOSB
		LRRC10B	XLOC_013614
		LOC100505869	SERPINB9
		FGF21	BARHL2
		KRT42P	XLOC_010897
		CXCL14	SUSD3
		OLFML2A	CYS1
		XLOC_l2_014245	LOC283674
		STOM	XLOC_014410
		SERPINE1	KRTAP4-9
		C10orf113	XLOC_002252
		IL11	SYT12
		MPP1	LOC100506835
		ICAM4	UAP1L1
		ADRA2B	XLOC_001989
		CCR1	NCKAP5
		TRPM6	XLOC_005034
		ATP1B2	CDKN1C
		ANKRD34A	DSC3
		C13orf35	IL10RA
		SLC25A42	XLOC_001504
		CYP26C1	PDE2A
		GNG2	EML1
		DNM1P46	XLOC_005808
		XLOC_010243	GPR20
		RND1	CDKN1A
		HSPA12A	PRPH
		OR5M3	SNAI3
		XLOC_008766	GNG2
		GPRC5B	CXCL6
		XLOC_003547	XLOC_012592
		DDIT3	LOC100132147
		WDR69	XLOC_l2_010947

		KRT86	XLOC_005774
		IL1A	ESPNL
		XLOC_012840	SEMA7A
		XLOC_002841	XLOC_009684
		XLOC_014226	SCARF2
		LOC100506662	PPP1R27
		SPINK2	FLJ37035
		SPAG11B	HDAC9
		HS3ST6	LOC100506397
		sept-06	C7orf58
		LOC100505679	XLOC_004238
		XLOC_012904	DPF3
		XLOC_009601	CATSPERB
		SNORD64	XLOC_012727
		WNT5B	CCDC151
		FHAD1	C1QL4
		XLOC_l2_004556	XLOC_000424
		RDH12	XLOC_000889
		SNAI3	CACNA1B
		XLOC_010307	NFATC1
		MUC19	SRRM3
		SCARF2	XLOC_006664
		SYT12	CCR1
		XLOC_002643	NTNG1
		LOC645434	CORO1A
		ARL4D	XLOC_l2_009492
		XLOC_010556	KCNQ1OT1
		TNFRSF18	PDE7B
		C14orf183	MAGI2-IT1
		LOC100507429	ARMC4
		DPF1	CSF3
		XLOC_l2_011908	UCN2
		XLOC_l2_000116	RND1
		LRTM1	ABAT
		C3orf32	XLOC_l2_000735
		CHD5	LOC116437
		CLTCL1	YSK4
		GRIA2	LOC643037
		CRH	XLOC_l2_015121
		FAM43A	MAB21L1
		OR4C12	CAGE1
		AHNAK2	LYL1
		SLC22A24	POMC
		XLOC_006326	LOC100287036
		DLX2	XLOC_013448
		PTPRU	LOC100508227
		NLRP8	XLOC_010031
		RHOV	XLOC_005465

		SUSD3	STOM
		CYP27C1	MARCO
		LYL1	IGSF5
		LINC00319	XLOC_002859
		ANKRD35	APC2
		LINC00310	GABRA6
		RGS6	GPR37L1
		CORO1A	XLOC_009361
		XLOC_l2_011218	PHOX2A
		XLOC_008260	XLOC_004490
		TMCC2	XLOC_008611
		OSBPL6	KLK7
		XLOC_l2_000514	MLXIPL
		DLK2	CSPG4P1Y
		XLOC_008208	C17orf99
		KRT14	XLOC_004044
		LOC283674	ABCA8
		REEP2	LOC647107
		CYTH4	XLOC_l2_011656
		FBN2	EDN2
		LYPD5	XLOC_l2_000941
		XLOC_l2_008991	LOC100287704
		PIP5KL1	XLOC_003820
		TMOD2	NXPH4
		XLOC_007437	MAT1A
		LOC153910	XLOC_008535
		RASL10A	XLOC_008832
		UAP1L1	TNFRSF4
		EFNA3	KIAA0513
		HCRTR1	MATN4
		APC2	VPS37D
		GP1BB	COL7A1
		XLOC_l2_009363	NRTN
		SLC45A1	PDE1C
		FP588	XLOC_006495
		XLOC_006106	DLX2
		TFCP2L1	ARC
		RGL1	XLOC_010743
		CLDN10	IFITM10
		LOC100129125	ATP1B2
		MYH8	PIP5KL1
		PRPH	GADD45B
		VASN	DLK2
		XLOC_l2_015039	OR1F1
		DHDH	NAT8L
		NRTN	CR2
		XLOC_004761	ZNF582
		C11orf86	XLOC_004073

		SERPINB9	XLOC_007407
		XLOC_l2_006021	LOC284570
		CTRB1	MAP3K12
		XLOC_000403	XLOC_004264
		XLOC_008693	HES7
		TMEM151A	VASN
		ZMYND10	ABCC9
		LOC100129662	RAB42
		FAM75D3	XLOC_008600
		CHST6	TMEM151A
		SLC2A7	LTBP2
		XLOC_l2_007452	ADD2
		USP2	XLOC_001070
		TTC23L	LOC100288175
		TMEM169	HYAL1
		GEM	XLOC_l2_012552
		SOCS3	CXCL3
		HYAL1	XLOC_l2_001548
		XLOC_013240	CTRC
		FKBP1B	SYNPO
		SCAMP5	ADRB2
		ELOVL3	GEM
		KRT17	CEACAM22P
		GADD45B	LOC100288568
		NAT8L	MICB
		NFE2L2	ICAM1
		LOC339568	XLOC_009473
		SERPINI1	LOC283501
		ULBP1	XRCC5
		SLC19A3	XLOC_008358
		LOC100507002	LOC100128787
		XLOC_005667	KLF7
		RAB3IL1	LOC100506853
		XLOC_014412	RHOV
		IFITM10	TDRD6
		XLOC_003882	XLOC_004244
		XLOC_000971	LOC152274
		C12orf69	SOCS3
		CDKN1A	RD3
		XLOC_l2_010458	ZMYND10
		XLOC_006870	CPEB1
		SYNPO	XLOC_000492
		XLOC_010934	FKBP1B
		LGALS1	P4HA3
		FBXO2	XLOC_000950
		ASPHD2	LGALS1
		FREM3	VWCE
		LOC283481	CNRIP1

		XLOC_l2_013859	C6orf168
		XLOC_l2_010855	PLK5
		MAP3K12	LRRC9
		OR8I2	SAMSN1
		XLOC_005791	RASL10A
		LOC100288175	KRT5
		SLC3A1	SNAI1
		CCK	XLOC_l2_011908
		C9orf169	CD28
		XLOC_004397	IL4I1
		HEG1	NR4A3
		PRSS33	WBSCR28
		XLOC_006890	ZBTB8B
		XLOC_l2_015520	SPINLW1-WFDC6
		XLOC_012048	RGL1
		SRCIN1	XLOC_001964
		LYPD3	LOC100508177
		XLOC_008601	COL1A1
		MGC27382	NTN5
		GPR20	FGF5
		LOC375295	XLOC_007000
		EDN2	LYPD3
		MLXIPL	XLOC_010254
		TSPY2	IL2RB
		VPS37D	LOC728763
		XLOC_l2_010601	BIRC3
		HES7	KLC3
		CD101	LRRC25
		LOC100128098	TNFSF9
		TMEM200A	SCGB2A2
		LINC00486	DLX6
		XLOC_004244	HLA-DQA1
		XLOC_005643	XLOC_000027
		KIAA0513	XLOC_007966
		XLOC_006580	C11orf86
		XLOC_l2_010835	TNFAIP3
		C1orf61	PLIN5
		SIAH3	FAM46B
		XLOC_l2_004385	XLOC_l2_002351
		BDKRB2	CBS
		XLOC_005915	C9orf169
		XLOC_l2_015322	NEURL3
		TNFSF9	FSCN1
		XLOC_l2_006013	HOXC12
		XLOC_010148	DHH
		HOXC12	ABCG4
		XLOC_002707	XLOC_004693
		SMARCD3	ADC

		NXPH4<br>XLOC_001881<br>DHH<br>LOC339400<br>SCN4B<br>SNAI1<br>XLOC_005808<br>XLOC_005454<br>IL1B<br>GPR141<br>XLOC_014220<br>NTN5<br>PLIN5<br>CHAC1<br>WBSCR28<br>LRRC25<br>XLOC_l2_013741<br>BEST1<br>XLOC_014001<br>TUBB2B<br>XLOC_006213<br>LOC285626<br>XLOC_005008<br>MICB<br>LOC643037<br>FAM46B<br>C1QL4<br>SLC35F1<br>JSRP1<br>KLC3<br>CBS<br>PLK5<br>LOC731779<br>NR4A3<br>TMEM121<br>FSCN1<br>LOC100506546<br>XLOC_000027<br>KREMEN2<br>ADRB2<br>COL1A1<br>IL10RA<br>ADC<br>ABCG4<br>SLC2A4<br>SEMA7A<br>DACT3<br>CEND1	TUBB2B<br>IL1B<br>CXCL2<br>JSRP1<br>DACT3<br>LOC100506546<br>ICAM4<br>IL17C<br>RRAD<br>FBXO2<br>CXCL1<br>SLC2A4<br>TMEM121<br>KREMEN2<br>CREB3L3<br>ANGPTL4<br>CEND1<br>TNF<br>SH2D3C<br>CCL20	

		ANGPTL4<br>CREB3L3<br>CYP1A1<br>SH2D3C	

TRAIL+SN (n=3313)
ZNF75A
RRP9
NBPF10
SMAP2
MAD1L1
IL10RB
SPDYE2
SNHG5
MEF2D
TGFBI
STRN
TCP1
SNORD71
KIF13B
SLC3A2
DBN1
LOC100506720
XLOC_008781
TRAFD1
ZNF295
HOXB13
FBXW7
FAM69A
EPN2
ZCCHC11
ATP2A1
FAM179B
PVT1
KIAA1609
XLOC_008015
PNMA1
XPR1
LOC399900
AQP7P3
DYNLT1
HAUS6
SOX13
GSTT2
C9orf116
KIAA0895L
PI4KA
TNNC2
STK11
GALK1

KLC2
ACSL3
XLOC_l2_006404
LOC285141
XLOC_013181
ARGFXP2
CHD4
MC1R
DNAJB2
NEK1
MAPK8
ZMIZ1
CXorf40B
SCARNA14
CEP68
C1orf55
NEDD4L
XLOC_013914
LOC100507672
SEPP1
DDHD2
XLOC_006124
TMEM104
SAR1A
FAM91A1
TCFL5
CNST
NT5C2
RPL28
XLOC_l2_008140
RAB43
ANKRD13C
XLOC_l2_010097
FBXO3
DHX16
CHRM4
ARHGAP33
XLOC_000166
USP20
PER2
UBE2I
ARPC4-TTLL3
ZBTB47
CRABP2
PLIN2
INPP5F
FBXL20
CCDC165

SLC26A6
LAMB1
LOC100506127
XLOC_l2_004640
FA2H
SLC25A28
SLC6A8
SEC31B
ACOX1
SEC14L2
PTPRE
OBSCN
CAMSAP1
OR13A1
ACBD7
TM2D2
PRKCZ
KLK5
FAM207A
TCF20
C12orf51
MIPOL1
SNORA42
GZF1
GGT3P
ARHGAP17
IPO9
LOC100287813
MGEA5
EFNA5
KIAA0284
LAMB2P1
GRB10
TNFRSF6B
XLOC_012754
C6orf1
TLE3
TMEM185A
CDH1
LOC100509105
C1orf124
C17orf51
SNORD83A
CDC34
XIST
ARFGAP3
XLOC_l2_004371
HSF2

PELI2
LOC100130876
PSMB9
ST14
SNORA73A
TBC1D9
C19orf59
FUT8
CD6
GPR137B
MFAP2
SLC29A1
PLCB2
EPS15L1
XLOC_011837
RBP5
PDXK
LOC100505933
XLOC_006321
ETV5
SYTL1
PPP1R18
LOC100506394
VAC14
CPLX1
XLOC_000495
SNORA81
RAB33A
FBXO36
SNORA80
PDK2
SNX27
LOC100131826
PTPRCAP
XLOC_005041
PIP5K1B
RNF149
SMG1
HDAC3
WNK2
SPSB3
ABHD3
TRIM62
SUSD2
SNORD101
ZNFX1
UBE2H
SNORA80B

F11R
BAHD1
STXBP1
RNF157
XLOC_l2_015037
XLOC_014388
STAT3
WDR83
XLOC_012162
POU3F1
MAPK15
SPIB
ABCD1
B4GALT1
LRRC1
SNORA23
NAMPT
DFFA
COQ10A
CD82
KCNK5
FADS3
TBX10
PLEKHG2
SNORD74
LOC100505815
KIF16B
XLOC_l2_006745
SNX29
ENPP4
C2orf54
XLOC_011448
RNU105A
EPHB3
SCIN
HYAL3
PLA2G4C
RALGPS1
IFI30
C1R
MAP3K11
GLTPD1
TNFAIP1
PMM1
TMEM2
LOC100506852
ZNF707
SYNJ1

LOC100132495
RBBP5
RIMKLA
SLC25A36
XLOC_006037
OCR1
SHISA7
XLOC_l2_005438
CGB
C20orf106
XLOC_014237
GDI1
C1orf63
USP37
IL15
SLMAP
GTF2B
FBXO46
MAP3K9
NUPL1
XLOC_009485
NRARP
XLOC_l2_000010
SMG7
CDC42EP2
CNTNAP1
FLJ30403
SPTAN1
TMEM136
XLOC_011223
GOLGA7B
KIAA0226
TOP1
POLR2J4
BIN1
XLOC_010376
LIPG
DNASE1L2
FAM90A10
BICD2
LCN15
LOC400958
CA4
FRY-AS1
LOC100652730
MXRA7
NME7
ZFAND3

PLXND1
RILPL1
CLIC3
NPC1L1
SLC6A6
UNC13D
TSL
B3GNT4
ADIPOR2
DNAJC12
KCTD5
LOC100287803
C2CD4C
F2RL1
SMTN
PBXIP1
NQO2
LOC100505904
CCDC130
XLOC_l2_000416
RAP1GAP
FRMD8
XLOC_l2_006026
GRIPAP1
SH2D6
HERPUD1
PIM2
PIBF1
HOTAIRM1
THSD4
GPT2
C19orf26
LCA5L
GNMT
USP33
XLOC_012192
SCARNA9L
USP32
LAMC2
LOC202025
DGKZ
LPCAT3
XLOC_l2_015034
WDR66
XLOC_000683
XLOC_000048
C3orf52
YIF1B

LY6G6C
LOC100507018
LIX1L
TBX3
IL6ST
ZNF789
ARMC2
ND4
SIRT7
LOC100129781
LCK
RAB26
AKIRIN1
WDR67
SH3BP4
GPAT2
XLOC_l2_000399
TRIM11
DGCR5
MBOAT7
BCR
CROCCP2
XLOC_000350
ARHGAP23
TESK1
LOC389791
GLA
ZNF251
UCKL1-AS1
ZNF709
DNAJC6
ULK4
TTC39B
LOC100653120
TBC1D24
XLOC_001624
GPR157
LRRC31
CATSPER2
VWA1
MALAT1
XLOC_007775
PSAT1
FBXO31
SHANK2
DYNC2H1
SNORD87
WDR19

FLJ21369
MKLN1
MICAL3
LUZP1
LOC100129034
SPDYE7P
MED20
KCNK6
SNORD29
PC
RANBP10
ZNF10
BCOR
SNX1
XLOC_008185
SLC46A3
LRP6
KIAA0930
DIP2C
MIR22HG
ANKRD54
DRAP1
SNORD22
ZNF555
DNAJC5
PRKAB2
DENND4C
CGN
FANCB
LEPREL2
STX1A
CERS5
LIPH
ADAM11
RNU4ATAC
C16orf80
IFNAR2
C10orf137
MAPK8IP1
BBOX1
LEPRE1
TJAP1
LOC100506990
TXNRD1
PTPRJ
TSC22D1
FUZ
CCDC157

DGKD
STXBP4
MGAT4A
GPR3
PCYT2
PPP1R13L
MACF1
MYH9
COL20A1
ZDHHC14
FOXK2
SLC30A1
FANK1
XLOC_l2_005781
USP12
SNORD34
ZACN
XLOC_013506
SNORD68
NEAT1
ARHGAP4
MAFK
C1orf52
C1orf81
ACSS2
VWF
PLEKHG1
IL23A
XLOC_014105
TSC2
RALGAPA2
XLOC_012009
KIF1B
NPHP3
XLOC_l2_015209
WDR91
AFAP1-AS1
REEP6
C6orf228
SPTSSA
LOC100505717
WNT7B
ZNF608
LOC100653178
NCK2
UBR2
SNAPC2
KCNAB2

MAP3K3
DNAH6
IQSEC1
FBRSL1
SHH
ABL2
BCL2L12
PTPRN2
SLC1A5
XLOC_l2_005130
CHMP1B
AK1
AP1S3
POFUT2
ARL14
XLOC_l2_009883
VPS13C
AFF4
XLOC_010207
LOC100652766
C17orf28
FAHD2A
VPS18
MAP7D1
RPS6KB1
ZNF79
ANKRD30BL
MAX
XLOC_014243
ZBTB43
STRA6
LINC00341
PRSS30P
C1orf195
ANKRD30BP2
CHD9
ACOT7
HPN
ELL
SNORD67
C15orf26
SGMS2
HDAC5
GGA1
XLOC_l2_008203
CCDC88B
C12orf44
CDH24

XLOC_007413
OSBPL3
DDX26B
C9orf85
CAPN8
XLOC_003481
ZNF77
MIA3
AFF1
TRA2B
EPB41L5
CDC42BPB
PRRT4
CASP10
HES5
ANKFY1
SUFU
SLC2A1
C14orf55
TSIX
TRIP10
C6orf141
DNAL1
TGIF2
MKL1
TBC1D2B
CIDECP
XLOC_009122
LYG1
HSPG2
SNORD125
ZFAND2A
TSPYL2
MYPOP
NPIP
PQLC1
NT5DC3
ZNF654
ACHE
SULT1A4
AGFG2
TSPAN15
MPV17L2
CEL
LOC100499467
DOCK6
ZBTB5
KIAA1462

KIFC2
ZNF224
MYH7B
XLOC_l2_000092
FLCN
GPR137C
XLOC_013994
LOC100505930
KDM4B
TCF7L2
BAGE
TULP3
RASD1
LOC100506428
LINS
SPECC1
SYS1
ATG9B
XLOC_l2_010330
CLIP3
PLEKHG5
SLC25A35
GOLT1A
NINJ1
EPHB6
PHRF1
XLOC_008916
RIPK4
EDA
KCNJ14
OPTN
FNDC3B
UBXN7
GZMM
LOC645638
PRODH
GPR162
DPM2
ZNF474
TAF3
C1orf228
SLC5A11
MAD2L2
MICAL2
XLOC_002577
SCARNA20
VGF
BACE1

CALML5
TP53INP1
SEMA6C
CLCN2
FGFR3
WDR60
SLC38A2
SELPLG
SNORD32A
FAM160A1
MAP3K10
KIAA1432
JDP2
C12orf70
FZD9
OR2AG1
INPP5A
MYO7A
LOC100506183
PKD1
SLC22A5
CD81
FAM167B
ABHD6
ARHGAP29
PPP2R2D
WFS1
OVOL1
AKAP5
TOR1AIP2
LRRC8A
HPCAL1
XLOC_008559
LOC100505633
SOCS4
IER2
PPAP2B
MOB2
ZFAND5
PIKFYVE
TSEN54
PP12719
MED26
BTBD19
CYP3A7
ATP7A
CPT1A
SPATA2L

LINC00340
ERVMER34-1
RASA3
XLOC_004640
SGPP1
SHD
CDC14B
GTPBP2
SERPINA6
FOXD4
FANCM
GGT1
KCTD13
CREB3L2
FAM132B
TTLL5
PRKAG2
PPP1R12B
XLOC_007776
LOC642852
CISH
CRY2
TAP1
OTUD1
SNORA10
PRRG1
ZCCHC14
SSBP3
GAFA3
LLGL2
ZFYVE28
SAMD8
XLOC_000340
ZNF124
LOC100130419
XLOC_007813
HERC1
LOC100129675
CYP39A1
XLOC_013350
PGPEP1
KLF10
TLE1
ZNF815
LOC283624
ARID4A
CYP3A5
LUC7L3

TBC1D22B
ETV3
ATP13A3
CDK18
LOC440910
FLJ32224
XPA
XLOC_009181
ARRB2
XLOC_003738
ZNF778
NR6A1
FAM106CP
LOC100506800
ZNF821
QSOX1
XLOC_005426
PDE4DIP
TMEM92
ADAM20
FAM82A2
METRN
RMRP
ATF4
MLLT4
ZFP36L1
PCBP4
DRD4
MEGF6
ENDOD1
CD14
LMTK2
DIO3
SNX18
LOC440905
SNORD30
TRAM2
PEAK1
XLOC_013364
THBS1
OCLN
XLOC_006263
PECAM1
RLF
C7orf54
CENPT
SLC6A10P
HPSE

GP5
C15orf5
XLOC_l2_013293
GNB1L
C1orf135
FUCA1
GBP1
SNORD3B-1
REL
SLC24A3
C5orf56
LOC254100
ST6GALNAC3
PFKFB3
FAM108A1
SNHG1
CLDN7
HN1
XLOC_l2_015561
CTNND1
MCTP2
XLOC_005737
XLOC_010601
XLOC_008223
SNORD25
SLC9A3R1
LOC100132240
XLOC_l2_001138
DCBLD2
RAB9A
TPMT
PROC
PDLIM5
XLOC_l2_008888
FLJ43315
TSPAN5
SLC12A4
DEPDC7
LOC100507412
EFHC1
FMNL2
TSPAN33
ISYNA1
ZRANB1
FHDC1
SLC31A1
KLK9
CEP170

ADRBK2
ADRA2C
CD59
PIEZO1
RSL1D1
NOSIP
LOC100130899
FAM102B
C7orf43
LAT2
MYO10
RUNDC3A
C9orf140
P2RY1
FAM135A
CHST7
GALNT6
MARCH3
SNORD94
RASSF6
FOXO1
PDZD7
TBC1D10B
C19orf28
S100A1
C21orf88
ARHGEF18
JUNB
CHN2
UPP1
PAPLN
PAPD5
ZSCAN5B
EFCAB3
XLOC_001023
CTSL2
TMEM81
FBP2
LDHD
XLOC_013445
SV2A
LOC728431
NR1D1
PHF20L1
CCDC116
CDX1
ZBTB10
FOSL2

IL18BP
BREA2
XLOC_003881
LMOD2
PAQR5
IL1RAP
RHOD
XLOC_005442
STON2
UBR4
PNPLA7
PPARGC1B
PAFAH1B2
CSNK1E
HSP90AB4P
LILRB5
LOC650293
KIDINS220
SIRPG
TSPAN32
LOC100506245
OSTM1
PLIN1
PCSK7
ZNF385A
C16orf87
ANKRD36B
TMEM145
LOC100289187
SNORD36C
SCML1
KCTD17
RTTN
SCARNA17
CCNE1
ANKRD11
XLOC_l2_014686
SLC22A23
DTX2
DNAJB5
XLOC_003480
HIVEP3
FAM107B
XLOC_001699
AHR
MFSD2A
PFKP
GPR133

CBL
C7orf51
UGCG
ELL2
PTCD3
SGK1
SPATA6
LINC00483
TTLL7
GNAI2
XLOC_l2_006578
XLOC_000794
SNX25
KIAA0895
CCDC126
AK7
EGR4
XLOC_l2_004854
LRIG1
XLOC_004700
S100A14
GDPD1
PPP1R9B
ADORA2B
PVRL4
XLOC_008614
LAMA5
DCUN1D3
XLOC_009684
FER1L4
SNRNP48
PCSK5
RUNX1
CEACAM1
HIP1R
IRGM
TPRA1
SYNJ2
NMUR2
GNA11
H2AFB3
C16orf93
XLOC_008100
HS6ST1
SLC12A7
XLOC_l2_007644
KCNA10
CDC14C

PHLDB3
psiTPTE22
ANKRD36
RPS2
LOC100128670
TMEM229B
RAPH1
LOC100506342
TNFRSF21
CKB
XLOC_014137
STK17B
ZNF519
XLOC_009382
LOC100130193
C13orf16
SAMD4A
LOC100505675
PTGS2
RALGDS
CIB2
LOC284581
VDR
MLF1
XLOC_001230
TTYH3
PIM3
ROM1
SNHG7
ZNF221
KANK2
DIO3OS
CNGA1
SLC27A1
CMTM1
GAS2L1
CDX2
FHL2
CYP26B1
RECQL4
POTED
OTUD7A
AFMID
FGF11
BTAF1
P39194
TBC1D20
SERPINA3

GUCA1B
RGNEF
KIAA1549
RHOBTB3
ORM1
CDA
SLC25A16
KBTBD8
KIAA0907
XLOC_002131
PODXL
GOLGA6L9
GRHL3
TNFRSF10D
CAMK1D
PLCD4
ITGA3
CIDEC
TANC2
TESK2
XLOC_l2_009571
PRKCE
LOC100127885
SNORA21
HCFC2
FLJ31104
RPS24
PPP1R12C
SCARNA4
CD86
ZDHHC3
LOC100652777
PDPK1
SYT15
EIF4A3
RHCG
NOTCH2
CNNM2
LOC100506157
IGSF3
SH3TC1
SLC9A5
CNP
PRSS35
RHPN2
SDHAP1
CALCOCO1
DDHD1

SEC16A
LOC100507305
GIPR
KIAA0913
MAN1B1
HK2
GAFA1
SNORD100
LOC100507364
XLOC_005968
RANBP6
STK17A
MRGPRF
SDCBP2
INPP4A
TMEM189
LOC541471
ACOXL
TMEM190
FAM83G
TNIP1
LOC644662
ABCC1
LOC643650
MIR7-3HG
ATG4B
TNFRSF13C
PGAP1
FGFBP1
ZNF598
BAIAP2L1
HEBP1
SHF
SNORA57
NOTCH2NL
BAI1
XLOC_l2_013001
ND2
XLOC_l2_001064
SLC41A1
ZNF703
TPPP
CTH
KLHL30
LMF2
PIGH
SCG5
LOC100190939

GK
ADCK3
SNX22
SEPX1
FRRS1
GLIS3
DAGLB
LOC143666
C1orf170
LOC283174
LOC648149
SBF1P1
IL28RA
PLAUR
RNF183
SNORA33
EFNA4
LOC283050
ERI1
LRRN4CL
XLOC_007368
CHRD
FLJ23867
PALM3
GPBAR1
LOC100505634
LOC100653030
SLC25A37
PRIC285
RTN4R
GGTLC2
C3orf35
LEAP2
PRR20B
ALDH1A3
SNORD43
SIK1
SNORD84
GABRE
MPRIP
LIN7B
XLOC_011535
FAM70B
LRWD1
LOC401052
SNORD50B
XLOC_009868
FSD1

GJB4
CYFIP2
XLOC_l2_006027
SLC2A8
ZNF833P
GAK
KSR2
XLOC_008487
LBH
LOC100506123
B3GAT1
KCNC4
UBXN11
ACCN3
SCARA3
MYO9B
CCDC75
FARP2
FBXO32
LOC100505664
MIAT
ENGASE
FOXJ1
XLOC_001351
CT45A1
TFAP2E
SPATA13
TMEM45B
XLOC_004122
XLOC_l2_013116
NXN
SLC19A2
XLOC_012065
KIAA1539
MB21D2
ZNF780B
ANKRD13B
LOC401561
LOC644192
NIPAL3
IGFL1
XLOC_l2_014098
SCARNA2
VLDLR
CYP2D6
CSDC2
KIAA0664L3
HEXIM1

SYTL4
RNF114
ICAM2
ADAMTSL4
GLS
ENPP1
DNAJB3
XLOC_011924
ACSF2
LILRA3
SCARNA11
LOC149773
KLHL28
ZNF516
SNORD89
POLR2F
POU3F3
NOTCH1
PLAG1
XLOC_001576
LOC729513
BCL2L11
RNF208
XLOC_l2_011901
PDIA2
CBX6
SARDH
SSC5D
UBAP1L
XLOC_005082
CEP72
SNORA4
LOC387895
MAN1A1
CACNA1G
XLOC_000922
INSR
WNT4
AAK1
CHTF18
TNFRSF11A
XLOC_l2_007802
LOC100288092
TNNI3
RAB11FIP3
MIDN
SPAG9
STK4

FLJ45248
LOC100128001
S100P
ITPR1
TMEM184B
VAT1
PLA2G10
XLOC_002133
STK40
AQP7
ALS2
XLOC_012078
EZR
FOXP1
TSPAN9
PLAT
CD74
GPCPD1
NPAS2
PCSK4
CHST12
NFATC2
CCNL1
PIK3CD
XLOC_l2_007928
SNORA52
XLOC_l2_013873
FLNB
LOC100505882
XLOC_011563
LOC100507594
TCEAL6
XLOC_l2_003295
DNAJB1
APLP1
OR7E91P
ZFPM1
C9orf89
GRIN1
PLCG2
KLK4
RAB3B
XLOC_008809
FKBP5
AGPAT9
LOC143188
MAP2K3
PCOLCE

DAAM1
XLOC_000755
SERP2
SYNGAP1
FAM161B
XLOC_013866
XLOC_l2_002171
LOC388152
XLOC_l2_010568
SNORD48
TRIM4
FAM132A
CECR7
LOC100507580
ARHGEF26
GPR124
XLOC_003522
LOC153577
SCARNA10
BCAM
MAFB
XLOC_014139
KDELC1
ZNF217
FLJ35390
HRASLS2
MAPK11
IGLL1
LOC100507918
VILL
LTB4R
WNT6
C7orf52
MBP
MSGN1
POTEB
LOC100505555
PACSIN2
RPS21
ATP6V0A1
FBXW10
XLOC_002793
PTPN13
C6orf154
SBF1
SEMA6A
GPR153
WNT11

MIB2
CPNE5
XLOC_011872
KCNF1
DOCK9
Q8N7V6
IL6R
TMEM110
TTC7A
WWC3
LOC100190986
LOC100509213
C16orf55
XLOC_l2_005076
GK3P
PPIEL
IL27RA
XLOC_012593
SNORD4B
LOC100131234
XLOC_006335
LOC100128477
TTBK2
XLOC_l2_008151
JARID2
SLCO4A1
TTLL10
GOLGA2
XLOC_l2_015033
SNORA68
LOC100132354
MAST3
LEMD1
TCIRG1
MUC12
COL23A1
PACSIN1
XLOC_l2_003974
PIP5K1C
XLOC_l2_007834
ANKRD2
ZDHHC8
RDH13
SLC25A30
LMF1
LOC100131831
GSTM4
TNXB

RASGEF1A
LOC100131820
LOC728802
SCARNA6
CCDC85C
SPTBN5
NXF1
LOC100131702
LOC100268168
XLOC_000194
ATP2B4
GRK5
ABCB1
LOC100505551
C16orf70
UNC5CL
RPS27
EXOC8
FAM89B
ECE1
ULK1
LOC100129363
INHBE
GAN
PMAIP1
C6orf124
DEGS1
LRFN1
SHB
CDC14A
FAM126A
C17orf67
CCP110
MBNL2
XLOC_004517
FEM1C
LOC100129617
IZUMO4
PLIN3
SOCS7
XLOC_005488
XLOC_012393
CD55
OAS1
XLOC_006505
XLOC_l2_014369
TTLL11
XLOC_003482

ALAD
ISG15
JUN
XLOC_013932
LOC100506641
CLEC16A
SLC25A29
LOC100505668
XLOC_l2_002204
HLA-F
BTN2A3P
GOLGA6L5
PRINS
KHK
SNORD35A
TMEM206
LOC728705
PLAU
TSPAN4
TTL
PGM2L1
FAM53C
MARK4
MFGE8
INSL4
XLOC_011480
SETD3
XLOC_l2_015127
XLOC_l2_009285
TBC1D10A
TRIM3
LRRC16B
SH3D21
PMPCA
GPC1
SNORA53
XLOC_008556
XLOC_l2_004317
PPARGC1A
LOC283070
SLC7A5P1
KIF7
NUDT17
APOC3
LPIN1
C7orf53
CLDN23
ZNF296

FAM55C
SLC25A44
COX6B2
LOC338620
C6orf25
C6orf222
RARA
LOC100505760
LOC202181
CTGF
SLC25A22
XLOC_009526
XLOC_l2_006152
XLOC_010377
XLOC_008619
XLOC_013950
PLGLB1
PPIP5K1
XLOC_l2_008221
RN7SK
MLLT11
RHOXF1
LOC729652
ANXA6
WNT10B
CCRN4L
SRPK3
LOC100506890
NPIPL2
LINC00260
LOC100507165
ATP2C2
PLEKHM1
FLJ35024
PAG1
CDHR5
XLOC_002830
FYN
FAM131A
LOC100653024
GRIN2C
SUN1
LOC728061
XLOC_002085
MYO15B
SNORA44
TLE6
PDHA1

C1orf9
PYGM
NCKAP5L
SNORD83B
ACSBG1
CT45A5
XLOC_l2_011291
TBPL1
XLOC_000302
XLOC_009007
KCTD6
BCL6
TMEM45A
ND5
SQSTM1
MAP4K2
LOC285957
LOC100127886
RHBG
PPL
ZNF324
ENPP2
GGTLC1
MXD1
SPTBN1
SERPINA5
FZD5
SNORD76
XLOC_008439
DISP1
LOC100507563
LOC100505725
ILF2
LOC100287177
XLOC_l2_004611
C20orf160
VAX2
WDR26
PLB1
XLOC_002987
GDF11
AGSK1
C20orf54
XLOC_l2_015038
JMJD7-PLA2G4B
C14orf45
PPFIA1
LOC100505988

XLOC_006336
TBC1D2
SH3BP2
NKD2
ST5
NDOR1
TAGLN
XLOC_014247
OTUD3
SPON2
AGPAT2
LOC390940
MAP1LC3B
LOC100507803
CYHR1
NPC1
RSPH3
XLOC_008466
XLOC_001441
LOC100129399
XLOC_006092
MKNK2
C10orf116
LOC440330
CYP2B6
FMNL1
USH1G
SYT7
SNX30
EHD4
SOX2
HIP1
ARHGAP39
PLEC
POGZ
PHF1
GRIN3B
MEGF9
STAB1
NEK10
ABLIM3
PLLP
BAK1
CYP1B1
RNF223
C14orf37
PKIG
LOC145757

LIMS3L
C10orf54
SLC46A1
NRP2
NHLRC4
KRT16
RNF207
PDXDC2P
NNAT
XLOC_l2_005179
PELI3
FBXO27
CCL3
APBB3
KIAA1984
TMED6
HIVEP2
TMEM120B
KCNH2
SNORA65
GCDH
ARFGAP1
CDC42BPG
SCARNA5
IRF6
XLOC_012912
USP49
RASGEF1B
LIN37
PRSS48
LOC344595
XLOC_002035
CARNS1
ADAP2
CLEC3B
POU2F3
XLOC_014399
ULBP3
LOC100652769
ZBED2
TNFRSF1B
SLC2A5
XLOC_l2_013267
TMEM170B
PAOX
FAM25A
CSGALNACT2
ZFP36

ETS1
CLDN11
SAT1
ICOSLG
PI4K2A
CTSK
LOC643201
MID2
LOC100132247
XLOC_l2_005020
ATP13A2
GAPVD1
LOC442028
LOC100652915
GATS
CHRNA10
POTEE
SNORD31
KLHL5
RFX2
FCHSD1
MYT1
RAB8B
DHRS9
XLOC_002356
FOXD1
ACER2
IFFO2
ZNF556
LOC284219
PRDM2
LOC100506802
PPP1R13B
LOC100507639
MRPL42P5
MAPRE2
SYTL2
XLOC_010881
Q6LRY1
VPS53
GSTM1
XLOC_006513
C4orf21
XLOC_000214
SERPINE2
LOC100509256
ASMT
SLC43A3

LNX1
TRIM50
ST6GALNAC6
SESN3
LOC440300
LOC100509487
AP3B2
PPARA
CATSPERG
SLC9A3
EFNB3
UBAP2
OSBPL7
ZNF711
KREMEN1
LOC400499
PRR5-ARHGAP8
ZBTB48
LOC100507309
TSPAN10
STARD5
ASB6
AFAP1
SLC16A10
KLHL15
LOC100130987
AMH
LOC100128198
GLI2
XLOC_l2_009301
POR
MFSD4
XLOC_010945
XLOC_l2_012678
EXOC3L4
USP17
TAOK2
KIAA1467
CLSTN3
DAPK2
ATP8A1
C17orf76
ALDOC
TTC16
OXTR
UBE2Q2P1
GTPBP3
NR3C1

TBC1D8
ANKRD42
NLRC5
LOC100506047
CCDC64B
C12orf5
XLOC_009576
CLEC18B
LOC100505787
RNU1-5
LOC100294362
DKFZp434J0226
KRTAP5-8
B3GAT3
Q6BWL2
TRIM10
ATG16L2
COL6A1
C10orf114
SPTBN4
LOC100128292
ZC3H12A
PORCN
TTC9B
STARD9
RPS6KA5
CLDN9
LOC338653
C6orf81
LOC652990
TAC3
DISP2
KIAA0247
XLOC_003235
SLC26A1
NDRG1
PPM1N
LOC646999
SIRT2
MEF2B
FAM85A
GIT1
HSPA2
NR2E3
MTSS1L
RPUSD1
VTRNA1-2
LOC650368

AQP3
VCL
LRFN3
NCF1
NTNG2
MAMSTR
ST6GALNAC4
WDR96
XLOC_007262
SLC2A6
KLK8
BACH1
CCL27
XYLT1
LOC100134138
RNGTT
ALDH8A1
AGT
DAPL1
FOSL1
LOC100131366
PLEKHA4
PI4KAP2
SLC31A2
XLOC_009458
SNORA64
AQP10
TMEM236
LOC100130930
KISS1
C1QL1
IL20RB
TMPRSS7
NGEF
PCNXL2
KRT35
PRO0628
STRC
CACNA1A
RAET1K
HEY1
PIDD
LINC00482
TOR1B
JAKMIP3
NINL
NAB2
CABYR

ST3GAL4
AMBRA1
NKIRAS1
FAM118A
AIM1L
ARSK
TNNT1
ANKRD27
NKAIN4
NPHP1
CNPY4
C18orf45
LOC283588
CCDC84
PLEKHM3
GPRIN1
LOC100653017
sept-12
KCNMB4
CD164L2
TRAF3IP1
MMP15
ANKRD33B
FBXL18
FRY
LONRF2
IGFALS
FLJ42022
IDI2-AS1
GCNT1
RAB24
DENND5A
SEC61A2
VTRNA2-1
XLOC_005039
AGPAT4
RNU6ATAC
SERHL2
BCO2
C10orf47
CBFA2T2
FAM40B
VTRNA1-3
XLOC_l2_001947
MAST1
MUC17
TMCC3
RPS6KL1

TRIM41
FGF8
ABCA7
LRRC3
PLCD1
POLH
SNN
FLNA
XLOC_013981
CBR3
TMEM52
DGKA
LOC100506688
TMEM98
HMHA1
LOC440792
PLCXD2
RPL32P3
RSPH4A
AVIL
DNHD1
ITGA10
SYTL3
TSC22D2
SOX8
KIAA0319
CRYGS
PLXDC1
LOC100131774
SNORA17
XLOC_008711
PRKCD
HTR7
LPAR5
TUBB2A
RSU1
TBX19
LPPR2
KIAA1671
RAB30
KIRREL2
SNAPC4
OSTBETA
RIOK3
HES2
XLOC_012688
LOC729668
COL16A1

XLOC_000421
PHYHIP
KIF3A
LOC440993
SERPINF1
HSD3B1
BTBD11
G0S2
ZNF114
LOC399715
TUBGCP6
XLOC_014257
ESAM
LOC100129268
ADA
XLOC_012515
TMEM132A
F3
C2orf48
SCPEP1
CCDC114
TNNT2
XLOC_003734
XLOC_000625
SLC16A3
LINC00222
LOC389199
NANOS3
CCDC153
ISG20
XLOC_013906
REC8
LOC100131726
GLI1
LRRC43
IRF7
LOC100130849
LOC100130547
LOC100507419
GLCE
XLOC_l2_013931
XLOC_007205
XLOC_012021
FAM182B
XLOC_002122
ALAS1
BMP8B
XLOC_l2_015239

UBE2Q2P2
EPHA2
SPDYA
EFR3B
NSAP11
PLXNA4
XLOC_005748
XLOC_003327
CCDC164
XLOC_005341
HIC2
PNPLA2
LOC100652965
XLOC_013100
ADAMTS17
DOC2GP
HOXC13
XLOC_000670
UNC119
ZNF775
AZI1
PITPNM1
CCDC19
MCAM
PADI2
JUND
CNN1
TNK2
TRPC1
SLC35E4
MUC4
XLOC_000011
PRSS8
KIF5A
RHPN1
XLOC_012679
RIIAD1
KLHL26
MPZL3
GOLGA6L10
H1FX
DOK3
CACNG8
PIRT
C9orf174
FAM115C
INPP5J
SLC22A4

SPEG
RNF39
ARHGEF25
GAMT
FNBP1
Q93YZ4
DIXDC1
DENND1C
NES
ATP1A1
DEFB1
LOC100289090
CLMN
CORO7
PDE9A
RNF19B
LIPE
LRRC8E
C6orf204
USP35
XLOC_005273
SGCA
LOC100128402
XLOC_l2_010602
LOC100129999
NEURL1B
CBX2
SPAG8
MICA
LOC100506268
LOC100128517
ATP1A3
LOC100289211
RPPH1
ACAP3
PLEKHO1
CCDC146
EPHB2
SPACA4
SERTAD2
ADARB1
LMBR1L
PMEPA1
KCNN4
SLC23A2
S1PR4
LOC619207
XLOC_013788

KIF21B
CELSR2
HIPK4
WDR47
LOC100508869
FAM73B
CCDC11
TTC21A
UBE2Q2P3
LOC100506124
IRGQ
HDAC10
PAGE2B
SH3BP5
TLE2
MYO1E
SAT2
LOC100129380
INPP5K
C22orf23
PLCXD1
XLOC_010194
ATP8B2
XLOC_013711
IQCD
LOC283887
RNF216
LOC100652768
C20orf96
ZDHHC11
OVGP1
MYLK2
FLJ30064
ADAM8
BLVRA
PALM
SPTSSB
XLOC_l2_006080
LAMA3
KLHL17
C17orf96
LOC100131829
LOC644242
COL28A1
MUSTN1
SLC26A11
RNF122
C18orf8

CX3CL1
EVI5L
XLOC_003854
C1orf226
TRIM52
C19orf51
XLOC_006994
ANKDD1A
XLOC_009146
PLEKHH2
SNHG12
MAPKBP1
SERTAD1
FGD6
ELF3
NR1H4
LOC100130713
XLOC_013905
LOC100616668
FAT4
CDRT1
LOC100128857
XLOC_l2_010118
LOC100652843
SRRM5
SSBP2
CCDC80
SEMA3G
SMAD7
H19
XLOC_008149
FKBP14
PDXP
C5AR1
LRRC15
MICALL2
LOC100130357
ITGB7
KCNK7
KCNN2
LRRC24
XLOC_l2_007204
SCARNA8
NFIL3
XLOC_009944
XLOC_l2_005553
PPARD
KCNC3

DGCR14
PFKFB2
GADD45A
XLOC_l2_011043
PTK2B
LOC100133669
SLC24A6
ARG2
PCYOX1L
GOLGA6L6
XLOC_l2_005517
IGSF8
XLOC_l2_003897
XLOC_006721
FGF22
ANKRD13D
CCDC96
RAD9A
LARP6
HLA-DOB
SNORD99
CPT1C
SH3PXD2A
AHRR
RAB36
TMCO2
ESPN
TIGD3
NPHS1
RAB44
DCDC2B
XLOC_l2_014421
XLOC_009797
TRIM25
SPIRE1
LOC100506766
HSPB11
FZD4
LOC100132077
NUAK2
MAPK8IP3
XLOC_013356
ERVK13-1
HTR7P1
FRMD6
LOC401317
LOC100289488
ZBP1

MAFF
SNORA43
PRICKLE3
FAM102A
PLA2G15
PRKCA
C4orf32
PLA2G4D
CSF1R
OR8G1
CGNL1
GAST
ASMTL
FAM90A1
C14orf43
ZDHHC20
ARNTL
BTNL8
RAPGEFL1
TTLL13
LAG3
C17orf69
XLOC_013732
RELL1
SGTB
XLOC_013712
CDKN2D
XLOC_010378
XLOC_012288
LZTS1
SKI
MBOAT2
LAMP3
C11orf94
PVR
AKNA
CCDC148
GNG3
OASL
CLDN6
C6orf132
XLOC_000055
XLOC_l2_015593
GDF1
XLOC_009191
DGAT1
SPTB
PTGER4

XLOC_010542
RASAL1
CXCL6
C4orf47
XLOC_009487
ZNF385C
CLEC4A
RPL23AP32
TUBB6
RNU2-2
ENTPD2
TMC8
LOC100131796
PLCE1
SERAC1
MGC4294
PNPLA3
FLRT1
ZMIZ2
SPSB1
GLB1L3
SGK223
GSDMB
LOC90246
SMPD1
XLOC_013772
ADAM32
LGI4
CFP
EFNB2
FAM90A7
LOC100506791
NFATC4
CDH3
GLRX
SEMA3F
PIGR
ARHGAP26
GRAMD1A
C17orf109
NDRG4
LOC388588
MSX2
XLOC_013368
SPRED3
HCN3
LOC286059
XLOC_l2_014820

PLEKHH3
XLOC_002132
FUT7
XLOC_002169
XLOC_l2_001537
XLOC_012568
ZNF697
XLOC_001048
JAG2
NOTCH3
XLOC_010989
XLOC_001243
LOC100133190
ATP6V0D1
NLGN2
LOC100506001
ITPKA
XLOC_009943
TAOK3
EMP1
XLOC_004693
DAPK3
SOCS1
XLOC_011284
RFPL2
PFKFB4
NKPD1
NCEH1
FAM71A
SNORA14A
ATG2A
PRSS53
IQUB
TPRN
VAMP1
XLOC_012343
XLOC_002345
LOC100128697
RAB6B
TNKS1BP1
PLEKHO2
FUT1
TRIM15
GRHL1
KANK3
SLC4A3
LOC100130276
XLOC_l2_012323

PMEL
CEACAM22P
MYOM1
RBM44
PIP4K2A
LOC645195
BCAS4
XLOC_000388
IZUMO1
IRAK2
LOC100505576
ZFHX2
ENPP5
XLOC_001133
LOC100652797
DENND2C
XLOC_000101
LHX1
XLOC_l2_000969
C12orf34
XLOC_l2_014504
PTGDS
C20orf195
HIST3H2BB
SIPA1
LGALS7
JAKMIP2
ATPAF1-AS1
GPR146
BTN2A2
XLOC_l2_012847
MGC50722
SNORA61
CYP46A1
ZNF253
LOC100507233
ANKRD29
ADM
LOC440934
NEU1
CYP2U1
EPAS1
XLOC_002821
SOAT1
XLOC_002997
LOC728978
PLEKHF1
XLOC_014219

SLC22A13
JAG1
SLC7A5
SMOX
CCDC28B
PLXNA3
SH2D1B
PLD2
RGS16
ZSWIM4
MYBL1
ARHGEF4
GRK4
CCDC89
PKP1
AHDC1
HLA-A
LCA5
RASSF4
NFKBIZ
LOC100506188
VSIG8
SLC25A41
XLOC_013370
OSM
GDPD5
PLK3
C15orf62
DZANK1
LOC200609
LOC100507109
HABP4
MITF
HKDC1
DOCK4
CRYM
LPHN1
ENO2
XLOC_011359
XLOC_006195
H1F0
RAB11FIP1
XLOC_013559
GABBR1
MPP2
CTIF
XLOC_003417
LOC100507347

CABLES2
KSR1
AMICA1
C8orf66
CLGN
LOC100129171
BMP6
SEMA4D
XLOC_008209
XLOC_013602
SYNGR3
SLC25A27
SLC45A4
AQP7P1
MSX1
NRGN
TSHZ2
XLOC_010245
XLOC_002069
TIPARP
JAK3
LOC100131581
POLN
SNTA1
XLOC_014369
TNFRSF25
EPOR
PPP1R15B
XLOC_012169
SLC29A4
SECTM1
TTLL6
SOBP
MEF2C
VWA5B2
NOS1AP
IL36G
LINC00312
UBTD1
LRP4
DMPK
RASA4
FBXO44
CAMTA1
ULBP2
FSTL3
XLOC_l2_003792
C9orf72

FAM126B
EHD1
CLIP2
ZDHHC11B
RHOF
METRNL
PRKCG
ACTA1
MERTK
XLOC_010591
C1orf201
ASNS
S1PR2
XLOC_l2_001134
TMEM25
GFOD1
XLOC_001338
SYCE2
NAP1L5
XLOC_013955
SPIRE2
KALRN
ASGR1
DNAH17
SLC25A25
COLEC11
FLT3LG
PDGFB
SPOCD1
GRASP
FILIP1L
SAMD14
DUSP5P
XLOC_011513
PREX1
IQCJ-SCHIP1
LMCD1
ABCA3
CPLX3
XLOC_002277
LILRB3
SORBS1
SEC14L4
LOC497257
CMTM2
IGSF9
CECR2
RELT

PPP1R14C
SLC6A13
LOC100288911
PTHLH
LOC100505573
FLJ26086
TSC22D3
CEBPB
XLOC_004557
PEG10
KLF4
SLC30A2
LOC151657
MFAP3L
ABCB9
XLOC_l2_003674
HMOX1
XLOC_011614
ELFN2
NEDD9
XLOC_002872
GPC2
TNNC1
CAPN5
RASIP1
XLOC_012567
PCK1
SPRR1A
LOC388242
RNASE1
ADRB1
LOC100507959
EIF5A2
IER5
CSRNP1
SPINK2
XLOC_l2_000804
LOC285147
MYZAP
XLOC_014402
LOC100507800
NUMBL
XLOC_013549
UCN2
TPST1
sept-03
ProSAPiP1
TCAP

LOC606724
ABHD8
XLOC_002231
LCAT
EPPK1
SLC1A4
EGFL7
CREB5
FAM18B2-CDRT4
FBXL16
XLOC_011287
SPOCK2
MAPRE3
EREG
AOC3
XLOC_001788
GLIS3-AS1
FLYWCH1
C7orf63
TRIM36
TOM1L2
LOC100508950
FAM65C
SH3PXD2B
C3orf45
MSX2P1
AASS
XLOC_l2_007427
CD274
PTGIR
TIMP2
XLOC_008488
BMP2
LINC00163
TNFRSF10B
DNAH12
GBX2
XLOC_011331
MAP1A
XLOC_007504
SYT11
WWC2
BAIAP2
C3
GDF15
XLOC_000695
XLOC_000478
CILP2

ETNK2
GNG4
RHEBL1
VKORC1
RPS6KA2
XG
BCORL1
DUSP8
CTAG1A
IL17RD
IL21R
TMEM35
PTGS1
ITGA7
XLOC_013093
ENTPD8
KLHL25
CDON
ERN1
XLOC_010275
XLOC_010743
CD19
C16orf79
LOC100129119
XLOC_002296
KIAA0509
BATF3
RFPL3-AS1
GSTM2
DBP
PDLIM7
CDKN1C
SLC4A11
XLOC_002650
RCAN2
LOC100287415
PNPLA1
DENND3
CALHM3
GPR111
HPX
ASAP3
LOC100131043
XLOC_l2_013153
LOC100131551
ERRFI1
XLOC_l2_014191
NBEAP1

C14orf49
C2orf53
XLOC_002918
GATSL3
XLOC_011348
CD52
CYP2E1
CTSL1P2
XLOC_l2_007783
MYO1G
AVPI1
XLOC_005747
BCL3
XLOC_l2_002355
OGDHL
XLOC_009880
OSBP2
EXT1
AREG
XLOC_003016
PDE4A
ARID3A
BMP8A
C2CD2L
MUC21
CROCCP3
IL11RA
XLOC_l2_004631
ATP8B3
METTL20
CDR2L
TMEM86A
ITPRIP
LOC100507445
CNNM4
FLJ44124
MAK
SCHIP1
FNDC4
IER3
TOX2
Q7VLF4
CYP2G1P
ADAMTS7
XLOC_002732
GNAZ
GNG8
CD7

LOC100130157
CCDC136
XLOC_010670
LOC400548
ZP2
RHOB
GCGR
XLOC_002515
TTC25
ZCCHC24
XLOC_l2_006812
IDS
P2RX7
TNFRSF4
MCF2L
TRIM46
XLOC_l2_012942
CARD14
PI4KAP1
FAM69B
LRRC6
GP6
PTGES
RORA
CFD
FTCD
DKFZp686M1136
SRRM3
ST3GAL5
EID3
C8orf31
PPP1R15A
XLOC_009196
TBC1D10C
ABLIM2
XLOC_004584
EEF1DP3
FLJ42392
XLOC_014336
TEX19
MAP3K14
EGR1
CD83
XLOC_l2_006944
LOC400927
MFNG
LOC100132474
GARNL3

LINC00051
C2orf57
ATAD3C
BDKRB1
PPP2R5B
SLC45A1
FLJ23152
GJD3
SLC25A34
FXYD4
MYT1L
KRT16P2
PLA2G4E
RFPL4A
KRT83
MGC23284
EFEMP2
XLOC_013776
DLX1
TAS1R3
XLOC_l2_015752
XLOC_l2_013256
KRT80
LOC100506252
FLJ41484
CLDN17
ADCY9
SNPH
XLOC_002523
GPR155
XLOC_001462
LOC157562
LOC441268
IRF1
KCNJ18
AKAP2
PHACTR3
XLOC_l2_013500
SIRT4
XLOC_005784
FAM183A
RELB
TMEM63C
C17orf72
CHKA
RTN2
PRRT2
CCR10

XLOC_007598
ADORA2A
GLIPR2
WISP2
CADM4
CXorf59
ZIC3
RCOR2
XLOC_000856
NOX5
GJA3
MAN1C1
RASGRP2
PACSIN3
TBXA2R
PIM1
XLOC_014018
TMEM158
SLC4A9
MGC15885
LOC100505619
DKFZp451A211
SBSN
FOS
CAMK2N2
NFKBIA
NFKB2
BIK
SLC38A4
CCDC151
XLOC_002322
LOC100288432
XLOC_011827
ARID5A
XLOC_l2_012552
NACAD
XLOC_011081
DMBX1
FAM46C
ZSWIM6
ABTB2
FGF1
ARID3B
XLOC_l2_010386
CITED4
XLOC_012209
GDPD3
XLOC_l2_008760

CYP2F1
SH2D5
LOC286114
XLOC_l2_015265
LOC100127983
XLOC_000545
LOC729040
RAB42
ARAP3
XLOC_004598
OLFML2A
LINC00087
XLOC_004325
HIC1
VEGFA
XLOC_001313
RNF24
DDIT4
XLOC_011559
HAL
SESN2
ATP1B2
PTAFR
POU2F2
PLIN4
ABCG1
SPEF1
DYRK3
KIAA1683
DIRAS3
RGS11
SHANK3
CPEB4
WFIKKN1
SH2B2
APOE
GGT8P
TDRD6
LMTK3
KLK7
ITIH4
KLHL32
PPM1J
SCN3B
XLOC_008989
XLOC_013535
LOC283403
NGFR

MOG
RNU11
DFNB31
ZNF204P
LOC728208
ICAM5
PER1
C8orf60
XLOC_l2_009332
LCN6
LOC399708
GK2
SELM
TUFT1
UNC5B
PADI3
C10orf129
XLOC_l2_011987
C20orf152
LOC100653515
XLOC_009810
LOC254896
LRRC9
CBX4
XLOC_000220
XLOC_010933
HAS3
BRSK2
PXK
BMF
LOC100652839
LOC340335
PPP1R14D
GPRC5B
XLOC_000111
CD1E
PLA2G2F
CATSPERB
BAIAP3
XLOC_006495
XLOC_006951
EGR2
XLOC_006421
LOC283404
RDH12
VAV1
MTMR7
HBA2

LOC100505697
MICALCL
GATA5
XLOC_005763
PP14571
LOC100653007
LOC100505583
ARHGAP30
TNFRSF10C
SLC9A1
LOC100507131
CERCAM
HBEGF
CYR61
SLC25A42
XLOC_000208
TUBB3
HSFY2
PGF
MAP1LC3A
DSG3
XLOC_l2_000018
XLOC_l2_009292
LRRC10B
SDC4
RNF112
BAI2
NEBL
XLOC_007835
Q29HP5
LOC100131355
PDE2A
MATN4
XLOC_l2_002351
DUSP1
SCARF2
CDH15
LOC100507930
C1orf130
CYTH3
LINC00032
KLHL29
PPFIA4
LOC100499221
XLOC_006150
CCDC65
DUSP10
LOC100507039

MYBPHL
XLOC_l2_000116
LOC283143
LYST
XLOC_007191
PTPRU
NFKBIE
OSBPL6
XLOC_011047
KLRD1
XLOC_l2_000735
XLOC_010942
DLX3
LYPD5
ADM2
HES4
XLOC_002688
FKBP1B
DBNL
KEL
ADCY4
IL8
NPW
C17orf99
WNT5B
CLTCL1
WDR69
SERPINI1
TFCP2L1
FGF21
FCHO1
ANKRD34A
IL32
GPR83
FHAD1
RAB3IL1
KLF9
BBC3
CDKN2B
KRT12
SERTM1
MOGAT1
LOC100505869
CERS1
KRTAP1-3
CTRC
XLOC_007189
TMCC2

PEAR1
TNFAIP2
CD84
CSRP2
ARL4C
FP588
AHNAK2
DPPA2
ZBTB46
PRPH
SUSD3
XLOC_003130
DDIT3
SYT12
EFNA3
TRPV6
XLOC_l2_003737
FOSB
LOC728228
REEP2
STOM
MIOX
NRTN
SH2B3
CCR7
RAB3A
LYL1
ABAT
AGPAT4-IT1
ESPNL
DHDH
N4BP3
ANKRD35
PPP4R4
LOC100507162
VWCE
LOC283674
PRDM1
SNAI3
CHST6
ULBP1
MPP1
CHD5
ADRA2B
XLOC_011080
LOC100506662
GP1BB
DLK2

KRT42P
FBXO2
TMEM169
GPSM1
ASB2
XLOC_009601
HAPLN3
ARL4D
KRT86
PIP5KL1
DPF1
TNFRSF18
CXCL3
TMEM151A
APC2
HSPA12A
CYTH4
COL7A1
LINC00319
TMOD2
LOC100507002
GPR37L1
XLOC_001366
ZMYND10
SCAMP5
CORO1A
ELOVL3
USP2
NAT8L
XLOC_002878
CSF2
SLC19A3
XLOC_005991
RGL1
DLX2
HEG1
UAP1L1
LOC100506120
CDY2A
KCNQ1OT1
XLOC_014226
ATF3
IL11
XLOC_l2_006036
BPIFB1
sept-06
ASPHD2
XLOC_009167

C3orf32
DUSP5
KRT14
ARC
MAP3K12
HYAL1
RASL10A
SERPINE1
DPF3
WBSCR28
LOC284570
XLOC_l2_006021
C11orf86
FAM43A
PBX4
TNFRSF9
SRCIN1
XLOC_008611
CCK
LGALS1
C1QL4
ICAM1
RHOV
IL1A
IFITM10
SMARCD3
VPS37D
KIAA0513
SERPINB9
LOC643037
SCN4B
NXPH4
GADD45B
HES7
RIMS3
XLOC_l2_011908
IL4I1
XLOC_013743
VASN
RND1
KRT17
MLXIPL
SYNPO
TUBB2B
DHH
CDKN1A
NTN5
GEM

GPR20
HOXC12
LYPD3
MICB
CHAC1
FAM46B
C9orf169
LRRC25
BEST1
SNAI1
TNFSF9
XLOC_004244
PLIN5
BDKRB2
SOCS3
LOC93463
KLC3
LOC100288175
CBS
NR4A3
NEURL3
CXCL2
EDN2
PLK5
CXCL1
ADC
TNFAIP3
LOC731779
TMEM121
SLC2A4
BIRC3
LOC100506546
XLOC_000027
ABCG4
IL10RA
FSCN1
RRAD
COL1A1
KREMEN2
IL1B
ADRB2
JSRP1
ICAM4
DACT3
CEND1
SEMA7A
IL17C
CYP1A1

CREB3L3
TNF
SH2D3C
CCL20
ANGPTL4
