# Supplementary material for: The probiotic Propionibacterium freudenreichii as a new adjuvant for TRAIL-based therapy in colorectal cancer
Source: Oncotarget. 2016 Jan 11;7(6):7161–78. doi: 10.18632/oncotarget.6881 (PMC4872776; doi:10.18632/oncotarget.6881)
Supplement: Supplementary file 3 [file oncotarget-07-7161-s003.pdf]

Supplementary Table 2 :

TRAIL-SN		TRAIL-C3/C2		TRAIL	
**KEGG ID**	**adj_pvalue**	**KEGG ID**	**adj_pvalue**	**KEGG ID**	**adj_pvalue**
hsa04060	7,88E-11	hsa04010	1,12E-19	hsa04060	4,09E-15
hsa05200	1,48E-10	hsa04360	3,41E-13	hsa04621	1,68E-07
hsa04010	1,48E-10	hsa04020	1,63E-12	hsa05200	4,48E-06
hsa00562	1,48E-10	hsa05200	3,19E-12	hsa04630	2,23E-05
hsa04070	4,02E-10	hsa04060	9,40E-11	hsa04640	2,37E-05
hsa04360	2,20E-08	hsa04510	5,12E-10	hsa04620	4,22E-05
hsa04020	6,82E-08	hsa04916	1,83E-09	hsa04062	7,50E-05
hsa04340	7,15E-08	hsa04062	5,92E-09	hsa04210	1,37E-04
hsa05217	1,07E-07	hsa04512	5,92E-09	hsa04622	4,09E-04
hsa04810	4,26E-07	hsa00562	1,28E-08	hsa04650	8,30E-04
hsa04920	8,97E-07	hsa04660	2,91E-08	hsa04623	1,45E-03
hsa04512	8,97E-07	hsa04080	3,34E-08	hsa05222	1,64E-03
hsa04916	1,04E-06	hsa04070	3,34E-08	hsa04660	3,17E-03
hsa04670	1,15E-06	hsa04670	3,36E-08	hsa05120	3,65E-03
hsa04930	1,95E-06	hsa05217	3,92E-08	hsa05219	4,60E-03
hsa04510	1,95E-06	hsa04810	5,19E-08	hsa04010	4,96E-03
hsa04912	3,57E-06	hsa04144	8,43E-08	hsa04514	8,02E-03
hsa04640	6,07E-06	hsa04722	1,88E-07	hsa05218	2,59E-02
hsa04960	6,08E-06	hsa04115	5,33E-07	hsa04920	2,59E-02
hsa04062	1,32E-05	hsa04340	7,32E-07	hsa04662	2,73E-02
hsa04630	1,32E-05	hsa04664	7,91E-07	hsa05220	3,02E-02
hsa00564	1,34E-05	hsa04912	1,41E-06	hsa04360	3,02E-02
hsa04270	1,90E-05	hsa04960	1,50E-06	hsa04672	3,82E-02
hsa04910	1,98E-05	hsa04662	1,50E-06	hsa05340	3,82E-02
hsa04144	3,90E-05	hsa04150	2,05E-06	hsa05215	4,35E-02
hsa04080	4,00E-05	hsa04930	2,77E-06	hsa04512	4,71E-02
hsa05410	4,00E-05	hsa04012	2,90E-06	hsa00740	4,74E-02
hsa00561	4,04E-05	hsa04920	2,90E-06		
hsa04664	4,04E-05	hsa04310	5,30E-06		
hsa00532	6,80E-05	hsa04666	6,69E-06		
hsa04666	6,96E-05	hsa04270	7,95E-06		
hsa04310	8,04E-05	hsa05414	1,71E-05		
hsa04710	9,50E-05	hsa04910	1,71E-05		
hsa00051	1,20E-04	hsa00230	2,29E-05		
hsa00533	1,28E-04	hsa04914	2,29E-05		
hsa04012	1,30E-04	hsa00330	2,99E-05		
hsa00260	1,98E-04	hsa04370	3,86E-05		
hsa04722	2,93E-04	hsa05221	4,90E-05		
hsa04530	3,01E-04	hsa05410	4,90E-05		
hsa05219	3,01E-04	hsa05219	4,90E-05		
hsa05414	4,44E-04	hsa00590	5,56E-05		
hsa04520	5,15E-04	hsa00564	5,85E-05		

hsa04115	5,73E-04	hsa04110	5,97E-05
hsa05130	6,07E-04	hsa05211	5,97E-05
hsa05120	7,63E-04	hsa00380	6,95E-05
hsa00565	7,83E-04	hsa04350	6,95E-05
hsa03320	9,97E-04	hsa04710	7,91E-05
hsa04150	9,97E-04	hsa04530	7,97E-05
hsa04540	1,03E-03	hsa05215	8,38E-05
hsa04660	1,03E-03	hsa00260	1,94E-04
hsa04210	1,03E-03	hsa04114	1,97E-04
hsa05412	1,09E-03	hsa05218	1,97E-04
hsa00510	1,25E-03	hsa00532	2,30E-04
hsa04142	1,30E-03	hsa04210	2,88E-04
hsa05221	1,31E-03	hsa00410	2,90E-04
hsa00600	1,55E-03	hsa05120	2,90E-04
hsa04350	1,83E-03	hsa04540	2,90E-04
hsa04662	2,28E-03	hsa00533	2,90E-04
hsa04620	2,44E-03	hsa05214	2,90E-04
hsa04610	2,44E-03	hsa04630	3,11E-04
hsa04730	2,44E-03	hsa04640	3,51E-04
hsa04650	2,44E-03	hsa04730	3,82E-04
hsa05340	3,72E-03	hsa04610	3,95E-04
hsa05222	3,91E-03	hsa00051	4,79E-04
hsa00250	3,96E-03	hsa05222	4,79E-04
hsa02010	3,96E-03	hsa04620	6,68E-04
hsa00330	3,96E-03	hsa04330	6,71E-04
hsa04330	3,96E-03	hsa05020	7,13E-04
hsa00910	4,11E-03	hsa00120	8,94E-04
hsa00534	4,11E-03	hsa00770	9,00E-04
hsa05218	4,39E-03	hsa00760	1,03E-03
hsa00380	4,87E-03	hsa00450	1,03E-03
hsa05215	5,75E-03	hsa04320	1,03E-03
hsa00120	7,14E-03	hsa00270	1,49E-03
hsa00590	7,14E-03	hsa00600	1,56E-03
hsa00601	8,49E-03	hsa00250	2,00E-03
hsa04514	8,69E-03	hsa00531	2,00E-03
hsa04370	8,94E-03	hsa04621	2,07E-03
hsa00531	1,14E-02	hsa05130	2,07E-03
hsa00563	1,14E-02	hsa05412	2,23E-03
hsa00760	1,24E-02	hsa04520	2,27E-03
hsa05210	1,35E-02	hsa04720	2,27E-03
hsa05211	1,43E-02	hsa00561	2,30E-03
hsa04320	1,43E-02	hsa04142	2,49E-03
hsa00020	1,71E-02	hsa05210	2,81E-03
hsa04720	1,79E-02	hsa04650	2,92E-03
hsa00450	1,98E-02	hsa04742	3,23E-03
hsa00052	2,00E-02	hsa05014	3,23E-03
hsa00750	2,07E-02	hsa00510	3,26E-03
hsa00511	2,13E-02	hsa00565	3,32E-03

hsa05214	2,15E-02	hsa03320	3,32E-03
hsa04621	2,54E-02	hsa03450	3,32E-03
hsa00010	2,64E-02	hsa04130	3,32E-03
hsa05014	2,73E-02	hsa00130	3,32E-03
hsa05213	2,81E-02	hsa00430	3,32E-03
hsa04260	2,99E-02	hsa05223	3,97E-03
hsa03450	3,33E-02	hsa00534	3,99E-03
hsa00730	3,33E-02	hsa05212	4,68E-03
hsa00740	3,33E-02	hsa00511	6,10E-03
hsa00400	3,33E-02	hsa00480	6,52E-03
hsa00130	3,33E-02	hsa04622	6,52E-03
hsa04742	3,36E-02	hsa02010	7,32E-03
hsa04130	3,36E-02	hsa04260	7,32E-03
hsa04622	3,36E-02	hsa00030	7,38E-03
hsa04114	3,42E-02	hsa05213	7,61E-03
hsa00340	3,89E-02	hsa00360	7,74E-03
hsa00360	4,21E-02	hsa00601	7,74E-03
hsa00980	4,24E-02	hsa00591	7,74E-03
hsa04914	4,63E-02	hsa00563	8,37E-03
hsa00592	4,71E-02	hsa00460	8,37E-03
hsa00100	4,71E-02	hsa04514	1,05E-02
		hsa05220	1,06E-02
		hsa00910	1,11E-02
		hsa00400	1,16E-02
		hsa00020	1,20E-02
		hsa00300	1,34E-02
		hsa05340	1,34E-02
		hsa00785	1,34E-02
		hsa00280	1,65E-02
		hsa00100	1,69E-02
		hsa00592	1,69E-02
		hsa00620	1,76E-02
		hsa00512	1,87E-02
		hsa00071	2,58E-02
		hsa04623	2,63E-02
		hsa00830	2,64E-02
		hsa04950	2,64E-02
		hsa03410	2,78E-02
		hsa00340	2,92E-02
		hsa00740	2,92E-02
		hsa00970	2,92E-02
		hsa00604	2,92E-02
		hsa00980	3,25E-02
		hsa05110	3,76E-02
		hsa00350	3,96E-02
		hsa00730	3,96E-02
		hsa00640	4,29E-02
		hsa00052	4,29E-02

hsa04120	4,29E-02
hsa00010	4,55E-02
hsa00630	4,55E-02
hsa00790	4,55E-02
hsa00920	4,60E-02

SN		C3/C2	
**KEGG ID**	**adj_pvalue**	**KEGG ID**	**adj_pvalue**
hsa04010	2,70E-11	hsa00562	9,63E-06
hsa04360	1,08E-10	hsa04360	1,20E-04
hsa05200	9,07E-09	hsa04020	1,20E-04
hsa04020	9,07E-09	hsa05217	1,20E-04
hsa04510	9,07E-09	hsa04340	1,20E-04
hsa04340	9,07E-09	hsa04070	1,20E-04
hsa04060	2,68E-08	hsa04060	2,47E-04
hsa00562	5,20E-08	hsa04916	2,43E-03
hsa05217	1,30E-07	hsa04010	3,52E-03
hsa04070	1,30E-07	hsa00590	4,34E-03
hsa04930	1,77E-07	hsa04640	4,52E-03
hsa04512	1,08E-06	hsa04144	5,15E-03
hsa04920	1,79E-06	hsa05200	5,56E-03
hsa00260	3,75E-06	hsa04062	1,17E-02
hsa04270	4,78E-06	hsa04930	1,31E-02
hsa04916	6,84E-06	hsa04960	1,54E-02
hsa04910	6,84E-06	hsa00564	1,58E-02
hsa04810	7,99E-06	hsa00330	1,58E-02
hsa04144	1,54E-05	hsa04666	1,76E-02
hsa04912	1,54E-05	hsa00260	1,82E-02
hsa04670	1,54E-05	hsa04664	1,82E-02
hsa04664	2,16E-05	hsa04270	1,90E-02
hsa04310	2,16E-05	hsa04080	1,90E-02
hsa04062	2,53E-05	hsa04920	2,09E-02
hsa00564	3,57E-05	hsa04310	2,46E-02
hsa04960	4,41E-05	hsa00532	2,46E-02
hsa00532	8,35E-05	hsa04650	2,46E-02
hsa04630	1,15E-04	hsa00565	2,54E-02
hsa05410	1,29E-04	hsa04660	2,91E-02
hsa04640	1,46E-04	hsa00980	2,93E-02
hsa04080	2,05E-04	hsa04710	3,35E-02
hsa04012	2,51E-04	hsa04910	3,63E-02
hsa00051	2,54E-04	hsa03320	4,36E-02
hsa04370	2,69E-04	hsa04912	4,36E-02
hsa04730	2,69E-04	hsa04150	4,36E-02
hsa00330	2,97E-04	hsa04670	4,55E-02
hsa04530	2,97E-04	hsa04370	4,55E-02
hsa05414	5,51E-04	hsa04510	4,56E-02
hsa04142	5,97E-04	hsa00830	4,56E-02
hsa04722	7,41E-04	hsa04115	4,93E-02
hsa04540	8,35E-04		
hsa04115	9,47E-04		

hsa00565	1,46E-03
hsa04710	1,46E-03
hsa04666	1,64E-03
hsa05222	1,69E-03
hsa04210	1,71E-03
hsa04660	2,14E-03
hsa05130	2,21E-03
hsa04350	2,32E-03
hsa04330	2,32E-03
hsa00380	2,58E-03
hsa04520	2,72E-03
hsa00561	2,72E-03
hsa04662	2,72E-03
hsa00531	3,00E-03
hsa05219	3,07E-03
hsa04150	3,62E-03
hsa00250	4,75E-03
hsa00910	5,13E-03
hsa05014	7,29E-03
hsa00533	7,35E-03
hsa05412	7,36E-03
hsa00511	8,84E-03
hsa04720	9,01E-03
hsa00590	9,01E-03
hsa05120	1,16E-02
hsa04620	1,27E-02
hsa04514	1,27E-02
hsa04114	1,29E-02
hsa04610	1,29E-02
hsa03320	1,33E-02
hsa05210	1,33E-02
hsa00534	1,33E-02
hsa05214	1,35E-02
hsa00410	1,37E-02
hsa04650	1,40E-02
hsa05221	1,60E-02
hsa00360	1,60E-02
hsa04320	1,60E-02
hsa05211	1,62E-02
hsa02010	2,03E-02
hsa05218	2,03E-02
hsa00592	2,17E-02
hsa00750	2,25E-02
hsa04614	2,34E-02
hsa00510	2,39E-02
hsa00010	2,43E-02
hsa05213	2,45E-02
hsa04914	2,70E-02

hsa00600	3,15E-02
hsa00563	3,15E-02
hsa00120	3,62E-02
hsa00052	3,62E-02
hsa00400	3,62E-02
hsa00130	3,62E-02
hsa05215	3,94E-02
hsa00020	4,43E-02
hsa00601	4,81E-02
hsa04130	4,91E-02
